# Supplementary material for: Functional Divergence of Delta and Mu Opioid Receptor Organization in CNS Pain Circuits
Source: Neuron. 2018 Apr 4;98(1):90–108.e5. doi: 10.1016/j.neuron.2018.03.002 (PMC5896237; doi:10.1016/j.neuron.2018.03.002)
Supplement: Document S1. Figures S1–S9 [file mmc1.pdf]

Neuron, Volume *98*

Supplemental Information

Functional Divergence of Delta and Mu Opioid Receptor Organization in CNS Pain Circuits

Dong Wang, Vivianne L. Tawfik, Gregory Corder, Sarah A. Low, Amaury François, Allan I. Basbaum, and Grégory Scherrer

SUPPLEMENTARY FIGURES AND FIGURE LEGENDS

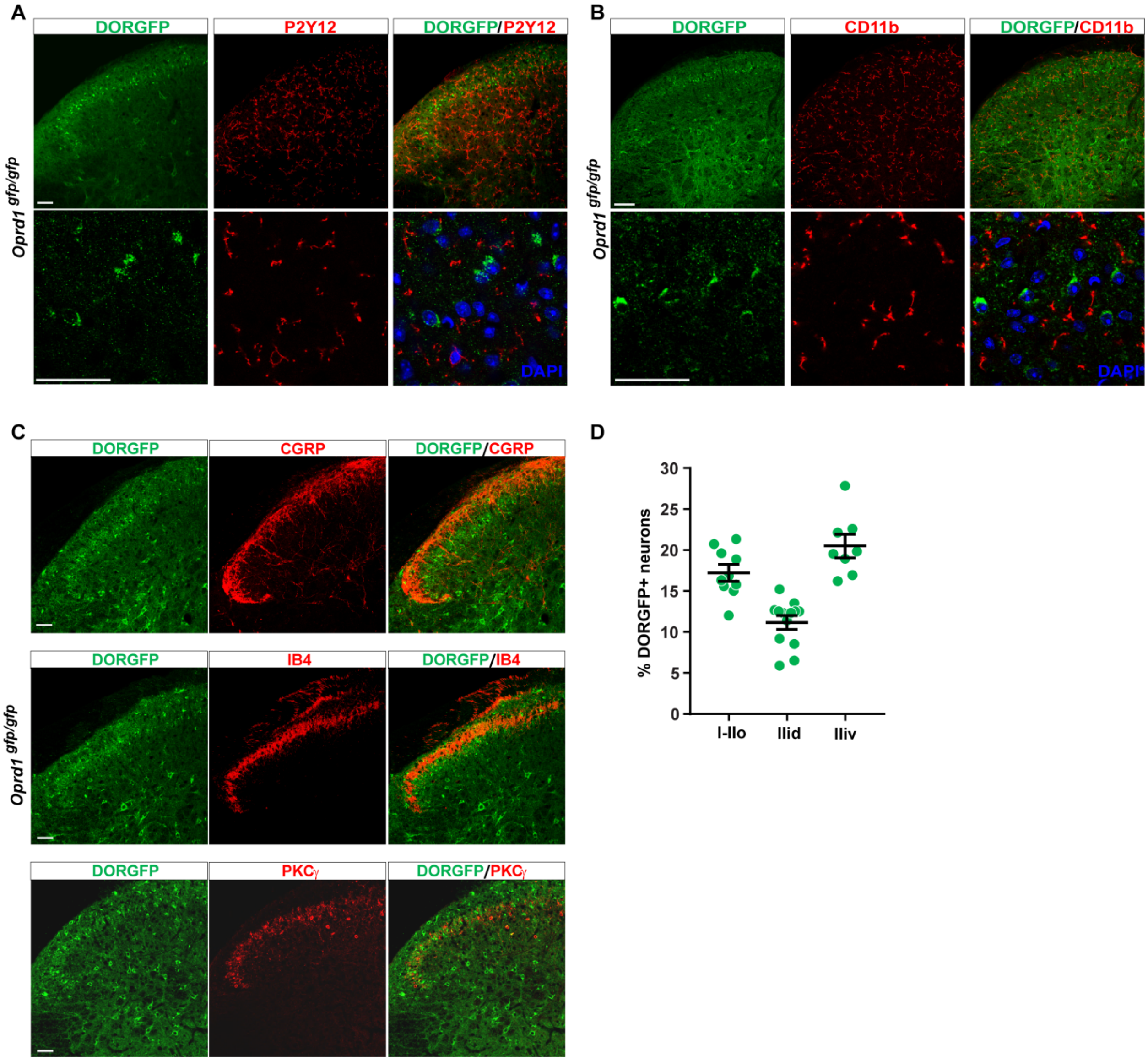

Figure S1

Figure S1. DORGFP Mouse Reports DOR Expression in Spinal Cord.

(Related to Figure 1)

(A-B) DOR is absent in spinal cord astrocytes and microglia by co-staining with anti-GFP and -P2Y12 or -CD11b antibodies.

(C) DORGFP+ neuron distribution in the dorsal horn.

Upper: Double labeling of GFP and CGRP in sections from DORGFP mice shows that 17.2% of DOR+ neurons are located in lamina I and II outer.

Middle: 11.2% of DOR+ neurons are located in lamina II inner dorsal within the IB4+ band.

Bottom: 20.5% of DOR+ neurons are located in lamina II inner ventral within the PKCγ+ band.

(D) Quantification of (C). Data are presented as mean ± SEM with dots showing individual counts in spinal cord sections from 3 mice.

Scale bars represent 50 μM.

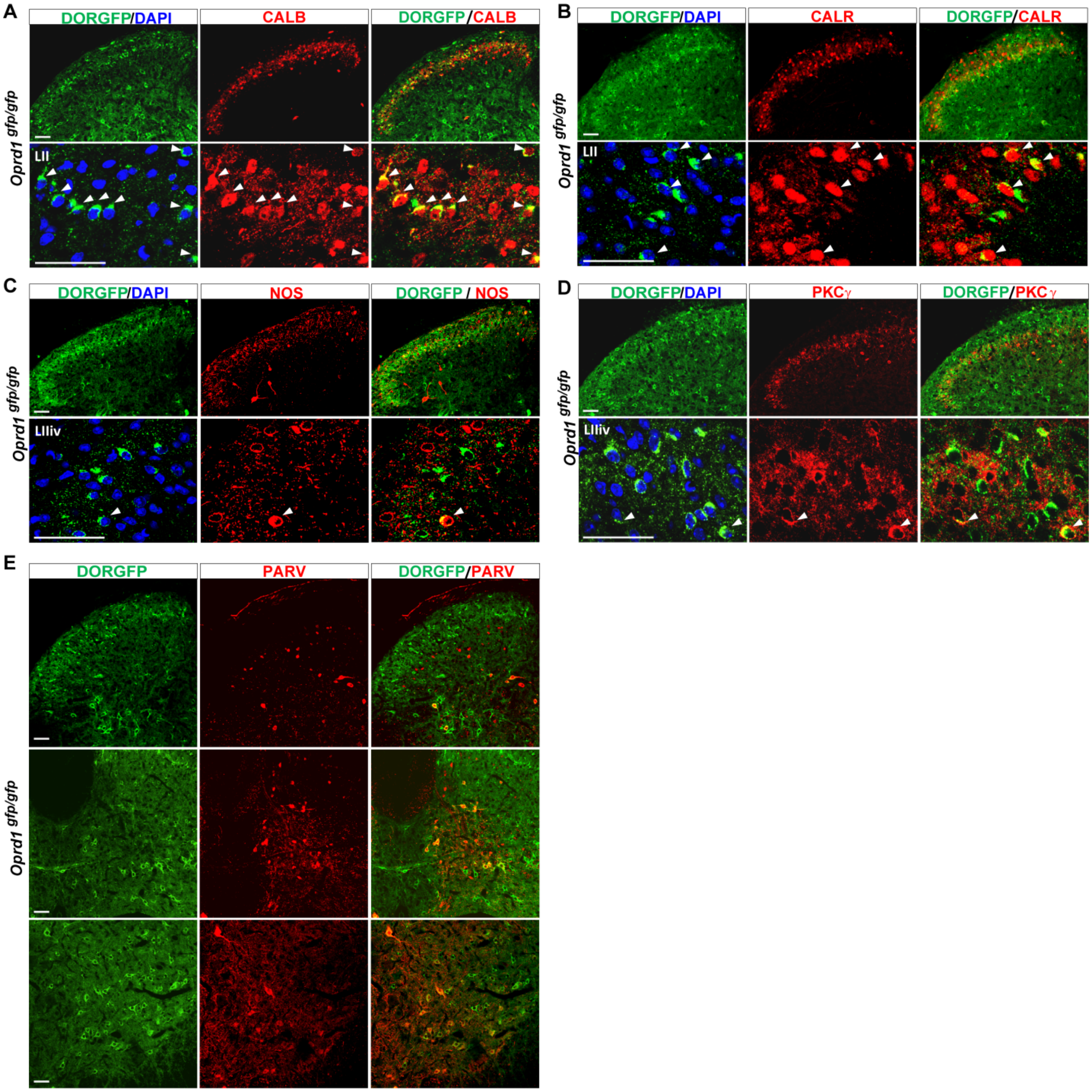
A
DORGFP/DAPI
CALB
DORGFP/CALB
Oprd1 gfp/gfp
LII
B
DORGFP/DAPI
CALR
DORGFP/CALR
LII
C
NOS
DORGFP / NOS
LIIiv
D
PKCγ
DORGFP/PKCγ
E
DORGFP
PARV
DORGFP/PARV

Figure S2

Figure S2. DORGFP+ Neurons in the Dorsal Horn Express Markers of Excitatory Interneurons.

(Related to Figure 2)

(A) CALB is expressed by 50.7% of DORGFP+ neurons. White arrowheads indicate cells where co-staining occurs.

(B) CALR is present in 26.5% of DORGFP+ neurons. White arrowheads indicate DORGFP+ CALR+ neurons.

(C) NOS is expressed by 12.1% of DORGFP+ neurons. White arrowheads indicate neurons that co-express DORGFP and NOS.

(D) PKCγ is present in 6.9% of DORGFP+ neurons. White arrowheads show DORGFP+ PKCγ+ neurons.

(E) DOR is absent in dorsal horn lamina III PARV+ neurons (top row) but is expressed by PARV+ neurons in the deep DH laminae IV-V (middle row) and by PARV+ neurons in the ventral horn (bottom row).

Scale bars represent 50 μM.

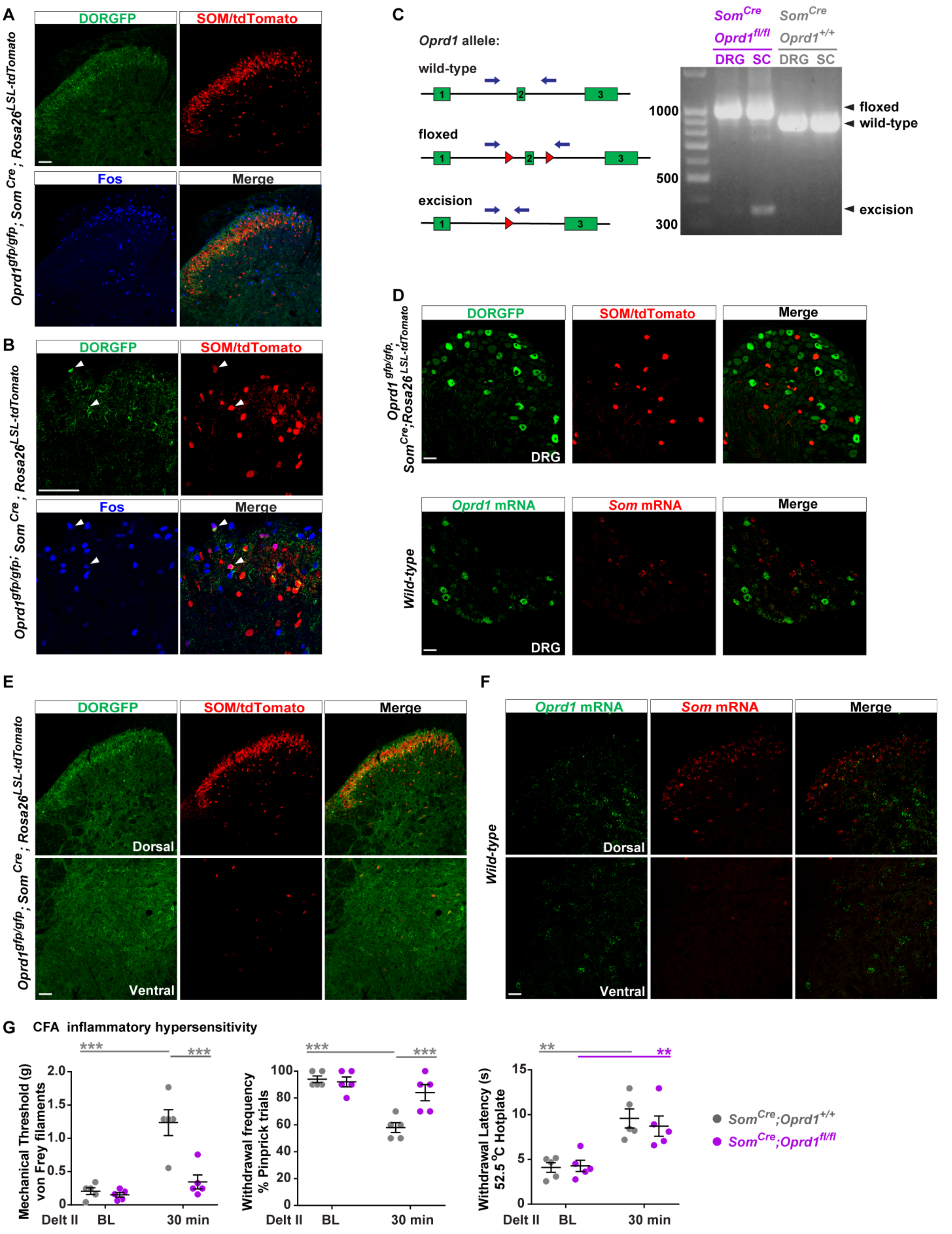
A
DORGFP
SOM/tdTomato
Fos
Merge
Oprd1gfp/gfp; SomCre; Rosa26LSL-tdTomato
B
DORGFP
SOM/tdTomato
Fos
Merge
Oprd1gfp/gfp; SomCre; Rosa26LSL-tdTomato
C
Oprd1 allele:
wild-type
floxed
excision
SomCre Oprd1fl/fl
SomCre Oprd1+/+
DRG SC DRG SC
1000
500
300
floxed
wild-type
excision
D
DORGFP
SOM/tdTomato
Merge
Oprd1gfp/gfp; SomCre;Rosa26LSL-tdTomato
DRG
Oprd1 mRNA
Som mRNA
Merge
Wild-type
DRG
E
DORGFP
SOM/tdTomato
Merge
Dorsal
Ventral
Oprd1gfp/gfp; SomCre; Rosa26LSL-tdTomato
F
Oprd1 mRNA
Som mRNA
Merge
Dorsal
Ventral
Wild-type
G
CFA inflammatory hypersensitivity
Mechanical Threshold (g) von Frey filaments
Withdrawal frequency % Pinprick trials
Withdrawal Latency (s) 52.5 °C Hotplate
Delt II BL 30 min
SomCre;Oprd1+/+
SomCre;Oprd1fl/fl

Figure S3

Figure S3. DOR in Spinal SOM+ Neurons Specifically Regulates Mechanical Pain.

(Related to Figure 3)

(A) Noxious mechanical stimulation of the hindpaw of *Oprd1*$^{gfp/gfp}$*;Som*^{Cre}*;Rosa26*$^{LSL\text{-}tdTomato}$ mice mice induced Fos expression in some DORGFP+ SOM+ dorsal horn neurons.

(B) High magnification images from (A). White arrowheads indicate DORGFP+ SOM+ neurons expressing Fos.

(C) PCR showing the recombination of the floxed allele and selective deletion of *Oprd1* in the spinal cord, but not DRG of *Som*^{Cre}; *Oprd1*$^{fl/fl}$ mice .

(D) Double labeling of GFP and tdTomato in sections from *Oprd1*$^{gfp/gfp}$; *Som*^{Cre}; *Rosa26*$^{LSL\text{-}tdTomato}$ mice shows that DOR is absent from SOM+ neurons in DRG neurons. Bottom: Fluorescence in situ hybridization experiments in wild-type mice confirm that *Oprd1* mRNA is absent from SOM+ DRG neurons.

(E) SOM is rarely present in spinal cord ventral horn neurons.

(F) Fluorescence in situ hybridization experiments in wild-type mice confirm that *Som* mRNA is rarely present in the spinal cord ventral horn.

(G) Decreased effect of intrathecal deltorphin II (15 μg) against CFA-induced inflammatory mechanical but not thermal hypersensitivity in DOR cKO mice. Data are presented as mean ± SEM . **, $P < 0.01$, ***, $P < 0.001$, repeated measures, Two-way ANOVA + Bonferroni. Von Frey threshold, pinprick and hotplate tests: n=5 control mice and 5 DOR cKO mice.

Scale bars represent 50 μM.

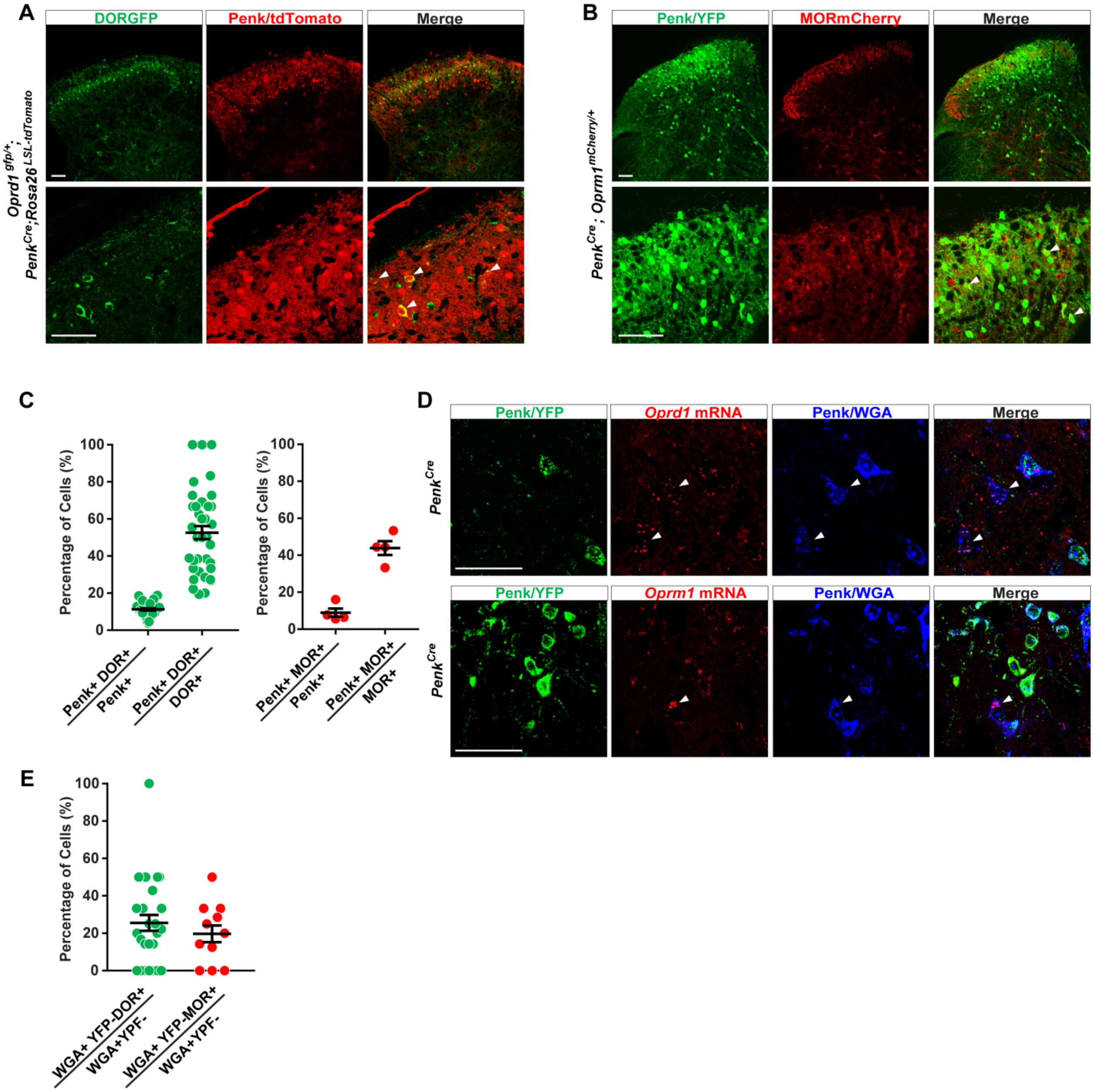
A
DORGFP
Penk/tdTomato
Merge
Oprd1 gfp/+; PenkCre;Rosa26LSL-tdTomato
B
Penk/YFP
MORmCherry
Merge
PenkCre; Oprm1mCherry/+
C
Percentage of Cells (%)
Penk+ DOR+ / Penk+
Penk+ DOR+ / DOR+
Penk+ MOR+ / Penk+
Penk+ MOR+ / MOR+
D
Penk/YFP
Oprd1 mRNA
Penk/WGA
Merge
Oprm1 mRNA
PenkCre
E
Percentage of Cells (%)
WGA+ YFP-DOR+ / WGA+YPF-
WGA+ YFP-MOR+ / WGA+YPF-

Figure S4

Figure S4. Spinal Neurons Expressing DOR or MOR Receive Synaptic Input from Enkephalinergic Neurons.

(Related to Figure 3 and Figure 5)

(A) Double labeling of GFP and tdTomato in sections from *Oprd1*$^{gfp/+}$;*Penk*^{Cre};*Rosa26*$^{LSL\text{-}tdTomato}$ mice shows that DOR is expressed by the Penk+ spinal dorsal horn neurons. White arrowheads indicate DORGFP+ Penk+ neurons.

(B) Co-staining of YFP and mCherry in sections from *Penk*^{Cre};*Oprm1*$^{mCherry+}$ mice shows that MOR is expressed by the Penk+ spinal dorsal horn neurons. White arrowheads indicate MORmCherry+ Penk+ neurons.

(C) Quantification of (A) and (B).

(D) Wheat germ agglutinin (WGA) anterograde tracing from Penk+ neurons and fluorescent in situ hybridization experiments in *Penk*^{Cre} mice indicates both DOR+ (upper panel) and MOR+ neurons (bottom panel) receive synaptic input from Penk+ spinal neurons.

(E) Quantification of (D).

Data are presented as mean ± SEM in (C) and (E). Scale bars represent 50 μM.

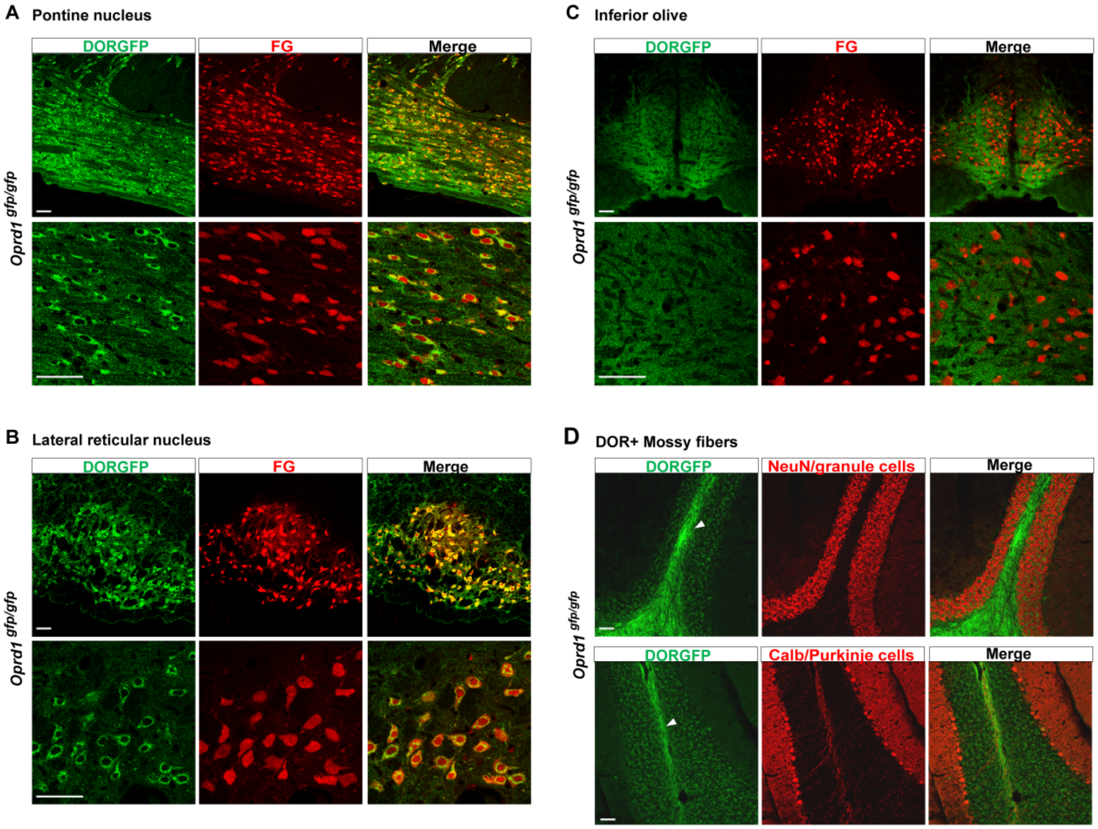
A
Pontine nucleus
DORGFP
FG
Merge
Oprd1 gfp/gfp
B
Lateral reticular nucleus
DORGFP
FG
Merge
Oprd1 gfp/gfp
C
Inferior olive
DORGFP
FG
Merge
Oprd1 gfp/gfp
D
DOR+ Mossy fibers
DORGFP
NeuN/granule cells
Merge
DORGFP
Calb/Purkinje cells
Merge
Oprd1 gfp/gfp

Figure S5

Figure S5. DOR Expression in Neurons that Project to the Cerebellum.

(Related to Figure 4)

(A) DOR is expressed by pontine nucleus neurons that project to the cerebellum, identified by fluorogold (FG) injection in cerebellum.

(B) Expression of DOR in lateral reticular nucleus neurons projecting to the cerebellum.

(C) DOR is absent in the inferior olive neurons that project to the cerebellum.

(D) DOR is present in mossy fibers (white arrowhead) innervating cerebellar granule cells, consistent with DOR expression in pre-cerebellar nuclei.

Scale bars represent 50 μM.

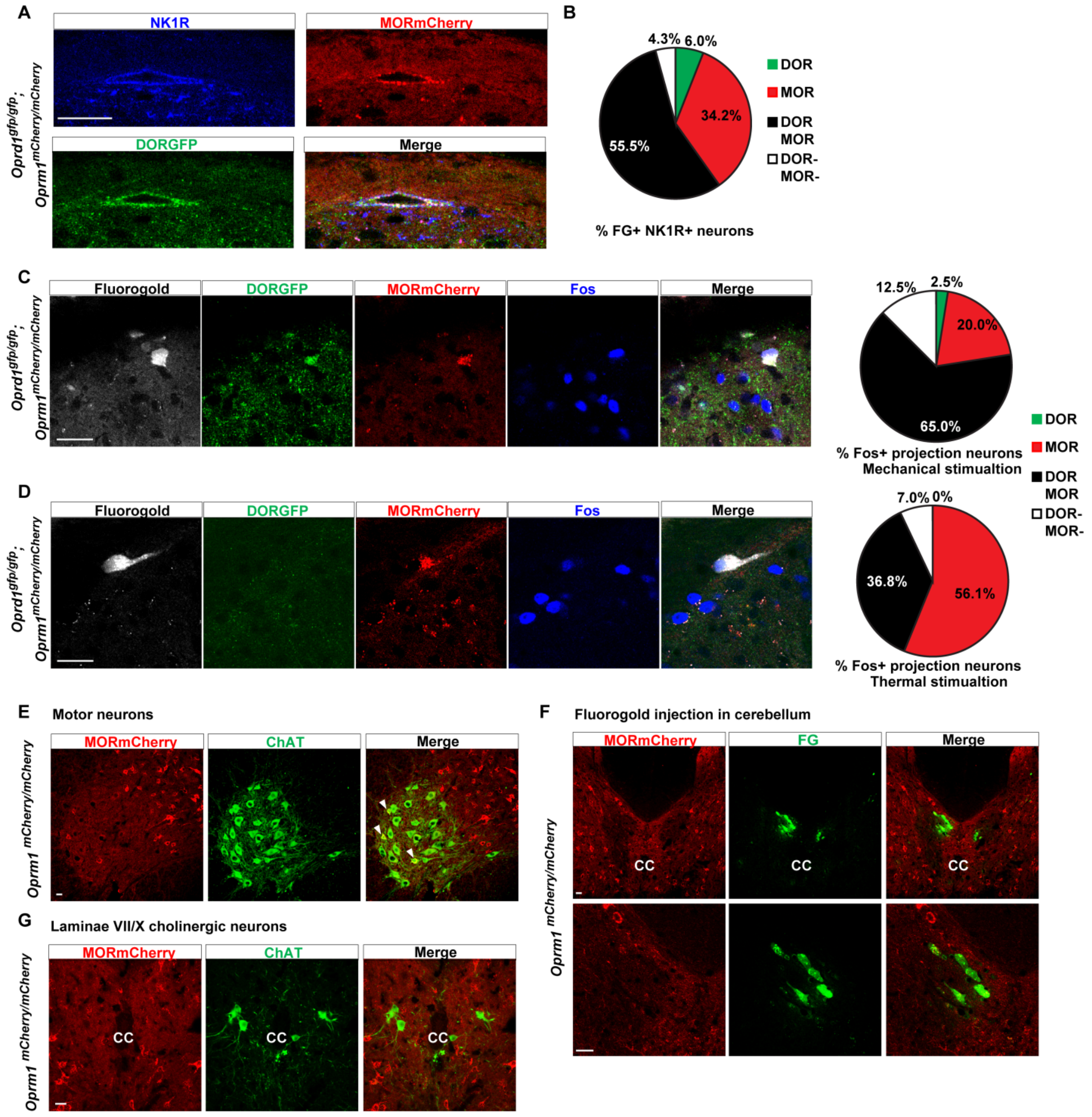
A
NK1R
MORmCherry
DORGFP
Merge
Oprd1gfp/gfp; Oprm1mCherry/mCherry
B
4.3%
6.0%
34.2%
55.5%
DOR
MOR
DOR MOR
DOR- MOR-
% FG+ NK1R+ neurons
C
Fluorogold
DORGFP
MORmCherry
Fos
Merge
12.5%
2.5%
20.0%
65.0%
% Fos+ projection neurons
Mechanical stimualtion
D
7.0%
0%
36.8%
56.1%
% Fos+ projection neurons
Thermal stimualtion
E
Motor neurons
ChAT
Oprm1 mCherry/mCherry
F
Fluorogold injection in cerebellum
FG
CC
G
Laminae VII/X cholinergic neurons

Figure S6

Figure S6. DOR and MOR are Co-expressed in Lamina I NK1R+ Projection Neurons but not in Motor Neurons.

(Related to Figure 6)

(A-B) DOR and MOR are co-expressed by 55.5% of lamina I NK1R+ projections (n=117 neurons from 3 mice).

(C) Fos is expressed by 65.0% of DOR+MOR+ projection neurons following mechanical stimulation of the hindpaw of DORGFP;MORmCherry mice.

(D) Noxious heat stimulation induced Fos expression in 92.8% of MORmCherry+ projection neurons.

(E) Double labeling of RFP and ChAT in spinal cord sections from MORmCherry mice shows that MOR is expressed by few ChAT+ motor neurons with small diameter cell bodies.

(F) MOR is largely absent from Clarke's column neurons. CC, central canal.

(G) MOR is rarely expressed by cholinergic partition cells and central canal cluster neurons.

Scale bars represent 20 μM.

A Lateral reticular nucleus

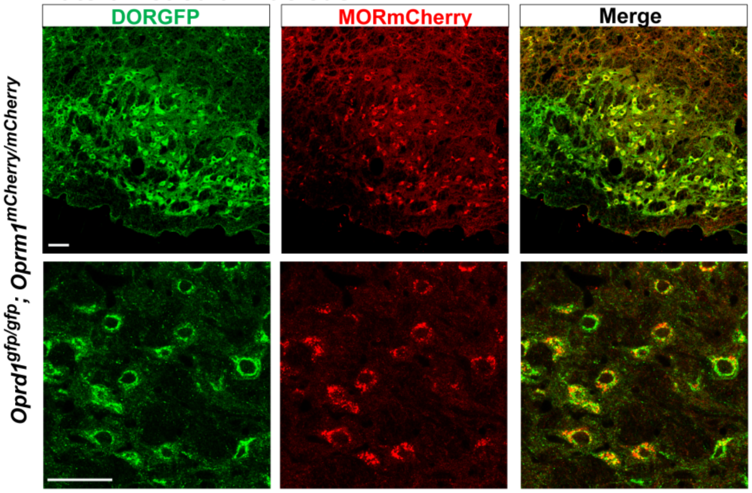

B Pontine nucleus

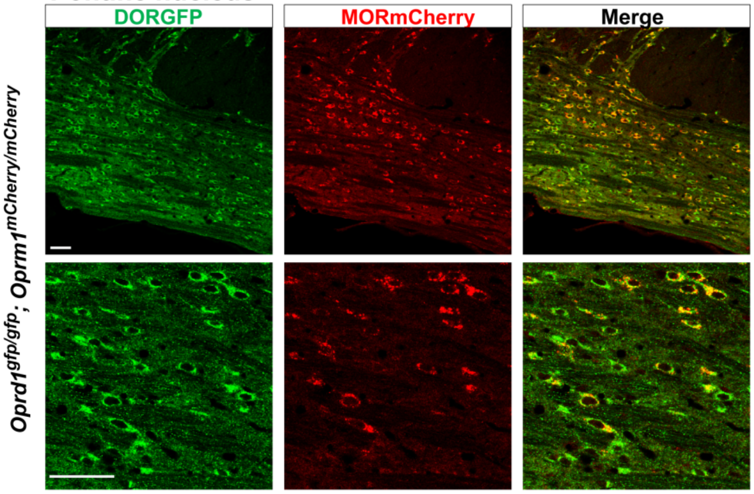

Figure S7

Figure S7. Co-expression of DOR and MOR in Pre-cerebellar Nuclei.

(Related to Figure 7)

(A) Co-staining with anti-GFP and -RFP antibodies in brain sections from DORGFP;MORmCherry mice indicate DOR and MOR are co-expressed by lateral reticular nucleus (LRt) neurons projecting to the cerebellum.

(B) Co-expression of DOR and MOR in pontine nucleus neurons projecting to the cerebellum.

Scale bars represent 50 μM.

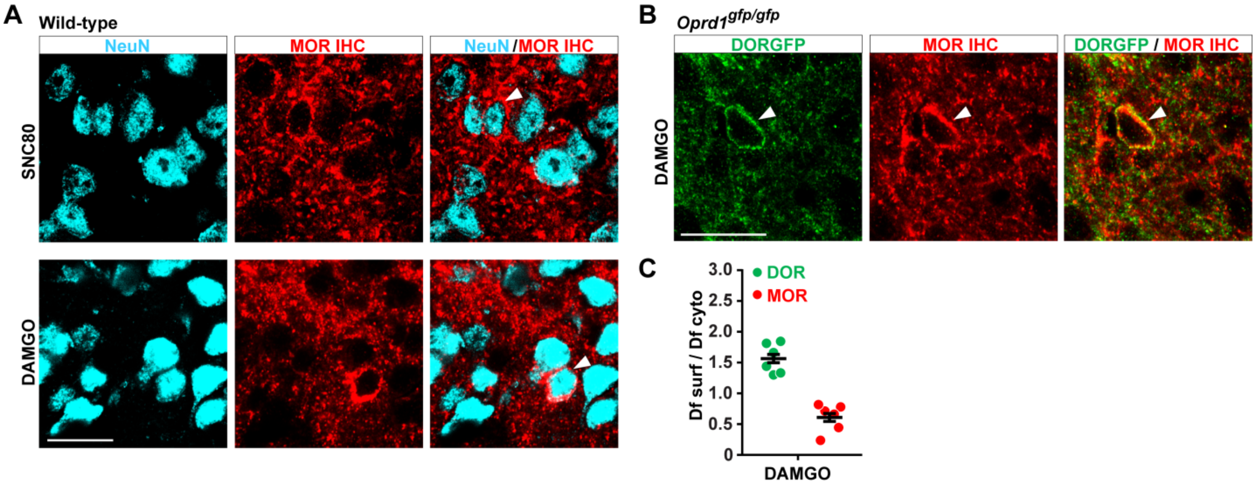
A
Wild-type
NeuN
MOR IHC
NeuN / MOR IHC
SNC80
DAMGO
B
Oprd1gfp/gfp
DORGFP
MOR IHC
DORGFP / MOR IHC
DAMGO
C
DOR
MOR
Df surf / Df cyto
3.0
2.5
2.0
1.5
1.0
0.5
0
DAMGO

Figure S8

Figure S8. The DOR Agonist SNC80 Does Not Cause MOR Co-internalization with DOR in Wild-type Mice.

(Related to Figure 8)

(A) Co-labeling of NeuN and MOR in sections from wild-type mice showing that MOR is internalized by its agonist DAMGO (100 ng, i.t.), but not co-trafficked to lysosomes with DOR following SNC80 treatment. White arrowheads show examples of MOR+ lamina II neurons.

(B) Co-staining with anti-GFP and anti-MOR antibodies in spinal cord sections from DORGFP mice shows that DAMGO causes MOR, without co-internalizing DOR. White arrowheads indicate DORGFP+ MOR+ neurons.

(C) Quantification of (B). Data are presented as mean ± SEM with dots showing individual neurons.

Scale bars represent 20 μM.

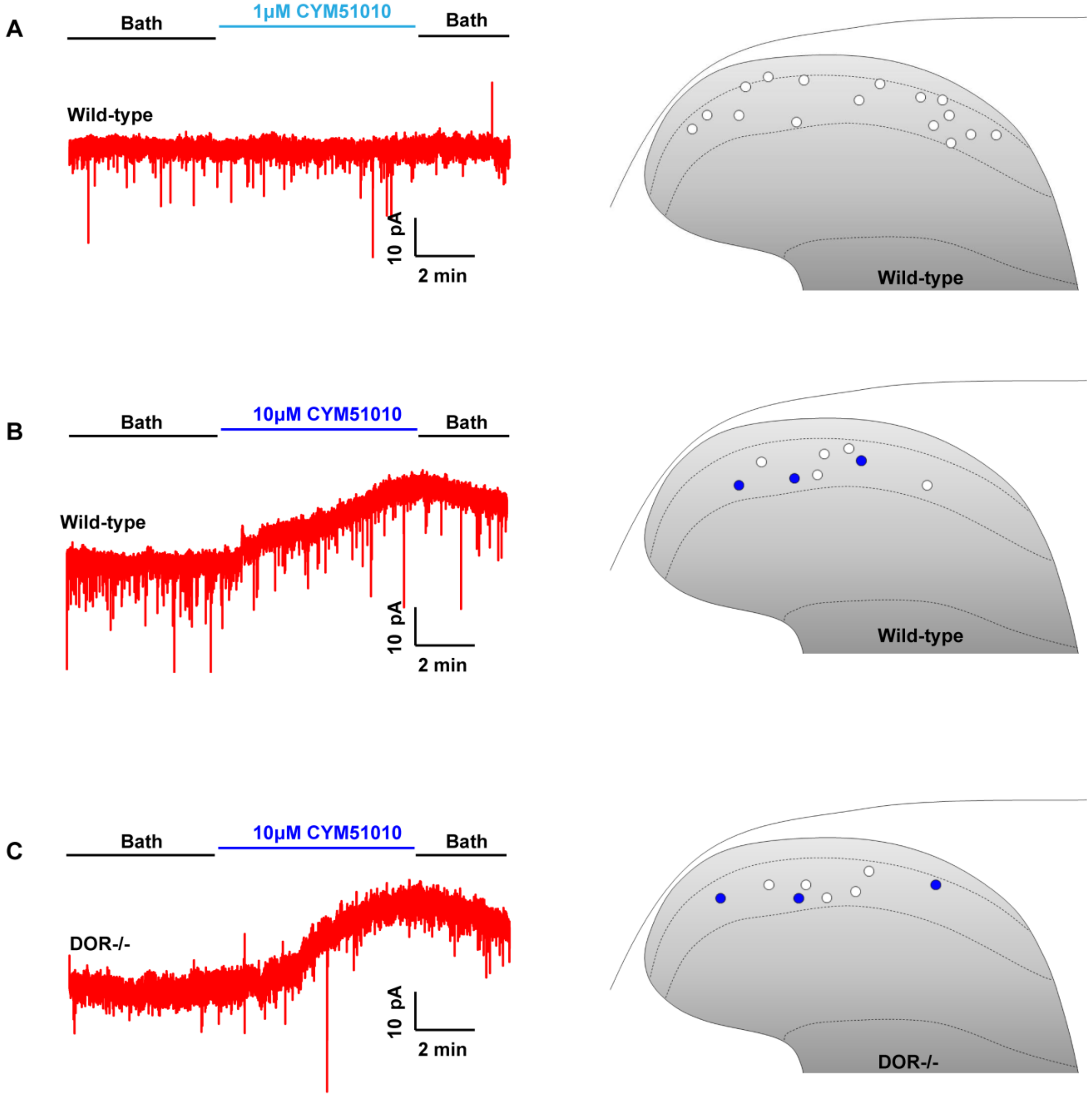
A
Bath
1μM CYM51010
Bath
Wild-type
10 pA
2 min
Wild-type
B
Bath
10μM CYM51010
Bath
Wild-type
10 pA
2 min
Wild-type
C
Bath
10μM CYM51010
Bath
DOR-/-
10 pA
2 min
DOR-/-

Figure S9

Figure S9. Effect of the Biased DOR-MOR Heteromer Agonist On GIRK Channel Currents in Wild-type and DOR KO Mice.

(Related to Figure 8)

(A) Representative trace and schematic map showing that at a dose of 1 μM, the biased DOR-MOR heteromer agonist CYM51010 does not activate GIRK channels in spinal cord dorsal horn neurons in slice from wild-type mice.

(B) 10 μM of CYM51010 did cause activation of GIRK channels in 3 out of 8 recorded dorsal horn neurons in slices from wild-type mice. Blue dots in the schematic map indicate CYM51010-responsive neurons.

(C) 10 μM of CYM51010 also activates GIRK channels in dorsal horn neurons from DOR KO mice, indicating that this response in not mediated by DOR-MOR heteromers, and suggesting that CYM51010 cannot be used to specifically study the neurophysiology of heteromers in slices.
