# Supplementary material for: Functional Divergence of Delta and Mu Opioid Receptor Organization in CNS Pain Circuits
Source: Neuron. 2018 Apr 4;98(1):90–108.e5. doi: 10.1016/j.neuron.2018.03.002 (PMC5896237; doi:10.1016/j.neuron.2018.03.002)
Supplement: Document S2. Article plus Supplemental Information [file mmc2.pdf]

Article

Neuron

Functional Divergence of Delta and Mu Opioid Receptor Organization in CNS Pain Circuits

Highlights

- DOR and MOR segregate in dorsal horn interneurons, and amygdalar and cortical neurons
- DOR and MOR are co-expressed in dorsal horn projection neurons and in ventral horn
- MOR is not co-degraded with DOR in neurons that co-express both receptors *in vivo*
- DOR in SOM+ dorsal horn interneurons controls mechanical pain and but not heat pain

Authors

Dong Wang, Vivianne L. Tawfik, Gregory Corder, Sarah A. Low, Amaury François, Allan I. Basbaum, Grégory Scherrer

Correspondence

gs25@stanford.edu

In Brief

Wang et al. show that the delta and mu opioid receptors are present in mostly different populations of pain neurons in the spinal cord and brain, indicating that they may control distinct types of pain or distinct aspects of pain experience.

Wang et al., 2018, Neuron *98*, 90–108
April 4, 2018 © 2018 The Authors. Published by Elsevier Inc.
https://doi.org/10.1016/j.neuron.2018.03.002

Neuron
Article

Functional Divergence of Delta and Mu Opioid Receptor Organization in CNS Pain Circuits

Dong Wang,[1,2,3,4] Vivianne L. Tawfik,[1,2,3,4] Gregory Corder,[1,2,3,4] Sarah A. Low,[1,2,3,4] Amaury François,[1,2,3,4] Allan I. Basbaum,[5] and Grégory Scherrer[1,2,3,4,6,7,*]
[1]Department of Anesthesiology, Perioperative and Pain Medicine, Stanford University, Palo Alto, CA 94304, USA
[2]Department of Molecular and Cellular Physiology, Stanford University, Palo Alto, CA 94304, USA
[3]Department of Neurosurgery, Stanford University, Palo Alto, CA 94304, USA
[4]Stanford Neurosciences Institute, Stanford University, Palo Alto, CA 94304, USA
[5]Department of Anatomy, University of California, San Francisco, San Francisco, CA 94158, USA
[6]New York Stem Cell Foundation – Robertson Investigator, Stanford University, Palo Alto, CA 94304, USA
[7]Lead Contact
*Correspondence: gs25@stanford.edu
https://doi.org/10.1016/j.neuron.2018.03.002

SUMMARY

Cellular interactions between delta and mu opioid receptors (DORs and MORs), including heteromerization, are thought to regulate opioid analgesia. However, the identity of the nociceptive neurons in which such interactions could occur *in vivo* remains elusive. Here we show that DOR-MOR co-expression is limited to small populations of excitatory interneurons and projection neurons in the spinal cord dorsal horn and unexpectedly predominates in ventral horn motor circuits. Similarly, DOR-MOR co-expression is rare in parabrachial, amygdalar, and cortical brain regions processing nociceptive information. We further demonstrate that in the discrete DOR-MOR co-expressing nociceptive neurons, the two receptors internalize and function independently. Finally, conditional knockout experiments revealed that DORs selectively regulate mechanical pain by controlling the excitability of somatostatin-positive dorsal horn interneurons. Collectively, our results illuminate the functional organization of DORs and MORs in CNS pain circuits and reappraise the importance of DOR-MOR cellular interactions for developing novel opioid analgesics.

INTRODUCTION

Opioids, such as morphine, are the mainstay for the treatment of moderate to severe pain (Fields, 2004). Classic radioligand binding studies have established that opioid receptors are present in the dorsal horn of the spinal cord (Snyder and Pasternak, 2003), which is a major center for the processing of pain information, and its modulation by opioid analgesics (Basbaum et al., 2009; Braz et al., 2014; Todd, 2010). However, the precise mechanisms, and in particular the neural circuits, cell, and receptor populations underlying opioid analgesia, remain poorly defined.

Cellular interactions between the delta and mu opioid receptors (DORs and MORs, respectively) are thought to regulate pain and opioid analgesic efficacy (Fujita et al., 2015; Ong and Cahill, 2014; van Rijn et al., 2010). Co-immunoprecipitation experiments, including those using spinal cord tissue, indicated that DOR and MOR directly interact and may form heteromers (Fan et al., 2005; Gomes et al., 2004). Furthermore, activation of DORs by DOR agonists has been proposed to induce co-internalization and degradation of MORs, thereby reducing the analgesic efficacy of MOR agonists acutely, and contributing to morphine tolerance (He et al., 2011). These results supported the idea that interfering with DOR function, by gene knockout, with DOR antagonists or with bivalent ligands with MOR agonist and DOR antagonist properties, might potentiate morphine analgesia and reduce morphine tolerance (Abdelhamid et al., 1991; Chefer and Shippenberg, 2009; Daniels et al., 2005; Fujita et al., 2015; Gomes et al., 2004; Schiller, 2005; Zhu et al., 1999). However, a difficulty in interpreting these data result from the fact that the DOR and MOR share approximately 60% amino acid sequence identity in mice, such that ligands that are reported to bind selectively to MOR or DOR can, at high doses, bind to both receptors. Consequently, direct cross binding of DOR ligands to MOR, rather than functional interaction between these receptors, might explain some of the previous results. For example, even limited occupancy of MOR by a DOR antagonist could reduce morphine efficacy. In fact, studies of DOR knockout mice have led to conflicting conclusions, showing either no change (Scherrer et al., 2009) or reduced morphine tolerance (Zhu et al., 1999).

Biochemical approaches used previously to study DOR-MOR interactions could not identify the neural circuits and cells in which these interactions might occur. Given the considerable diversity in cell populations within somatosensory and motor control spinal circuits, this gap in knowledge precludes understanding how spinal DORs, MORs, and possible heteromers, control pain, and what somatosensory modalities (e.g., heat versus mechanical pain, acute versus chronic pain, but also itch, touch, and proprioception) are sensitive to opioid ligands. Immunohistochemical studies indicated that MOR is expressed by peptidergic nociceptors, and by excitatory spinal neurons, including lamina I projection

 © 2018 The Authors. Published by Elsevier Inc.
This is an open access article under the CC BY license (http://creativecommons.org/licenses/by/4.0/).

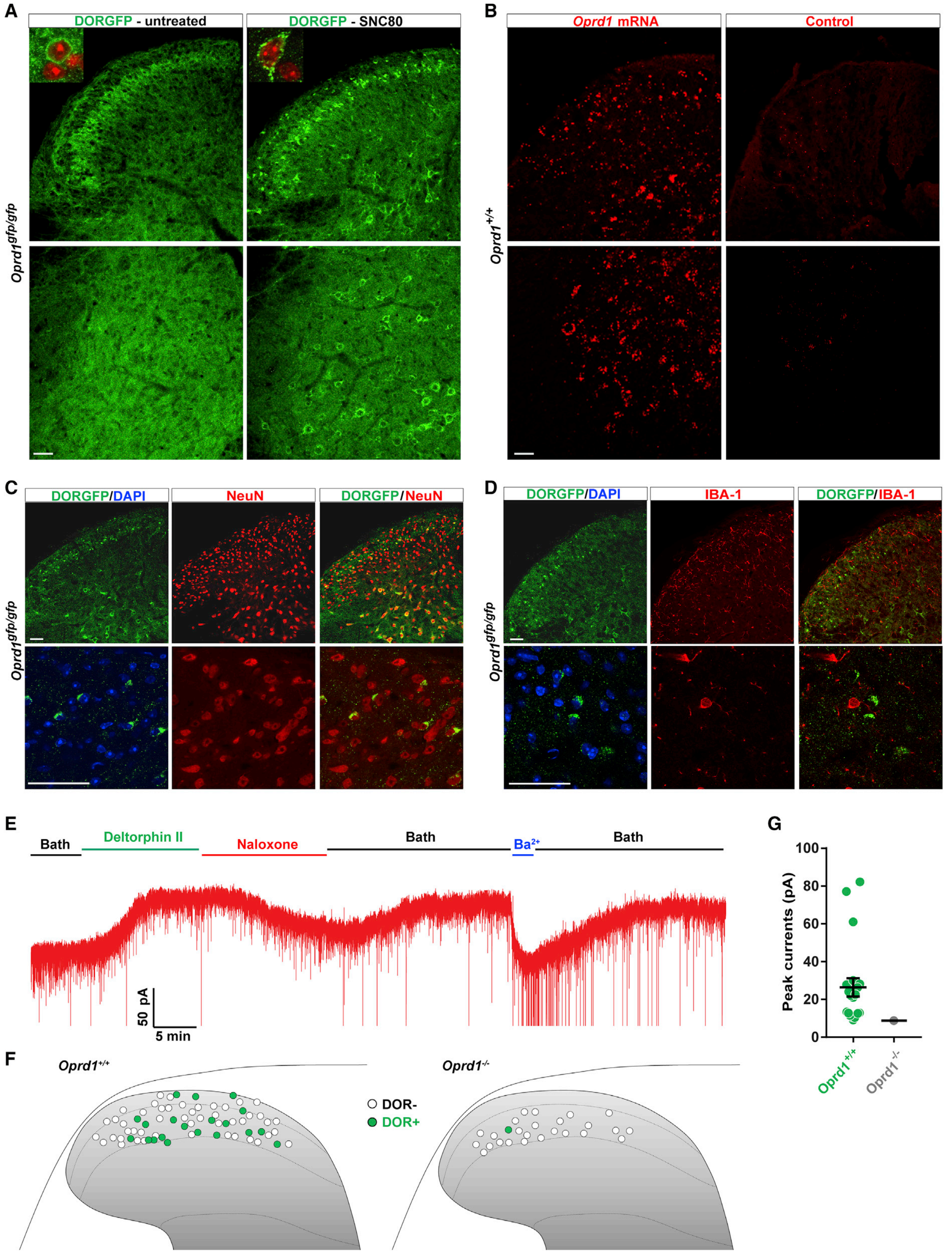

(legend on next page)

neurons, and lamina II excitatory interneurons (Arvidsson et al., 1995; Bardoni et al., 2014; Kemp et al., 1996; Li et al., 1998; Scherrer et al., 2009; Spike et al., 2002). In contrast, the DOR expression pattern continues to be a subject of intense debate. Studies relying on an antibody (Ab$^{3-17}$) (Dado et al., 1993) suggested that DOR is also expressed by peptidergic nociceptors, but not by spinal neurons. This conclusion followed from the observation that Ab$^{3-17}$-immunoreactivity (ir) co-localizes with substance P and CGRP in DRG neuron cell bodies and terminals in the dorsal horn (Bao et al., 2003; Dado et al., 1993; Guan et al., 2005; Overland et al., 2009; Wang et al., 2010) and is lost in the dorsal horn following deafferentation (Dado et al., 1993). Based on these findings, DOR-MOR heteromers and cellular interactions between these receptors in the spinal cord were thought to occur in the central terminals of peptidergic nociceptors.

In contrast, we previously showed, using a DORGFP reporter mouse, as well as *in situ* hybridization and electrophysiology in wild-type mice, that DOR and MOR are expressed in largely distinct populations of DRG neurons (Bardoni et al., 2014; François and Scherrer, 2017; Scherrer et al., 2009). Although MOR is indeed enriched in unmyelinated peptidergic nociceptors, DOR predominates in myelinated mechanoreceptors and unmyelinated non-peptidergic nociceptors. Recent expression studies using highly sensitive single-cell RNA sequencing confirmed the segregated expression of DOR and MOR in DRG neurons (Usoskin et al., 2015). Since we observed that Ab$^{3-17}$-ir pattern persists in two strains of *Oprd1* knockout (DOR KO) mice (Scherrer et al., 2009) and does not match the distribution patterns of *Oprd1* mRNA or DOR radioligand binding, we concluded that Ab$^{3-17}$-ir might not accurately represent DOR expression in DRG and CNS. Therefore, the identity of the spinal cord neurons that express DOR, and the extent to which there is MOR co-expression and potential heteromerization in these cells, remains to be determined.

Here we provide a comprehensive histological, electrophysiological, and behavioral analysis that establishes the principles of opioid receptor functional organization in CNS circuits that transmit and process pain signals.

RESULTS

DORGFP Internalization Reveals the Distribution of DOR+ Spinal Neurons

We first used DORGFP reporter mice (Scherrer et al., 2006) and GFP immunolabeling to determine the DOR expression pattern in the spinal cord. Consistent with the binding pattern of DOR radioligands (Bardoni et al., 2014; Mennicken et al., 2003; Scherrer et al., 2009), we observed diffuse DORGFP expression throughout the spinal cord gray matter, with a relatively brighter DORGFP+ band in lamina II (Figure 1A, left). To identify DORGFP+ cell bodies, we took advantage of the trafficking properties of DOR, wherein binding of agonists results in internalization and accumulation of the receptor in perinuclear lysosomes for degradation (Pradhan et al., 2009; Scherrer et al., 2006; Tsao and von Zastrow, 2000; Wang et al., 2003; Whistler et al., 2002).

Remarkably, pre-treating DORGFP mice with the DOR agonist SNC80 uncovered the distribution of a very large number of DOR+ cell bodies, both in the dorsal and ventral horns (Figure 1A, right). DORGFP+ spinal cells co-express the pan neuronal marker NeuN (Figure 1C), but not the microglial markers IBA-1 (Figure 1D), P2Y12, or CD11b (Figures S1A and S1B), indicating that they are neurons. Labeling of the central terminals of CGRP+ and IB4+ nociceptors, and of PKCγ interneurons, indicated that DORGFP+ neurons are particularly enriched at the ventral border of lamina II inner (lamina IIiv) (Figures S1C and S1D). We next used *in situ* hybridization and electrophysiology in wild-type mice to further test the hypothesis that DOR is expressed by spinal neurons. Consistent with the DORGFP expression pattern, *Oprd1* mRNA is present in numerous neurons throughout the spinal cord gray matter of wild-type mice, mainly in small lamina II neurons, and in larger neurons in the ventral horn (Figure 1B).

In CNS neurons, postsynaptic opioid receptors are generally coupled to G protein-coupled inwardly rectifying potassium (GIRK) channels. In spinal cord slices of wild-type mice, we bath perfused the DOR agonist deltorphin II and recorded GIRK channel-mediated increases in holding currents in randomly selected neurons, focusing on lamina II. We found that deltorphin II induced an outward current in 29.4% (20/68) of recorded neurons (Figures 1E–1G). Deltorphin II-responsive neurons were concentrated in lamina II inner, in agreement with the distribution of both DORGFP and *Oprd1* mRNA. Naloxone, an opioid receptor antagonist, or barium (Ba^{2+}), a potassium channel blocker, blocked the deltorphin II-induced currents (Figure 1E). To confirm deltorphin II selectivity for DOR, we performed identical recordings in spinal cord slices from DOR KO mice. In only one out of 26 recorded neurons did we observe a small deltorphin II-induced GIRK current (8.8 pA), possibly due to deltorphin II-mediated activation of other opioid receptors in the absence of DOR (Figures 1F and 1G). Based on these results,

Figure 1. Receptor Trafficking in DORGFP Mouse Reveals DOR+ Neurons in Spinal Cord

(A) Staining with an anti-GFP antibody in spinal cord sections from either untreated or SNC80-pretreated (10 mg/kg, s.c., 2 hr before tissue collection) DORGFP knockin mice.

(B) *Oprd1* mRNA in spinal cord sections from wild-type mice.

(C) Co-localization of DORGFP with the neuronal marker NeuN.

(D) DORGFP+ cells do not express the microglia marker IBA-1.

(E) Deltorphin II activates GIRK channels in spinal cord dorsal horn neurons in wild-type mice.

(F) Schematic map showing the location of all recorded dorsal horn neurons in wild-type (n = 68) or DOR knockout mice (n = 26). Green recorded neurons presented deltorphin II-induced GIRK currents.

(G) Quantification of peak GIRK channel currents from deltorphin II-responsive neurons in (F). Data are presented as mean ± SEM with dots showing individual neurons.

Scale bars represent 50 μM. See also Figure S1.

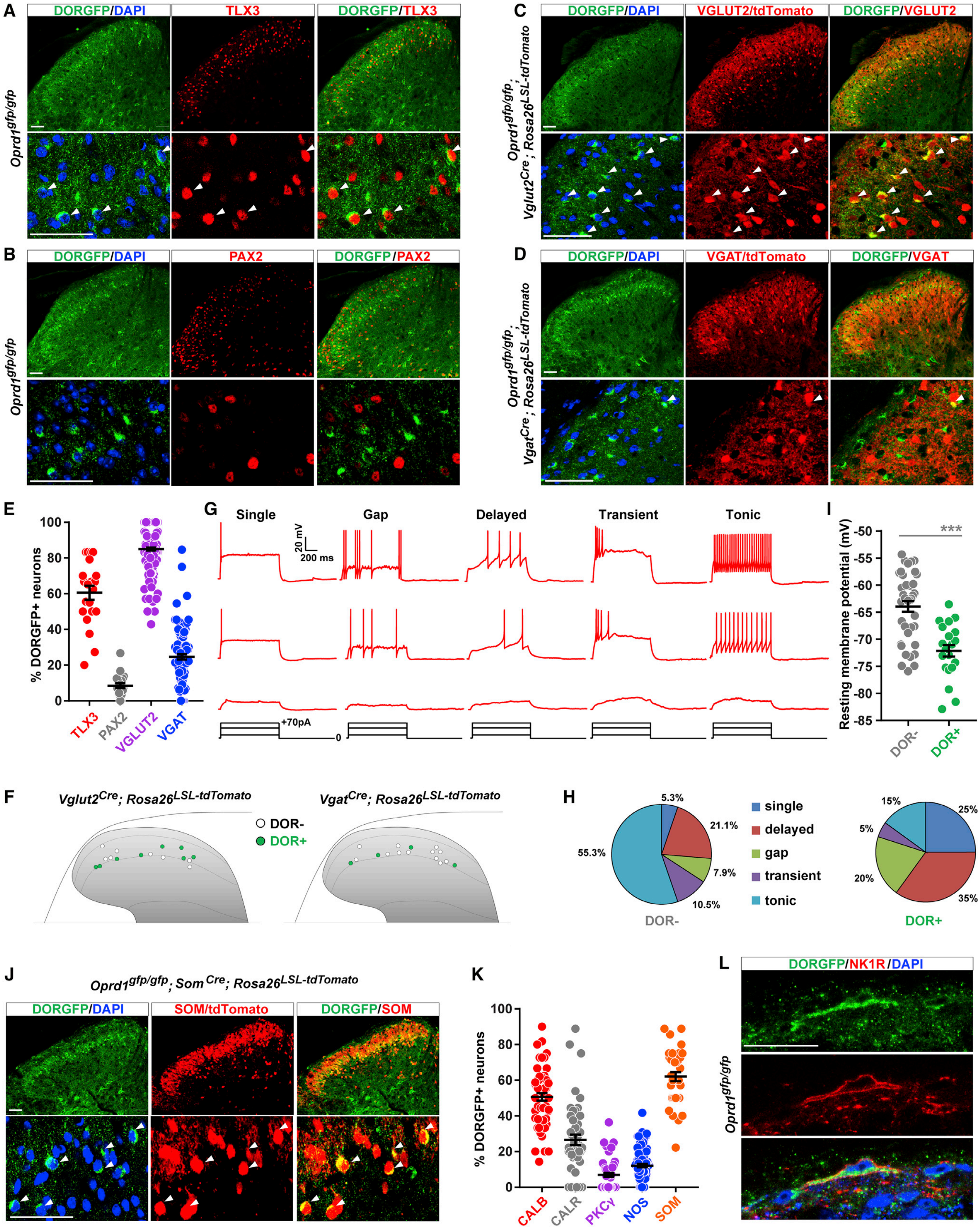

(legend on next page)

we conclude that DOR is not only expressed by primary afferent DRG neurons, but also by numerous spinal neurons, both in the dorsal and ventral horns.

Dorsal Horn SOM+ Lamina II Excitatory Interneurons Express DOR

We next characterized the different populations of DOR+ spinal neurons. Superficial dorsal horn neurons, which originate from the Lbx1+ neuronal lineage, can be divided into TLX3+ excitatory and PAX2+ inhibitory neurons (Alaynick et al., 2011). Co-immunostaining revealed that 60.5% of the DORGFP+ neurons located in lamina II express TLX3, but that only 8.5% express PAX2 (Figures 2A, 2B, and 2E). Furthermore, we crossed DORGFP mice with *Vglut2*^{Cre}*;Rosa26*$^{LSL\text{-}tdTomato}$ and *Vgat*^{Cre}*;Rosa26*$^{LSL\text{-}tdTomato}$ reporter mice and found that 85.0% of DORGFP+ lamina II neurons co-express the vesicular glutamate transporter 2 (VGLUT2), while only 24.6% co-express the vesicular GABA transporter (VGAT) (Figures 2C–2E). Consistent with these anatomical results, whole-cell recordings from VGLUT2/tdTomato+ or VGAT/tdTomato+ lamina II neurons in mice with a wild-type *Oprd1* allele showed that 50.0% (8/16) of VGLUT2/tdTomato+ and 25.0% (4/16) of VGAT/tdTomato+ neurons displayed deltorphin II-induced GIRK currents, respectively (Figure 2F). For lamina II interneurons, neurotransmitter phenotype is thought to be correlated with action potential (AP) firing patterns; inhibitory neurons most commonly display a tonic AP firing pattern, while excitatory neurons generally show delayed, gap, or single AP firing patterns (Abraira et al., 2017; Yasaka et al., 2010). Excitatory interneurons also display more negative resting membrane potentials, compared to inhibitory interneurons (Yasaka et al., 2010). We found that 80.0% (16/20) of DOR+ neurons show a delayed, gap, or single firing pattern (Figures 2G and 2H) and that DOR+ neurons displayed a more negative resting membrane potential (−72 mV), compared to DOR-negative neurons (−64 mV) (Figure 2I). Together, these results indicate that the great majority of DOR+ neurons in the dorsal horn are lamina II excitatory interneurons.

We further resolved the molecular identity of lamina II DORGFP+ excitatory neurons using antibodies or reporter mouse lines that distinguish dorsal horn interneuron subpopulations, including calbindin (CALB), calretinin (CALR), nitric oxide synthase (NOS), protein kinase C gamma (PKCγ), and somatostatin (SOM) (Figures 2J, 2K, and S2A–S2D). These experiments demonstrated that, among these markers, SOM is most often co-expressed by DORGFP+ lamina II neurons (62.0%). Although SOM+ neurons are distributed throughout lamina II, we found that the great majority of DOR+ SOM+ neurons are located in lamina IIiv, at the ventral border between laminae II-III (Figure 2J), intermixed with PKCγ+ interneurons, which only rarely express DOR (Figures 2K and S2D). The rare DORGFP+ lamina IIiv neurons that co-express NOS likely correspond to a small population of SOM-negative inhibitory interneurons (Figures 2K and S2C). DOR+ excitatory interneurons also frequently co-express CALB, and to a lesser extent CALR, particularly when they are located more dorsally in lamina II (Figures 2K, S2A, and S2B).

To complete the characterization of DOR+ neurons in the dorsal horn, we also identified the few DOR+ neurons present in lamina I, in laminae III-V, and in the lateral spinal nucleus (LSN). Co-expression of DOR with neurokinin 1 receptor (NK1R), the receptor for substance P, in large lamina I and LSN neurons suggests that DOR is present in a subpopulation of glutamatergic projection neurons that relay pain information to the brain (Figure 2L). Finally, we determined that DOR is absent from lamina III, which contains parvalbumin+ (PARV) inhibitory neurons that gate mechanical hypersensitivity (Petitjean et al., 2015) but frequently is co-expressed with PARV in deeper dorsal horn neurons (laminae IV-V) (Figure S2E).

DOR Agonists Inhibit Activity of SOM+ Interneurons and Decrease Mechanical Pain

A recent cell ablation study demonstrated that lamina II SOM+ interneurons are critical for acute mechanical pain and injury-induced mechanical hypersensitivity (Duan et al., 2014). Given our finding that DOR is co-expressed with SOM in lamina II interneurons, we hypothesized that DOR+ lamina II neurons might also be part of mechanical pain circuits. To test this hypothesis, we first stimulated the hindpaw of DORGFP mice with a noxious mechanical stimulus and used Fos immunostaining to identify mechano-nociceptive dorsal horn neurons. Figures 3A and 3B show that 40.0% of DORGFP+ lamina II neurons were Fos+, among which 22.3% were also SOM+ (Figures S3A and S3B), indicating that DOR is indeed expressed by dorsal horn neurons that process cutaneous mechano-nociceptive information.

Aδ and Aβ fibers, including myelinated mechanonociceptors (AMs) and low-threshold mechanoreceptors (A-LTMRs) are essential contributors to cutaneous mechanosensation. Using

Figure 2. Most DOR-Expressing Neurons in the Dorsal Horn are Lamina II Excitatory Interneurons

(A) DORGFP+ neurons frequently co-express TLX3 (white arrowheads).
(B) DORGFP+ neurons rarely express PAX2.
(C) Most DORGFP+ neurons express VGLUT2/tdTomato (white arrowheads).
(D) Few DORGFP+ neurons express VGAT/tdTomato (white arrowheads).
(E) Quantification of (A)–(D). Dots represent individual counts in spinal cord sections from 3–6 mice.
(F) Schematic map of the location of VGLUT2+ or VGAT+ neurons that responded to deltorphin II (i.e., DOR+, green). n = 16 neurons for each.
(G) Whole-cell recording indicated different AP firing patterns in deltorphin II-responsive neurons in wild-type mice.
(H) Proportions of DOR+ or DOR- neurons showing the different AP firing patterns.
(I) DOR+ neurons have a more negative resting membrane potential compared to DOR− neurons. ***$p < 0.001$ with unpaired t test.
(J) DORGFP+ lamina II neurons frequently co-express somatostatin (white arrowheads).
(K) Quantification of DORGFP+ neuron co-expression with multiple other neuronal markers. Dots represent individual counts in spinal cord sections from 3–6 mice.
(L) DOR is expressed by a subpopulation of lamina I NK1R+ projection neurons.
Data are presented as mean ± SEM in (E), (I), and (K). Scale bars represent 50 μM. See also Figure S2.

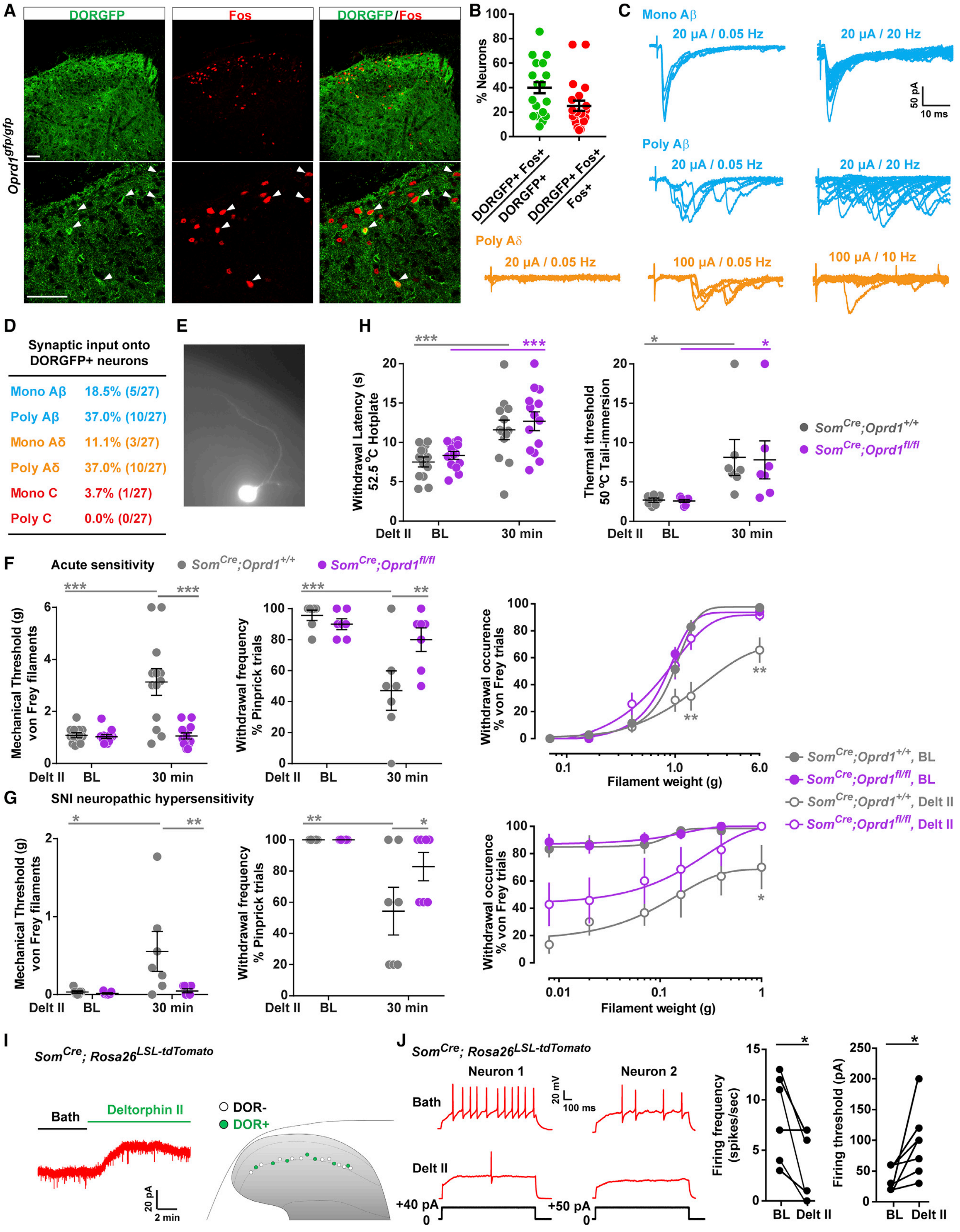

(legend on next page)

spinal cord slices with dorsal root attached, we found that the majority of DORGFP+ lamina II neurons receive mono- and poly-synaptic Aδ and Aβ inputs, but rarely C fiber inputs (Figures 3C and 3D). Additionally, these recorded lamina II DORGFP+ neurons extend a process dorsally, toward lamina I (Figure 3E), suggesting that these cells may relay mechanosensory myelinated afferent input to lamina I projection neurons.

DOR agonists are particularly effective at reducing pain provoked by mechanical stimuli (Cahill et al., 2001; Pradhan et al., 2009; Scherrer et al., 2009); however, the mechanisms underlying these properties remain unclear. DOR agonist antinociceptive effects are observed following not only systemic but also intrathecal delivery of the drug and were thought to result from an action on DORs present on primary afferent central terminals. Our finding that DOR is expressed by mechano-nociceptive dorsal horn neurons suggested that DOR agonists might also act centrally on DORs in SOM+ lamina II neurons, to reduce mechanical pain. To test this possibility, we deleted DOR selectively from SOM+ neurons, by crossing mice bearing conditional (i.e., floxed) *Oprd1* alleles (*Oprd1*$^{lox/lox}$ mouse) (Gaveriaux-Ruff et al., 2011) with mice in which Cre recombinase expression is driven by the somatostatin gene (*Som*^{Cre}). We then evaluated the ability of intrathecal deltorphin II to decrease sensitivity to mechanical stimulation in the DOR conditional knockout mice (DOR cKO), by stimulating the mouse hindpaw with calibrated von Frey hairs or pinprick, and recording nociceptive withdrawal responses. We found that the deltorphin II-induced decrease in mechanical sensitivity observed in control littermates is lost in DOR cKO mice (Figure 3F). Similarly, in models of neuropathic and inflammatory mechanical hypersensitivity, deltorphin II anti-allodynic effect was profoundly reduced following deletion of DOR in SOM+ neurons (Figures 3G and S3G). As we did not observe expression of DOR in the SOM+ DRG neurons (Figures S3C and S3D), and very limited *Som* expression in ventral horn (Figures S3E and S3F), the effect is dorsal horn SOM+ neuron specific. This result suggests that intrathecal DOR agonists act, at least in part, on DOR expressed by SOM neurons to diminish mechanical sensitivity and that action of the drug exclusively on presynaptic DORs expressed by DRG neurons is not sufficient to cause a significant antinociceptive effect. Finally, we also measured the effect of deltorphin II on heat sensitivity and found that the antinociceptive action of deltorphin II in the hotplate and tail immersion tests was intact in DOR cKO mice (Figure 3H).

To test the possibility that deltorphin II could decrease mechanical sensitivity by influencing SOM+ interneuron excitability, we next recorded from tdTomato+ lamina II neurons in spinal cord slices from *Som*^{Cre};*Rosa26*$^{LSL\text{-}tdTomato}$ mice. Figure 3I shows that bath application of deltorphin II caused an increase in holding current in about half of the tdTomato+ recorded neurons. Furthermore, in the neurons in which we observed this hyperpolarization (i.e., deltorphin II responsive and thus DOR+), deltorphin II significantly decreased action potential firing (Figure 3J). Interestingly, DORs in SOM+ neurons might also be targeted by the endogenous peptide enkephalins. Using *Penk*^{Cre} mice (Francois et al., 2017), and anterograde tracing with wheat germ agglutinin (WGA), we not only found that approximately 52% of DOR+ neurons are Penk+ and presumably enkephalinergic, suggesting autosignaling mechanisms, but also that Penk+ neurons are occasionally presynaptic to DOR-expressing neurons (Figures S4A and S4C–S4E).

DOR Is Expressed by Several Classes of Neurons that Regulate Motor Control

We next identified the ventral horn neurons that express DOR. Two observations suggested that DOR is expressed by V1 inhibitory interneurons, which include pre-motor Ia interneurons mediating inhibition of antagonist muscles, and Renshaw cells mediating motor neuron recurrent inhibition. First, we observed that in spinal cord sections from *Oprd1*$^{gfp/gfp}$;*Vgat*^{Cre};*Rosa26*$^{LSL\text{-}tdTomato}$ mice the great majority of DORGFP+ ventral horn neurons were also tdTomato+, i.e., likely GABA/glycinergic interneurons (Figure 4A). We confirmed this observation in another reporter mouse line, *Vgat*^{Cre};*Rosa26*$^{LSL\text{-}ZsGreen}$ mice (Figure 4B). Second, these DORGFP+ ventral horn neurons included CALB+ neurons positioned deep, close to the white matter, identifying them as Renshaw cells (Figure 4C). To establish more definitively that DOR is expressed by V1 interneurons, we crossed DORGFP reporter mice with mice in which tdTomato expression is driven by the *En1* gene, a marker of V1 interneurons (*Oprd1*$^{gfp/gfp}$;*En1*^{Cre};*Rosa26*$^{LSL\text{-}tdTomato}$ mice). Figure 4D shows that the majority of V1 interneurons indeed express DOR, including CALB+ Renshaw cells.

Figure 3. DOR Agonist Inhibits SOM+ Interneurons to Decrease Mechanical Pain

(A) Noxious mechanical stimulation of the hindpaw of DORGFP mice induced Fos expression in DORGFP+ dorsal horn neurons (white arrowheads).

(B) Quantification of (A). Dots represent individual counts in spinal cord sections from 3 mice.

(C) Representative traces showing Aβ and Aδ fiber input to DORGFP+ neurons.

(D) Summary of (C). n = 27 neurons.

(E) Morphology of a DOR+ neuron during recording.

(F) Decreased effect of intrathecal deltorphin II (1 μg) against acute mechanical nociception in DOR cKO mice. Von Frey threshold test: n = 13 control mice and 14 DOR cKO mice; pinprick and von Frey frequency tests: n = 7 control mice and 7 DOR cKO mice.

(G) Diminished anti-allodynic effect of intrathecal deltorphin II (15 μg) against SNI-induced neuropathic hypersensitivity in DOR cKO mice. Von Frey threshold and pinprick tests: n = 7 control mice and 7 DOR cKO mice; von Frey frequency test: n = 6 control mice and 7 DOR cKO mice.

(H) The antinociceptive action of intrathecal deltorphin II (1 μg) against heat nociception (hotplate and tail immersion tests) is intact in DOR cKO mice. Hotplate test: n = 13 control mice and 14 DOR cKO mice); tail immersion test: n = 7 control mice and 7 DOR cKO mice.

(I) Representative trace showing deltorphin II-induced GIRK currents (labeled in green) in tdTomato+ lamina II neuron in slices from *Som*^{Cre};*Rosa26*$^{LSL\text{-}tdTomato}$ mice.

(J) Deltorphin II reduces the AP firing rate and increases the AP firing threshold in SOM/tdTomato+ neurons (n = 7).

*p < 0.05, **p < 0.01, ***p < 0.001, (F)–(H), repeated-measures, two-way ANOVA + Bonferroni; (J), paired t test.

Data are presented as mean ± SEM in (B) and (F)–(H). Scale bars represent 50 μM. See also Figures S3 and S4.

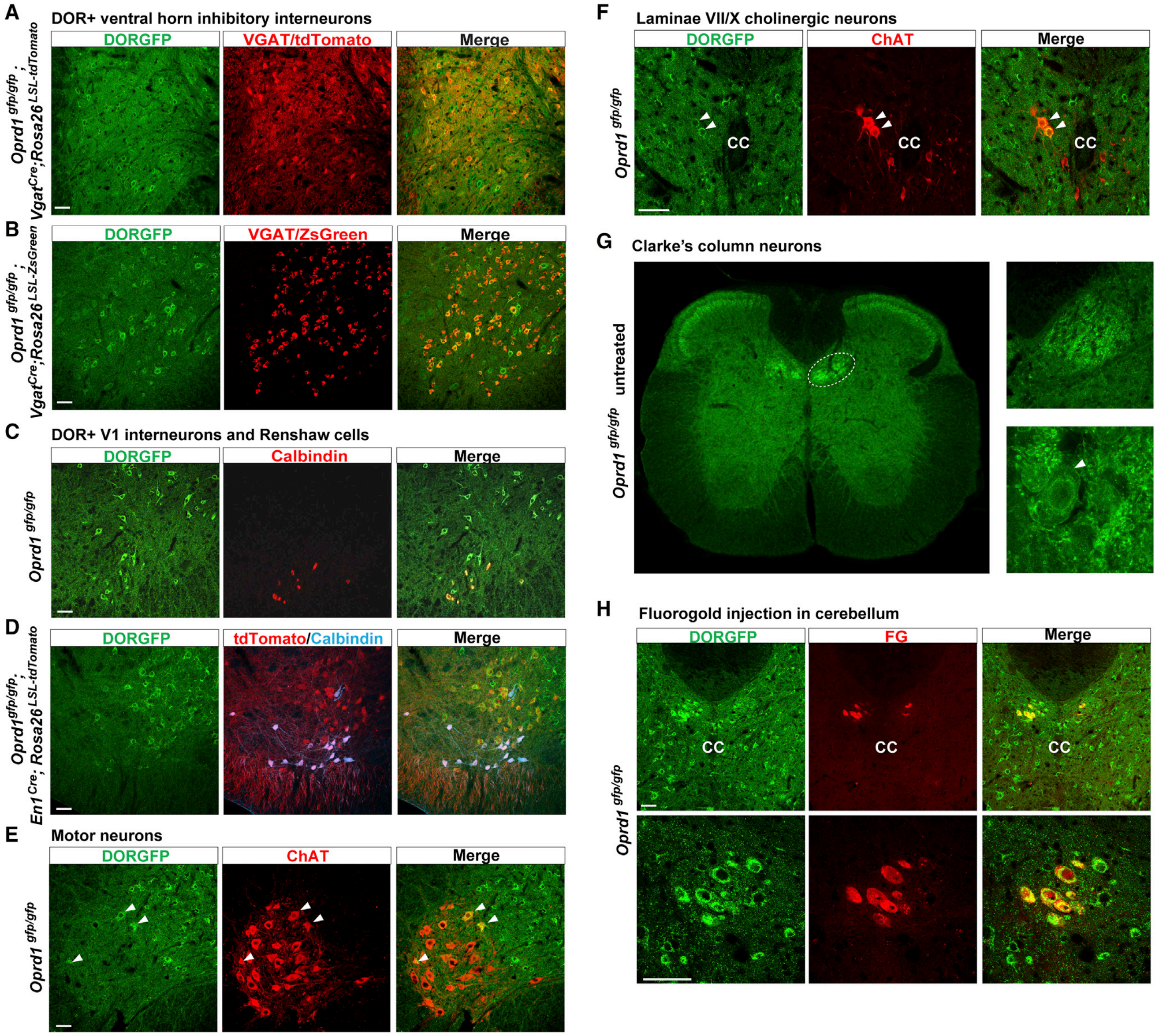

Figure 4. DOR Is Expressed by Several Classes of Spinal Neurons Regulating Motor Control

(A and B) Staining of sections from VGAT-tdTomato (A) or VGAT-Zsgreen (B) reporter mice crossed with DORGFP mice with an anti-GFP antibody indicates that ventral horn DOR+ neurons are inhibitory interneurons.

(C and D) DOR is expressed by En1+ V1 inhibitory neurons (D) including calbindin+ Renshaw cells (C).

(E) DOR is rarely expressed by motor neurons.

(F) DOR is expressed by cholinergic V0c neurons (white arrowheads). CC, central canal.

(G and H) Expression of DOR in Clarke's column neurons that project to the cerebellum in naive (G) and SNC80-treated (H) DORGFP mice.

Scale bars represent 50 μM. See also Figure S5.

We next asked whether DOR is also expressed by motor neurons, identified by their location and labeling with an anti-choline acetyltransferase (ChAT) antibody (Figure 4E). We found that DOR is very rarely expressed by motor neurons (2.8%). On the other hand, we observed that a different class of large ChAT+ neurons, located in laminae VII/X just lateral to the central canal and that are likely partition $V0_c$ neurons, express DOR (Figure 4F). Next, we examined thoracic sections and noticed a prominent cluster of very large DOR+ neurons, dorsal but in close proximity to $V0_c$ neurons. These likely correspond to Clarke's column neurons (Figure 4G), which are glutamatergic neurons that receive input from proprioceptors and relay this information to the cerebellum via the dorsal spinocerebellar tract. To confirm the identity of this population of DOR+ neurons, we microinjected the retrograde tracer fluorogold (FG) into the cerebellum of DORGFP mice. This procedure successfully labeled Clarke's

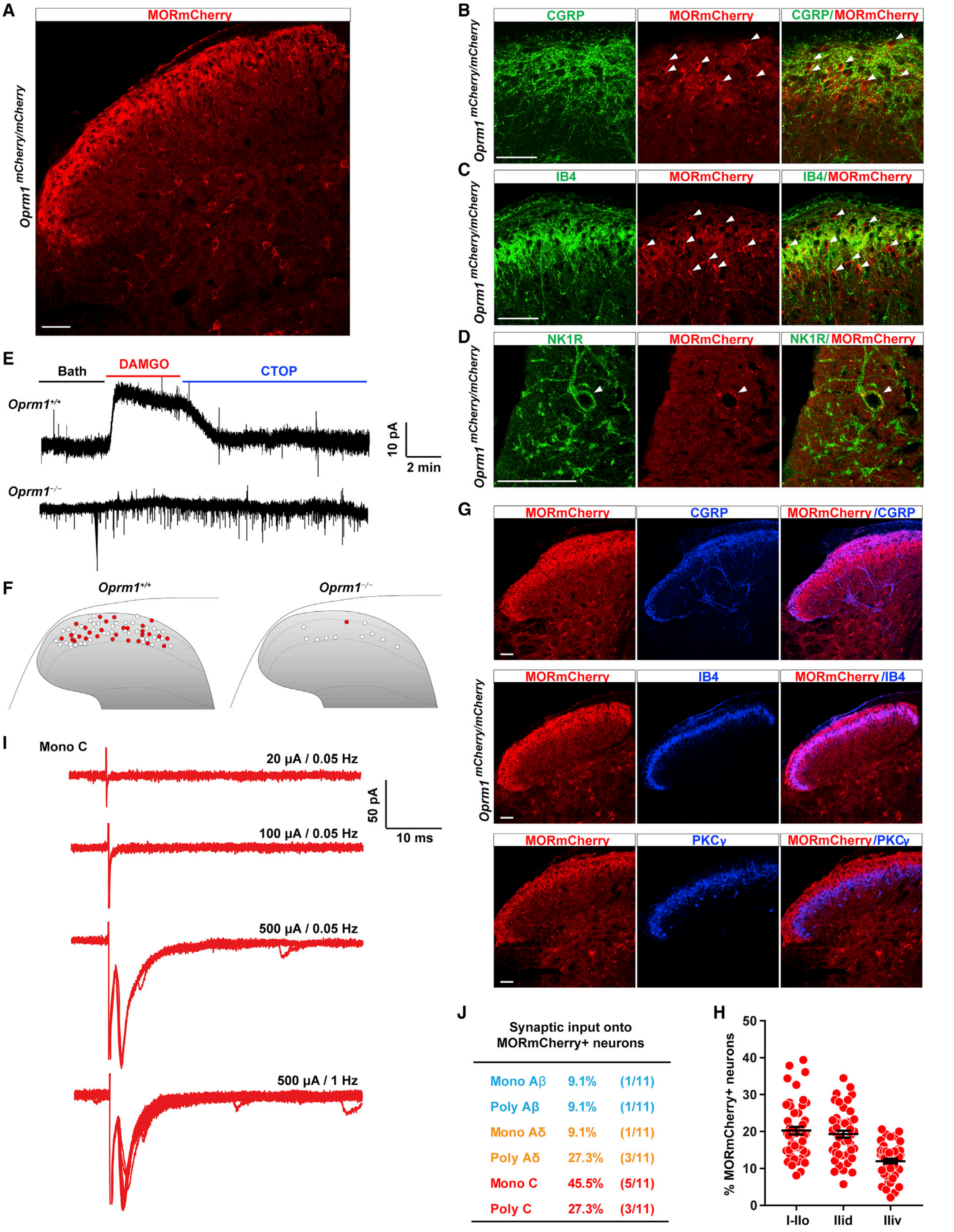

(legend on next page)

column neurons in thoracic and L1-L2 lumbar segments and as expected many were DORGFP+ (Figure 4H). Interestingly, DOR is also highly expressed in other pre-cerebellar nuclei in the brain, namely the pontine and lateral reticular nuclei (Pn and LRt), but not the inferior olive (Figures S5A–S5C). Consistent with these observations, DOR is present in mossy fiber terminals that synapse onto cerebellar granule cells, but not in the molecular layer where inferior olive-derived climbing fibers terminate (Figure S5D).

Somewhat unexpectedly, these results indicate that DOR is expressed in multiple classes of neurons that critically regulate spinal motor control. We propose that this expression pattern underlies the pathophysiological effects of DOR agonists on motor coordination (Beaudry et al., 2009; Jutkiewicz et al., 2005) for which underlying mechanisms had proven elusive.

DORs and MORs Are Largely Segregated in Lamina II Excitatory Interneurons

Our finding of DOR expression in spinal neurons suggested that the previously proposed DOR-MOR interactions could occur within spinal neurons. Indeed, several studies demonstrated that MOR is present in dorsal horn neurons, both in lamina II excitatory interneurons and in lamina I projection neurons (Aicher et al., 2000; Kemp et al., 1996; Marker et al., 2006). Whether and to what extent MOR is also expressed by other types of spinal neurons is less clear. Therefore, we next used electrophysiological and histological techniques, both in wild-type and in a MORmCherry (*Oprm1*$^{mCherry/mCherry}$) reporter mouse (Erbs et al., 2015), to resolve the MOR expression pattern in the spinal cord.

Immunolabeling with anti-RFP antibodies in MORmCherry mice revealed the location of MOR+ cell bodies, without the requirement of agonist-induced internalization. Consistent with published MOR expression studies performed in wild-type mice and rats, we observed dense MORmCherry expression in the superficial dorsal horn, in DRG neuron terminals as well as in lamina II interneurons (Figures 5A–5C) and NK1R+ lamina I projection neurons (Figure 5D). Electrophysiological studies in slices from wild-type mice consistently showed that the selective MOR agonist DAMGO induced GIRK-mediated currents in a subpopulation of superficial dorsal horn neurons (40.8%, 29/71) (Figures 5E and 5F). These DAMGO-induced currents were almost completely absent in MOR KO mice (1/11) (Figures 5E and 5F).

Interestingly, we found that the MORmCherry+ neurons in the dorsal horn occupy a location more dorsal compared to DOR+ lamina IIiv interneurons. Thus, MORmCherry+ neurons are particularly enriched in lamina II outer and in the dorsal part of lamina II inner, where CGRP+ and IB4+ DRG neurons terminate, respectively, dorsal to the band of PKCγ interneurons (Figures 5B, 5C, 5G, and 5H). In agreement with this observation, MORmCherry+ lamina II neurons receive C and Aδ fiber input (presumably C and AM nociceptors) (Figures 5I and 5J), in contrast to the lamina II DOR+ neurons that receive inputs mostly from Aδ and Aβ fibers (presumably AMs and A-LTMRs) (Figures 3C and 3D). As for DOR, however, lamina II MOR+ neurons are often Penk+ and/or occasionally postsynaptic to Penk+ neurons (Figures S4B–S4E).

This differential pattern strongly suggested that DOR and MOR are expressed by distinct populations of excitatory interneurons. To directly test this possibility, we crossed DORGFP and MORmCherry reporter mice. Figure 6A shows that indeed the majority of MORmCherry+ neurons differ from, and are located dorsally to, DORGFP+ lamina IIiv interneurons. Note, however, that DOR and MOR are occasionally co-expressed in a population of lamina II interneurons (16.5%), as shown in Figure 6B. We confirmed these results in both wild-type mice and in DORGFP mice, which have a wild-type *Oprm1* allele (Figures 6C–6F). In slices from wild-type mice, we found very few laminae I-II neurons with GIRK-mediated currents following bath perfusion of both DAMGO and deltorphin II (9.4%, 5/53) (Figures 6C–6E). Furthermore, the majority of DORGFP+ interneurons are not labeled with an anti-MOR antibody (Figure 6F).

DOR and MOR Are Co-expressed in Some Lamina I Projection Neurons and in V1 Ventral Horn Interneurons

We next labeled projection neurons in DORGFP;MORmCherry (*Oprd1*$^{gfp/gfp}$/*Oprm1*$^{mCherry/mCherry}$) mice by microinjecting FG in the lateral parabrachial nucleus (Figure 6G). Remarkably, we found that multiple subpopulations of projection neurons can be defined based on DOR and/or MOR expression and that DOR and MOR are frequently co-expressed by lamina I projection neurons. Thus, the great majority of lamina I projection neurons express MORmCherry, and among these neurons about 58.0% co-expresses DORGFP. Overall, half of the projection neurons express both receptors (50.0%), 36.1% express only MORmCherry, and very few express only DORGFP (6.0%) or neither receptor (7.8%) (Figure 6G). Co-immunostaining for

Figure 5. Distribution of MOR-Expressing Spinal Neurons

(A) Staining with an anti-RFP antibody in spinal cord sections from MORmCherry knockin mice shows MOR presence in primary afferent terminals.

(B) Double labeling of RFP and CGRP shows MORmCherry+ neurons (white arrowheads) present in laminae I and II outer.

(C) MORmCherry+ neurons (white arrowheads) are also found in lamina II inner dorsal where IB4+ primary afferent project.

(D) MOR is also expressed by lamina I NK1R+ neurons (white arrowhead).

(E) Representative traces of MOR agonist DAMGO induced GIRK channel activation in spinal dorsal horn neurons from wild-type or MOR knockout mice. The outward current can be blocked by the MOR antagonist CTOP.

(F) Schematic map showing the location of all recorded dorsal horn neurons from wild-type (n = 71) or MOR knockout (n = 11) mice. DAMGO-induced GIRK currents were observed in those neurons filled with red.

(G) MOR+ neuron distribution in dorsal horn laminae I and II outer (CGRP), lamina II inner dorsal (IB4), and lamina II inner ventral (PKCγ).

(H) Quantification of (G). Data are presented as mean ± SEM with dots showing individual counts in spinal cord sections from 3 mice.

(I and J) Sample traces (I) show C fiber input to MORmCherry+ neurons (n = 11). Summary table (J) indicates that the majority of MORmCherry+ neurons receive input from C/Aδ, but not Aβ, fibers.

Scale bars represent 50 μM. See also Figure S4.

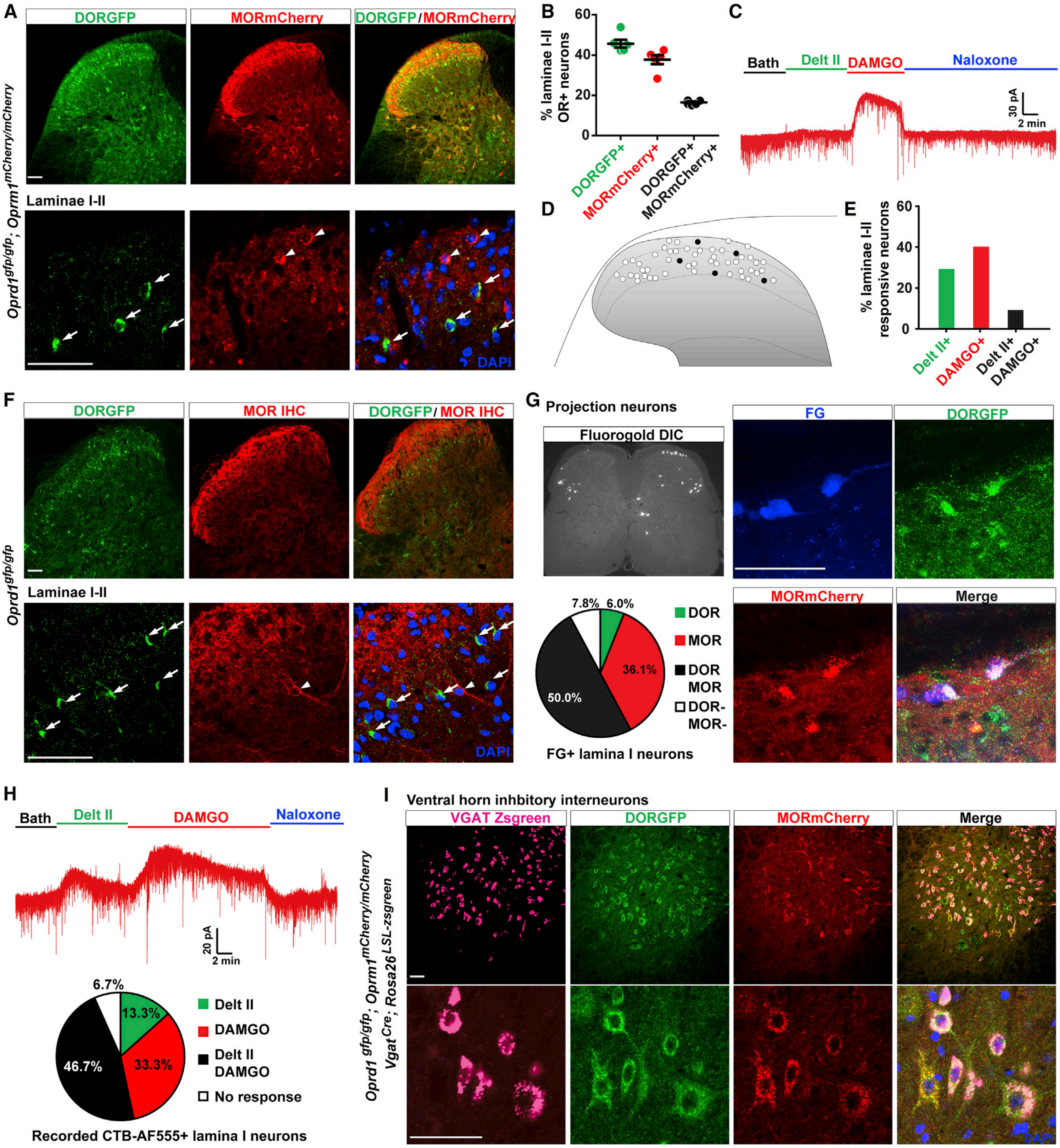

Figure 6. Spinal Neurons Co-expressing DOR and MOR

(A) Limited co-expression of DOR and MOR in laminae I-II dorsal horn neurons. White arrows indicate DORGFP+ neurons, and arrowheads indicate MORmCherry+ neurons.

(B) Quantification of (A). Data are presented as mean ± SEM from 6 mice.

(C) Representative trace showing that DAMGO and deltorphin II very rarely induced GIRK channel activation in the same lamina II neurons.

(D and E) Schematic map (D) and bar graph (E) showing the location and proportion of the neurons that responded to both deltorphin II and DAMGO (black) in wild-type mice.

(F) Immunostaining for DORGFP and MOR in DORGFP mice confirms the segregated expression of DOR and MOR in the dorsal horn.

(G) Co-expression of DOR and MOR in lamina I projection neurons (n = 166 neurons from 3 mice).

(legend continued on next page)

NK1R confirmed that both opioid receptors are present in 55.5% of NKIR+ lamina I neurons (Figures S6A and S6B). Electrophysiological recordings in wild-type mice also support the co-expression of DOR and MOR in projection neurons (Figure 6H). Sequential application of deltorphin II and DAMGO induced GIRK currents in response to both agonists in 46.7% (7/15) of CTB-AF555+ lamina I neurons. 33.3% (5/15) displayed only DAMGO-mediated GIRK currents, 13.3% (2/15) only deltorphin II-induced currents, and 6.7% (1/15) did not respond to either agonist. Interestingly, noxious mechanical stimulus-induced Fos expression predominated in the projection neurons that co-expressed DORGFP and MORmCherry (65.0%), whereas a noxious heat stimulus primarily induced Fos in MORmCherry+ projection neurons (56.1%) (Figures S6C and S6D).

Note, however, that projection neurons account for no more than 5% of neurons in lamina I and represent an extremely small percentage of all spinal neurons (Todd, 2010). Given the great predominance of other neuronal and glial cell types, the co-immunoprecipitation studies that reported direct DOR-MOR interactions in spinal cord tissue were likely not adequately sensitive to detect these interactions if they originated solely from this projection neuron population. Because of the high prevalence of DOR in ventral horn inhibitory interneurons, we reasoned that if MOR is present in the same ventral horn neuronal population, then DOR and MOR co-expression in spinal cord may, in fact, prevail in motor circuits. Strikingly, we found that the majority of the large populations of DORGFP+ ventral horn neurons do indeed co-express MORmCherry (55.5%). Furthermore, we found that 65.8% of DORGFP+MORmCherry+ neurons in the ventral horn expressed VGAT (Figure 6I), suggesting that they mostly correspond to V1 inhibitory neurons. Finally, we found that MORmCherry, as for DORGFP, is rarely expressed by motoneurons; and based on their cell body diameters, DORGFP and MORmCherry are segregated in α and γ motoneurons, respectively (Figures 4E and S6E). MORmCherry is also rarely expressed by Clarke's column or other cholinergic neurons (Figures S6F and S6G), in contrast to DOR.

DOR and MOR Organization in Brain Circuits that Mediate Pain Affect

Opioid analgesics act not only at the DRG and spinal levels, but also in the brain, where they notably interfere with the affective component of pain experience. The lateral parabrachial nucleus (LPB) represents the major brain output of spinal projection neurons of the anterolateral tract (Todd, 2010). Remarkably, we found that MORmCherry and DORGFP are expressed in distinct subnuclei of the LPB, with DOR being predominantly present in the dorsal part of the LPB (LPBD), while MOR is mostly expressed in the external part of the LPB (LPBE) (Figure 7A). The amygdala and anterior cingulate cortex (ACC) are considered key structures for the aversive quality of pain. Figures 7B and 7C shows that the segregated model of DOR and MOR expression seen in the DRG and dorsal horn extends to CNS circuits that underlie pain affect. This divergent expression is most evident in the amygdala (Figure 7B), where MOR is densely expressed by neurons of the capsular part of the central amygdala (CeC) and by intercalated (ITC) cells, while DOR+ neurons are mostly found in the basolateral amygdala (BLA). BLA neurons are predominantly excitatory, receive inputs from cortical and subcortical regions for attribution of emotional valence and motivational significance to noxious stimuli, and transmit this information to the central amygdala. In contrast, the central amygdala predominantly contains inhibitory neurons and is the major output nucleus of the amygdala. It is clear, therefore, that DOR and MOR have distinct functions in amygdalar circuits modulating pain affect. Similarly, DORGFP+ and MORmCherry+ neurons are segregated in different cortical layers of the ACC (Figure 7C). DOR is enriched in ACC laminae II/III, while MOR predominates in layer V. In sharp contrast, and following the functional organization pattern that we observed in spinal motor circuits, almost all neurons of the pre-cerebellar Pn and LRt co-express DORGFP and MORmCherry (Figures S7A and S7B).

DORs and MORs Internalize Independently in Nociceptive Spinal Neurons

In contrast to DOR, which is trafficked to lysosomes and degraded, MOR is largely recycled to the membrane and resensitized after agonist-induced internalization (Tsao and von Zastrow, 2000; Whistler et al., 2002; Williams et al., 2013). Previous trafficking studies, which relied primarily on *in vitro* heterologous expression systems, proposed that DOR agonists regulate the antinociceptive action of a MOR agonist by causing MOR co-internalization and co-degradation with activated DORs (He et al., 2011). Our identification of the lamina I-II nociceptive neurons that co-express both receptors enabled asking whether DOR and MOR co-trafficking indeed occurs *in vivo*.

We first examined the impact of SNC80 pre-treatment, which induces DORGFP internalization and trafficking to lysosomes (Pradhan et al., 2009; Scherrer et al., 2006), on MOR cellular distribution in DOR-MOR co-expressing dorsal horn neurons, using immunostaining with anti-GFP and anti-MOR antibodies. Figure 8A shows that in control conditions DOR and MOR coexist at the surface of the rare dorsal horn neurons that co-expresses both receptors. SNC80 triggered robust DORGFP internalization and accumulation in large-diameter vesicles in the perinuclear region, consistent with its trafficking to lysosomes for degradation (Figures 8A and 8B). Most importantly, however, SNC80 treatment had no obvious impact on the distribution of MORs, which rather than being co-internalized with DORGFP receptors, remained at the cell surface. Because the presence of the GFP-tag in the DORGFP mice could alter co-internalization and co-degradation mechanisms, we repeated these experiments in wild-type mice and again found that SNC80 did not cause MOR trafficking to lysosomes (Figure S8A). These observations

(H) Representative trace showing that both deltorphin II and DAMGO induced GIRK channel activation in a lamina I projection neuron. Summarized data from 15 recorded projection neurons indicates that 46.7% of them responded to both agonists.

(I) Triple labeling with an anti-GFP antibody, an anti-RFP antibody, and VGAT-Zsgreen in sections from *Oprd1*$^{gfp/gfp}$;*Oprm1*$^{mCherry/mCherry}$;*Vgat*^{Cre};*Rosa26*$^{LSL\text{-}Zsgreen}$ mice indicating that DOR and MOR are highly co-expressed in ventral horn inhibitory interneurons.

Scale bars represent 50 μM. See also Figure S6.

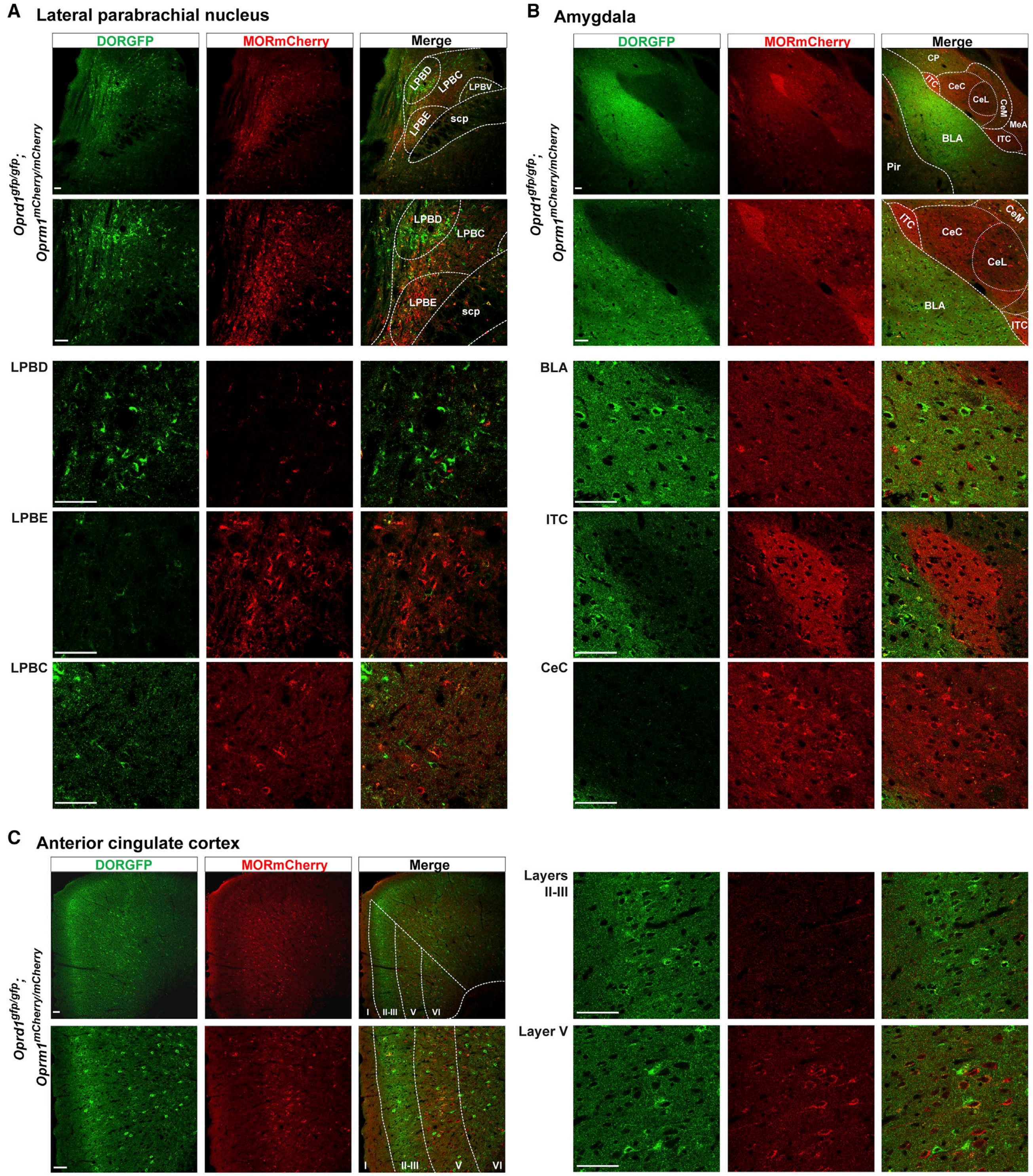

Figure 7. DOR and MOR Divergent Distribution in Brain Pain Circuits

(A) DOR and MOR are expressed by neurons located in different subnuclei of the lateral parabrachial nucleus. LPBD, lateral parabrachial nucleus, dorsal; LPBE, lateral parabrachial nucleus, external; LPBC, lateral parabrachial nucleus, central; LPBV, lateral parabrachial nucleus, ventral; scp, superior cerebellar peduncle.

(B) Segregated expression of DOR- and MOR-expressing neurons in distinct amygdalar nuclei. BLA, basolateral amygdala; ITC, intercalated cells; CeC, central amygdala, capsular; CeM, central amygdala, medial; CeL, central amygdala, lateral; MeA, medial amygdala; Pir, piriform cortex; CP, caudate putamen.

(C) Preferential distribution of neurons expressing DOR or MOR in layer II/III and V of the anterior cingulate cortex, respectively.

Scale bars represent 50 μM. See also Figure S7.

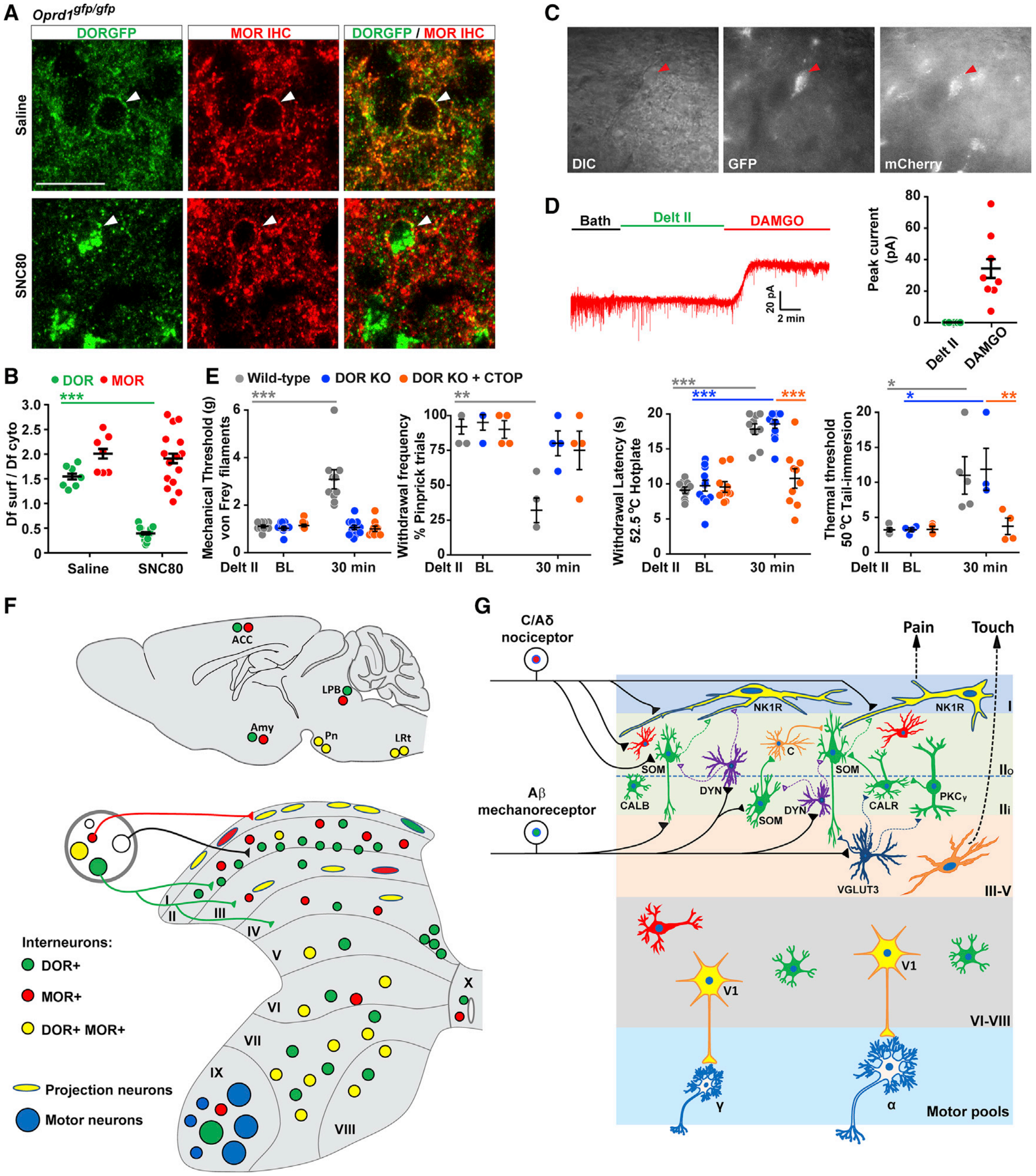

Figure 8. MOR Is Not Co-internalized and Co-degraded with DOR in Response to DOR Agonists

(A) Co-staining with anti-GFP and anti-MOR antibodies in spinal cord sections from DORGFP mice indicates that the DOR agonist SNC80 (10 mg/kg, s.c., 2 hr before tissue collection) causes DOR, but not MOR, internalization. White arrowheads indicate DOR+ MOR+ neurons. Scale bars represent 20 μM.

(B) Quantification of (A). Data are presented as mean ± SEM with dots showing individual neurons from 3 mice. ***$p < 0.001$, one-way ANOVA.

(C) DOR+MOR+ neurons are visually identified by GFP and mCherry fluorescence prior to recording.

(D) Despite SNC80 treatment, DAMGO, but not deltorphin II, still causes GIRK channels activation in spinal neurons that co-express DOR and MOR (n = 8).

(E) Effect of intrathecal deltorphin II (1 μg) against mechanical and heat nociception in wild-type mice. Co-injection of the MOR antagonist CTOP blocked the residual deltorphin II-induced antinociception against heat pain in DOR global knockout mice. Data are presented as mean ± SEM, *$p < 0.05$, **$p < 0.01$, ***$p < 0.001$, repeated-measures, two-way ANOVA + Bonferroni. Von Frey threshold and hotplate tests: n = 10 wild-type mice, 13 DOR KO mice, and 10 DOR KO+CTOP mice; pinprick and tail immersion tests: n = 5 wild-type mice, 4 DOR KO mice, and 4 DOR KO+CTOP mice.

(F) DOR and MOR distribution map in pain and motor circuits. DOR is expressed by numerous populations of spinal neurons, including laminae I-II interneurons, Clarke's column neurons, Renshaw cells, ChAT+ V0c, and V1 inhibitory neurons in the ventral horn. DOR and MOR are often co-expressed in lamina I projection neurons and V1 inhibitory neurons. In the brain, DORs and MORs are predominantly segregated in neurons of the lateral parabrachial nucleus (LPB), amygdala (Amy), and anterior cingulate cortex (ACC) but are abundantly co-expressed in precerebellar motor control regions including the pontine nucleus (Pn) and lateral reticular nucleus (LRt).

(legend continued on next page)

suggest that *in vivo*, MOR is not co-internalized and co-degraded with DOR in lysosomes. Similarly, intrathecal DAMGO caused the internalization of MOR, presumably in recycling endosomes beneath the plasma membrane (Williams et al., 2013), without affecting the DORGFP signal, which remained at the cell surface (Figures S8B and S8C).

We next used electrophysiology to examine the impact of DOR trafficking to lysosomes on MOR function. Based on the co-degradation hypothesis (He et al., 2011), one would predict that SNC80 treatment should eliminate subsequent neuronal responses to both DOR and MOR agonists. As expected, we found that in slices from mice treated with SNC80, deltorphin II no longer activates GIRK currents in lamina I-II neurons that co-express DORGFP and MORmCherry (0%, 0/8) (Figures 8C and 8D). Remarkably, however, DAMGO-induced GIRK currents were intact in these neurons (Figure 8D). Together with our histological findings, these data suggest that DOR trafficking to and degradation in lysosomes does not profoundly impact MOR function *in vivo* in cells that co-express both receptors (see Discussion). Of note, we attempted to directly probe the function of potential DOR-MOR heteromers using the heteromer-biased agonist CYM51010 (Gomes et al., 2013). However, control recordings in DOR KO mice revealed that this compound can cause the activation of GIRK currents in the absence of DOR (Figures S9A–S9C).

The distinct expression pattern and trafficking properties of DOR and MOR in dorsal horn neurons suggests that these receptors may have different functions in opioid-mediated pain control. Our previous studies using dose responses of SNC80 (Scherrer et al., 2009) and the current analysis indicated that DOR+ neurons in primary afferents and in the spinal cord predominate in circuits that regulate mechanical pain. On the other hand, multiple studies showed that deltorphin II can increase the threshold for not only mechanical but also heat pain (Beaudry et al., 2009; Cahill et al., 2001; Gavériaux-Ruff and Kieffer, 2011; Gendron et al., 2007, 2015; Normandin et al., 2013; Porreca et al., 2003; Schuster et al., 2015). To address this discrepancy, we next assessed the effect of intrathecal deltorphin II on mechanical and heat pain both in wild-type and global DOR KO mice. Consistent with previous studies, deltorphin II significantly elevated both mechanical and heat thresholds in wild-type mice. However, while deltorphin II-induced mechanical antinociception was completely lost in DOR KO mice, deltorphin II-mediated elevation of the heat pain threshold was intact (Figure 8E). These opposing data from DOR KO mice and wild-type mice suggest that *in vivo*, at the doses commonly used in the literature, intrathecal deltorphin II can cross activate other opioid receptors in addition to activating DOR. In support of this idea, we found that deltorphin II-mediated antinociception against noxious heat is blocked by the intrathecal co-injection of the MOR antagonist CTOP, indicating that the broadly observed effect of intrathecal deltorphin II against heat pain is dependent on MOR (Figure 8E).

In summary, our analysis establishes that few spinal nociceptive neurons co-express DOR and MOR and that in the rare neurons in which they are co-expressed, the two receptors internalize and function independently. The schematics in Figures 8F and 8G highlight the segregated distribution and function of DOR and MOR in major populations of primary sensory, spinal, and brain neurons constituting pain circuits.

DISCUSSION

Functional Organization of DOR and MOR in Pain Circuits

The spinal cord dorsal horn is an essential integrative center that processes and transmits pain information to the brain (Basbaum et al., 2009; Braz et al., 2014; Todd, 2010). MOR expression in spinal neurons was described mainly in laminae I-II (Aicher et al., 2000; Kemp et al., 1996; Spike et al., 2002; Trafton et al., 2000). Earlier work using the anti-DOR$^{3-17}$ antibody (Ab$^{3-17}$) suggested that DOR was only expressed at the central terminals of peptidergic afferents and absent from spinal cord neurons (Dado et al., 1993). We demonstrate here that both DOR and MOR are broadly distributed in a variety of spinal interneurons and projection neurons.

We used agonist-induced receptor internalization in DORGFP mice to reveal the distribution and identity of DOR+ neurons in the spinal cord. We confirmed this DOR distribution pattern in wild-type mice, by detecting *Oprd1* transcripts and DOR agonist-induced GIRK currents in spinal neurons. Interestingly, DOR expression in spinal neurons may explain previous data reporting inhibition of substance P (SP) release by intrathecal DOR agonists (Beaudry et al., 2011; Kouchek et al., 2013), despite the segregated expression of SP and DOR in primary afferents (Bardoni et al., 2014; Mennicken et al., 2003; Minami et al., 1995; Scherrer et al., 2009; Usoskin et al., 2015). SP, like DOR, is expressed by a population of excitatory interneurons in the dorsal horn (Gutierrez-Mecinas et al., 2017; Xu et al., 2013). Therefore, DOR agonists might directly act on DOR+ SP+ dorsal horn neurons or other DOR+ connected CNS neurons, rather than on primary afferents, to depress SP spinal release.

(G) Schematic showing DOR and MOR distribution and function in dorsal horn pain circuits. DOR is expressed by multiple types of excitatory interneurons in lamina II, including those expressing SOM, CALB, CALR, or PKCγ. The DOR+ SOM+ neurons located in lamina IIi ventral predominantly receive Aβ mechanoreceptor input and polysynaptically transmit this information to lamina I projection neurons, including those expressing DOR and/or MOR, via lamina II transient-central cells and vertical cells. The lamina IIo vertical DOR+SOM+ neurons may receive synaptic input from mechanonociceptors, and activity in this pathway may be controlled by inhibitory neurons expressing dynorphin (DYN+) (Duan et al., 2014). In the condition of injury-induced inflammatory or neuropathic pain, lamina III VGLUT3+ neurons may receive Aβ input (Peirs et al., 2015) and could engage DOR+ neurons in lamina IIi (including DOR+ PKCγ+ or DOR+ CALR+), relaying this information dorsally to nociceptive neurons for the emergence of mechanical allodynia. In contrast, the populations of lamina I MOR+ and DOR+ MOR+ projection neurons may receive monosynaptic C/Aδ nociceptive inputs from MOR+ peptidergic DRG neurons and myelinated mechanonociceptors to transmit both heat and mechanical acute pain information. In the ventral horn, DOR and MOR are co-expressed by V1 inhibitory interneurons, including Renshaw cells, suggesting a function in motor output regulation. SOM, somatostatin; DYN, dynorphin; CALR, calretinin; CALB, calbindin; PKCγ, protein kinase Cγ; C, transient-central cells; VGLUT3, vesicular glutamate transporter type 3.
See also Figures S8 and S9.

Most importantly, we found that DOR+ neurons in the dorsal horn mainly correspond to the most ventral subpopulation of SOM+ lamina II excitatory interneurons and to preferentially mechanoresponsive lamina I nociceptive projection neurons. Previous ablation and inhibitory chemogenetic studies of SOM+ dorsal horn neuron function showed that these cells are critical to acute mechanical pain and injury-induced mechanical hypersensitivity (Christensen et al., 2016; Duan et al., 2014). We show here that DOR activation in these cells causes membrane hyperpolarization and reduces action potential firing via GIRK channel opening. Therefore, DOR is optimally distributed in dorsal horn circuits, both presynaptically at the terminals of mechanosensory neurons and centrally in SOM+ neurons, to selectively control transmission of mechanical pain information to the brain. We propose that DOR agonists reduce excitability of SOM+ interneurons, mimicking the consequences of SOM+ neuron ablation or chemogenetic inhibition (Christensen et al., 2016; Duan et al., 2014), to limit injury-induced pathological polysynaptic neurotransmission between low-threshold mechanoreceptor inputs in laminae IIiv-IV and lamina I nociceptive projection neurons (Figure 8G). We recently described a descending pain control circuit involving the spinal release of the endogenous DOR and MOR agonist enkephalin (Francois et al., 2017) and the pronociceptive maladaptive function of MOR in TRPV1+ nociceptors during prolonged agonist treatment (Corder et al., 2017). Future studies will dissect the contribution of the different populations of dorsal and ventral horn DOR and MOR receptor populations to enkephalinergic pain and motor control and will determine whether presynaptic DOR may also induce maladaptive plasticity in mechanosensory neurons with sustained DOR agonist exposure.

DOR and MOR Cooperation in Nociceptive Neurons *In Vivo*

Previous studies suggested that interfering with DOR activity could enhance morphine analgesia and reduce antinociceptive tolerance (Abdelhamid et al., 1991; Chefer and Shippenberg, 2009; Daniels et al., 2005; Fujita et al., 2015; Gomes et al., 2004; Schiller, 2005; Zhu et al., 1999). These findings were often interpreted as resulting from functional interactions between DOR and MOR, including in the same cells through heteromerization. *In vitro* studies using heterologous expression systems or spinal cord tissue also provided evidence that the two receptors may directly interact and form heteromers (Fan et al., 2005; Gomes et al., 2004). For example, HEK293 cells overexpressing Myc-DOR and HA-MOR constructs were used to investigate cooperation between the two receptors (He et al., 2011). These studies suggested that DOR agonists SNC80 or deltorphin II can produce co-internalization and co-degradation of MOR, which could attenuate morphine analgesia and contribute to tolerance.

Here we make several key observations *in vivo* that lead us to reconsider the physiological importance of these earlier findings. First, we found that DOR and MOR are expressed in largely distinct neurons along pain circuits. Second, we found that DOR activation and internalization affects neither MOR presence at the surface of neurons co-expressing both receptors *in vivo*, nor the ability of DAMGO to activate GIRK channels, arguing against MOR lysosomal co-degradation with DOR. Therefore, it is unlikely that DOR intracellular control of MOR function defines morphine-induced antinociception and adaptations associated with tolerance. Note, however, that given the unknown sensitivity of immunohistological assays and reporter mouse lines, that the activation of a small fraction of MORs can lead to maximal activation of GIRK currents, and since direct receptor interactions might have other consequences than co-degradation, our results do not rule out the existence of DOR-MOR heteromers. Additionally, DOR and MOR can influence each other's function through other molecular and cellular mechanisms in discrete neuronal populations or at the circuit level in pain networks. The consequence of expressing both receptors, rather than only one of them, on neuronal excitability and neurotransmitter release, for a defined neuron *in vivo*, is for example completely unexplored. Determining the precise function of DOR+ MOR+ dorsal horn neurons will be necessary to formulate testable predictions regarding the relevance of DOR-MOR functional interactions for the modulation of somatosensory experience, including encoding of specific pain (e.g., cutaneous versus visceral), temperature, itch, or touch modalities. Here we provide an approach to answering these questions as we identify trackable populations of neurons that co-express DOR and MOR, including the small, but critical to pain (Mantyh et al., 1997; Nichols et al., 1999), retrogradely labeled population of nociceptive NK1R+ lamina I projection neurons. Alternatively, the genetically defined large population of En1+ ventral horn neurons may represent another ideal population for mechanistic studies of DOR-MOR cellular and molecular interactions (Figure 8F).

In conclusion, we resolve here the functional organization of DOR and MOR, the two main opioid receptors for the control of neurotransmission in pain circuits. This study provides the fundamental basis for understanding the mechanism of action of endogenous and exogenous opioids to develop improved opioid analgesics, an urgent need given the chronic pain and opioid epidemics (Institute of Medicine, 2011; Volkow and McLellan, 2016).

STAR★METHODS

Detailed methods are provided in the online version of this paper and include the following:

- KEY RESOURCES TABLE
- CONTACT FOR REAGENT AND RESOURCE SHARING
- EXPERIMENTAL MODEL AND SUBJECT DETAILS
- METHOD DETAILS
    - Drugs Administration
    - Immunohistochemistry
    - *In Situ* Hybridization
    - Subcellular Fluorescence Density
    - Noxious Mechanical and Thermal Stimulation
    - Genomic DNA Analysis
    - Retrograde Tracing
    - Spinal Cord Slice Preparation and Electrophysiology
    - Behavioral Tests
- QUANTIFICATION AND STATISTICAL ANALYSIS

SUPPLEMENTAL INFORMATION

Supplemental Information includes nine figures and can be found with this article online at https://doi.org/10.1016/j.neuron.2018.03.002.

ACKNOWLEDGMENTS

This work was supported by National Institutes of Health grants DA031777 and DA044481 (G.S.), DA029204 and NS097306 (A.I.B.), a Department of Defense Neurosensory Award MR130053 (G.S.), a Welcome Trust award A102645 (A.I.B), a Rita Allen Foundation and American Pain Society Award in Pain (G.S.), NIH fellowships T32GM089626 (D.W.), T32DA035165 (D.W. and G.C.), F32DA041029, and K99DA043609 (G.C.), a Foundation for Anesthesia Education and Research (FAER) Mentored Research Training Grant (V.L.T.) and an HHMI medical research fellowship (S.A.L.). G.S. is a New York Stem Cell Foundation – Robertson Investigator. We thank Brigitte Kieffer for providing *Oprd1*$^{egfp/egfp}$, *Oprd1*$^{lox/lox}$, and *Oprm1*$^{mcherry/mcherry}$ reporter mice, Tom Jessell for providing *En1*^{Cre}*; Rosa26*$^{LSL\text{-}tdTomato}$ mice, and David Julius for the anti-P2Y12 antibody.

AUTHOR CONTRIBUTIONS

D.W. performed spinal cord slice electrophysiology, retrograde tracing, and agonist-induced receptor internalization experiments. D.W., V.L.T., and S.A.L. performed Immunohistochemistry. D.W. and V.L.T. performed *in situ* hybridization. G.C. and D.W. performed behavioral pharmacology. A.F. preformed studies in *Penk*^{Cre} mice. D.W., V.L.T., A.I.B., and G.S. designed studies and wrote the manuscript. All authors contributed to data interpretation and editing of the manuscript.

DECLARATION OF INTERESTS

The authors declare no competing interests.

Received: June 29, 2017
Revised: January 19, 2018
Accepted: March 2, 2018
Published: March 22, 2018

REFERENCES

Abdelhamid, E.E., Sultana, M., Portoghese, P.S., and Takemori, A.E. (1991). Selective blockage of delta opioid receptors prevents the development of morphine tolerance and dependence in mice. J. Pharmacol. Exp. Ther. *258*, 299–303.

Abraira, V.E., Kuehn, E.D., Chirila, A.M., Springel, M.W., Toliver, A.A., Zimmerman, A.L., Orefice, L.L., Boyle, K.A., Bai, L., Song, B.J., et al. (2017). The cellular and synaptic architecture of the mechanosensory dorsal horn. Cell *168*, 295–310 e219.

Aicher, S.A., Punnoose, A., and Goldberg, A. (2000). mu-Opioid receptors often colocalize with the substance P receptor (NK1) in the trigeminal dorsal horn. J. Neurosci. *20*, 4345–4354.

Alaynick, W.A., Jessell, T.M., and Pfaff, S.L. (2011). SnapShot: spinal cord development. Cell *146*, 178–178 e171.

Arvidsson, U., Riedl, M., Chakrabarti, S., Lee, J.H., Nakano, A.H., Dado, R.J., Loh, H.H., Law, P.Y., Wessendorf, M.W., and Elde, R. (1995). Distribution and targeting of a mu-opioid receptor (MOR1) in brain and spinal cord. J. Neurosci. *15*, 3328–3341.

Bao, L., Jin, S.X., Zhang, C., Wang, L.H., Xu, Z.Z., Zhang, F.X., Wang, L.C., Ning, F.S., Cai, H.J., Guan, J.S., et al. (2003). Activation of delta opioid receptors induces receptor insertion and neuropeptide secretion. Neuron *37*, 121–133.

Bardoni, R., Tawfik, V.L., Wang, D., François, A., Solorzano, C., Shuster, S.A., Choudhury, P., Betelli, C., Cassidy, C., Smith, K., et al. (2014). Delta opioid receptors presynaptically regulate cutaneous mechanosensory neuron input to the spinal cord dorsal horn. Neuron *81*, 1312–1327.

Basbaum, A.I., Bautista, D.M., Scherrer, G., and Julius, D. (2009). Cellular and molecular mechanisms of pain. Cell *139*, 267–284.

Beaudry, H., Proteau-Gagné, A., Li, S., Dory, Y., Chavkin, C., and Gendron, L. (2009). Differential noxious and motor tolerance of chronic delta opioid receptor agonists in rodents. Neuroscience *161*, 381–391.

Beaudry, H., Dubois, D., and Gendron, L. (2011). Activation of spinal mu- and delta-opioid receptors potently inhibits substance P release induced by peripheral noxious stimuli. J. Neurosci. *31*, 13068–13077.

Braz, J., Solorzano, C., Wang, X., and Basbaum, A.I. (2014). Transmitting pain and itch messages: a contemporary view of the spinal cord circuits that generate gate control. Neuron *82*, 522–536.

Cahill, C.M., Morinville, A., Lee, M.C., Vincent, J.P., Collier, B., and Beaudet, A. (2001). Prolonged morphine treatment targets delta opioid receptors to neuronal plasma membranes and enhances delta-mediated antinociception. J. Neurosci. *21*, 7598–7607.

Chaplan, S.R., Bach, F.W., Pogrel, J.W., Chung, J.M., and Yaksh, T.L. (1994). Quantitative assessment of tactile allodynia in the rat paw. J. Neurosci. Methods *53*, 55–63.

Chefer, V.I., and Shippenberg, T.S. (2009). Augmentation of morphine-induced sensitization but reduction in morphine tolerance and reward in delta-opioid receptor knockout mice. Neuropsychopharmacology *34*, 887–898.

Christensen, A.J., Iyer, S.M., François, A., Vyas, S., Ramakrishnan, C., Vesuna, S., Deisseroth, K., Scherrer, G., and Delp, S.L. (2016). In Vivo Interrogation of Spinal Mechanosensory Circuits. Cell Rep. *17*, 1699–1710.

Corder, G., Tawfik, V.L., Wang, D., Sypek, E.I., Low, S.A., Dickinson, J.R., Sotoudeh, C., Clark, J.D., Barres, B.A., Bohlen, C.J., and Scherrer, G. (2017). Loss of μ opioid receptor signaling in nociceptors, but not microglia, abrogates morphine tolerance without disrupting analgesia. Nat. Med. *23*, 164–173.

Dado, R.J., Law, P.Y., Loh, H.H., and Elde, R. (1993). Immunofluorescent identification of a delta (delta)-opioid receptor on primary afferent nerve terminals. Neuroreport *5*, 341–344.

Daniels, D.J., Lenard, N.R., Etienne, C.L., Law, P.Y., Roerig, S.C., and Portoghese, P.S. (2005). Opioid-induced tolerance and dependence in mice is modulated by the distance between pharmacophores in a bivalent ligand series. Proc. Natl. Acad. Sci. USA *102*, 19208–19213.

Duan, B., Cheng, L., Bourane, S., Britz, O., Padilla, C., Garcia-Campmany, L., Krashes, M., Knowlton, W., Velasquez, T., Ren, X., et al. (2014). Identification of spinal circuits transmitting and gating mechanical pain. Cell *159*, 1417–1432.

Erbs, E., Faget, L., Scherrer, G., Matifas, A., Filliol, D., Vonesch, J.L., Koch, M., Kessler, P., Hentsch, D., Birling, M.C., et al. (2015). A mu-delta opioid receptor brain atlas reveals neuronal co-occurrence in subcortical networks. Brain Struct. Funct. *220*, 677–702.

Fan, T., Varghese, G., Nguyen, T., Tse, R., O'Dowd, B.F., and George, S.R. (2005). A role for the distal carboxyl tails in generating the novel pharmacology and G protein activation profile of mu and delta opioid receptor hetero-oligomers. J. Biol. Chem. *280*, 38478–38488.

Fields, H. (2004). State-dependent opioid control of pain. Nat. Rev. Neurosci. *5*, 565–575.

Filliol, D., Ghozland, S., Chluba, J., Martin, M., Matthes, H.W., Simonin, F., Befort, K., Gavériaux-Ruff, C., Dierich, A., LeMeur, M., et al. (2000). Mice deficient for delta- and mu-opioid receptors exhibit opposing alterations of emotional responses. Nat. Genet. *25*, 195–200.

François, A., and Scherrer, G. (2017). Delta Opioid Receptor Expression and Function in Primary Afferent Somatosensory Neurons. Handb. Exp. Pharmacol. Published online October 10, 2017. https://doi.org/10.1007/164_2017_58.

Francois, A., Low, S.A., Sypek, E.I., Christensen, A.J., Sotoudeh, C., Beier, K.T., Ramakrishnan, C., Ritola, K.D., Sharif-Naeini, R., Deisseroth, K., et al. (2017). A brainstem-spinal cord inhibitory circuit for mechanical pain modulation by GABA and enkephalins. Neuron *93*, 822–839 e826.

Fujita, W., Gomes, I., and Devi, L.A. (2015). Heteromers of μ-δ opioid receptors: new pharmacology and novel therapeutic possibilities. Br. J. Pharmacol. *172*, 375–387.

Gavériaux-Ruff, C., and Kieffer, B.L. (2011). Delta opioid receptor analgesia: recent contributions from pharmacology and molecular approaches. Behav. Pharmacol. *22*, 405–414.

Gaveriaux-Ruff, C., Nozaki, C., Nadal, X., Hever, X.C., Weibel, R., Matifas, A., Reiss, D., Filliol, D., Nassar, M.A., Wood, J.N., et al. (2011). Genetic ablation of delta opioid receptors in nociceptive sensory neurons increases chronic pain and abolishes opioid analgesia. Pain *152*, 1238–1248.

Gendron, L., Pintar, J.E., and Chavkin, C. (2007). Essential role of mu opioid receptor in the regulation of delta opioid receptor-mediated antihyperalgesia. Neuroscience *150*, 807–817.

Gendron, L., Mittal, N., Beaudry, H., and Walwyn, W. (2015). Recent advances on the δ opioid receptor: from trafficking to function. Br. J. Pharmacol. *172*, 403–419.

Gomes, I., Gupta, A., Filipovska, J., Szeto, H.H., Pintar, J.E., and Devi, L.A. (2004). A role for heterodimerization of mu and delta opiate receptors in enhancing morphine analgesia. Proc. Natl. Acad. Sci. USA *101*, 5135–5139.

Gomes, I., Fujita, W., Gupta, A., Saldanha, S.A., Negri, A., Pinello, C.E., Eberhart, C., Roberts, E., Filizola, M., Hodder, P., and Devi, L.A. (2013). Identification of a μ-δ opioid receptor heteromer-biased agonist with antinociceptive activity. Proc. Natl. Acad. Sci. USA *110*, 12072–12077.

Guan, J.S., Xu, Z.Z., Gao, H., He, S.Q., Ma, G.Q., Sun, T., Wang, L.H., Zhang, Z.N., Lena, I., Kitchen, I., et al. (2005). Interaction with vesicle luminal protachykinin regulates surface expression of delta-opioid receptors and opioid analgesia. Cell *122*, 619–631.

Gutierrez-Mecinas, M., Bell, A.M., Marin, A., Taylor, R., Boyle, K.A., Furuta, T., Watanabe, M., Polgár, E., and Todd, A.J. (2017). Preprotachykinin A is expressed by a distinct population of excitatory neurons in the mouse superficial spinal dorsal horn including cells that respond to noxious and pruritic stimuli. Pain *158*, 440–456.

He, S.Q., Zhang, Z.N., Guan, J.S., Liu, H.R., Zhao, B., Wang, H.B., Li, Q., Yang, H., Luo, J., Li, Z.Y., et al. (2011). Facilitation of μ-opioid receptor activity by preventing δ-opioid receptor-mediated codegradation. Neuron *69*, 120–131.

Institute of Medicine (2011). Relieving Pain in America: A Blueprint for Transforming Prevention, Care, Education, and Research (National Academies Press).

Jutkiewicz, E.M., Kaminsky, S.T., Rice, K.C., Traynor, J.R., and Woods, J.H. (2005). Differential behavioral tolerance to the delta-opioid agonist SNC80 ([(+)-4-[(alphaR)-alpha-[(2S,5R)-2,5-dimethyl-4-(2-propenyl)-1-piperazinyl]-(3-methoxyphenyl)methyl]-N,N-diethylbenzamide) in Sprague-Dawley rats. J. Pharmacol. Exp. Ther. *315*, 414–422.

Kemp, T., Spike, R.C., Watt, C., and Todd, A.J. (1996). The mu-opioid receptor (MOR1) is mainly restricted to neurons that do not contain GABA or glycine in the superficial dorsal horn of the rat spinal cord. Neuroscience *75*, 1231–1238.

Kimmel, R.A., Turnbull, D.H., Blanquet, V., Wurst, W., Loomis, C.A., and Joyner, A.L. (2000). Two lineage boundaries coordinate vertebrate apical ectodermal ridge formation. Genes Dev. *14*, 1377–1389.

Kouchek, M., Takasusuki, T., Terashima, T., Yaksh, T.L., and Xu, Q. (2013). Effects of intrathecal SNC80, a delta receptor ligand, on nociceptive threshold and dorsal horn substance p release. J. Pharmacol. Exp. Ther. *347*, 258–264.

Li, J.L., Ding, Y.Q., Li, Y.Q., Li, J.S., Nomura, S., Kaneko, T., and Mizuno, N. (1998). Immunocytochemical localization of mu-opioid receptor in primary afferent neurons containing substance P or calcitonin gene-related peptide. A light and electron microscope study in the rat. Brain Res. *794*, 347–352.

Madisen, L., Zwingman, T.A., Sunkin, S.M., Oh, S.W., Zariwala, H.A., Gu, H., Ng, L.L., Palmiter, R.D., Hawrylycz, M.J., Jones, A.R., et al. (2010). A robust and high-throughput Cre reporting and characterization system for the whole mouse brain. Nat. Neurosci. *13*, 133–140.

Mantyh, P.W., Rogers, S.D., Honore, P., Allen, B.J., Ghilardi, J.R., Li, J., Daughters, R.S., Lappi, D.A., Wiley, R.G., and Simone, D.A. (1997). Inhibition of hyperalgesia by ablation of lamina I spinal neurons expressing the substance P receptor. Science *278*, 275–279.

Marker, C.L., Luján, R., Colón, J., and Wickman, K. (2006). Distinct populations of spinal cord lamina II interneurons expressing G-protein-gated potassium channels. J. Neurosci. *26*, 12251–12259.

Matthes, H.W., Maldonado, R., Simonin, F., Valverde, O., Slowe, S., Kitchen, I., Befort, K., Dierich, A., Le Meur, M., Dollé, P., et al. (1996). Loss of morphine-induced analgesia, reward effect and withdrawal symptoms in mice lacking the mu-opioid-receptor gene. Nature *383*, 819–823.

Mennicken, F., Zhang, J., Hoffert, C., Ahmad, S., Beaudet, A., and O'Donnell, D. (2003). Phylogenetic changes in the expression of delta opioid receptors in spinal cord and dorsal root ganglia. J. Comp. Neurol. *465*, 349–360.

Minami, M., Maekawa, K., Yabuuchi, K., and Satoh, M. (1995). Double in situ hybridization study on coexistence of mu-, delta- and kappa-opioid receptor mRNAs with preprotachykinin A mRNA in the rat dorsal root ganglia. Brain Res. Mol. Brain Res. *30*, 203–210.

Nichols, M.L., Allen, B.J., Rogers, S.D., Ghilardi, J.R., Honore, P., Luger, N.M., Finke, M.P., Li, J., Lappi, D.A., Simone, D.A., and Mantyh, P.W. (1999). Transmission of chronic nociception by spinal neurons expressing the substance P receptor. Science *286*, 1558–1561.

Normandin, A., Luccarini, P., Molat, J.L., Gendron, L., and Dallel, R. (2013). Spinal μ and δ opioids inhibit both thermal and mechanical pain in rats. J. Neurosci. *33*, 11703–11714.

Ong, E.W., and Cahill, C.M. (2014). Molecular Perspectives for mu/delta Opioid Receptor Heteromers as Distinct, Functional Receptors. Cells *3*, 152–179.

Overland, A.C., Kitto, K.F., Chabot-Doré, A.J., Rothwell, P.E., Fairbanks, C.A., Stone, L.S., and Wilcox, G.L. (2009). Protein kinase C mediates the synergistic interaction between agonists acting at alpha2-adrenergic and delta-opioid receptors in spinal cord. J. Neurosci. *29*, 13264–13273.

Peirs, C., Williams, S.P., Zhao, X., Walsh, C.E., Gedeon, J.Y., Cagle, N.E., Goldring, A.C., Hioki, H., Liu, Z., Marell, P.S., and Seal, R.P. (2015). Dorsal horn circuits for persistent mechanical pain. Neuron *87*, 797–812.

Petitjean, H., Pawlowski, S.A., Fraine, S.L., Sharif, B., Hamad, D., Fatima, T., Berg, J., Brown, C.M., Jan, L.Y., Ribeiro-da-Silva, A., et al. (2015). Dorsal horn parvalbumin neurons are gate-keepers of touch-evoked pain after nerve injury. Cell Rep. *13*, 1246–1257.

Porreca, F., Vanderah, T.W., Lai, J., and Ossipov, M.H. (2003). The delta opioid receptor subtypes and pain modulation. In The Delta Receptor, K. Chang, F. Porreca, and J. Woods, eds. (Taylor Francis), pp. 297–329.

Pradhan, A.A., Becker, J.A., Scherrer, G., Tryoen-Toth, P., Filliol, D., Matifas, A., Massotte, D., Gavériaux-Ruff, C., and Kieffer, B.L. (2009). In vivo delta opioid receptor internalization controls behavioral effects of agonists. PLoS ONE *4*, e5425.

Scherrer, G., Tryoen-Tóth, P., Filliol, D., Matifas, A., Laustriat, D., Cao, Y.Q., Basbaum, A.I., Dierich, A., Vonesh, J.L., Gavériaux-Ruff, C., and Kieffer, B.L. (2006). Knockin mice expressing fluorescent delta-opioid receptors uncover G protein-coupled receptor dynamics in vivo. Proc. Natl. Acad. Sci. USA *103*, 9691–9696.

Scherrer, G., Imamachi, N., Cao, Y.Q., Contet, C., Mennicken, F., O'Donnell, D., Kieffer, B.L., and Basbaum, A.I. (2009). Dissociation of the opioid receptor mechanisms that control mechanical and heat pain. Cell *137*, 1148–1159.

Schiller, P.W. (2005). Opioid peptide-derived analgesics. AAPS J. *7*, E560–E565.

Schuster, D.J., Metcalf, M.D., Kitto, K.F., Messing, R.O., Fairbanks, C.A., and Wilcox, G.L. (2015). Ligand requirements for involvement of PKCε in synergistic analgesic interactions between spinal μ and δ opioid receptors. Br. J. Pharmacol. *172*, 642–653.

Snyder, S.H., and Pasternak, G.W. (2003). Historical review: Opioid receptors. Trends Pharmacol. Sci. *24*, 198–205.

Spike, R.C., Puskár, Z., Sakamoto, H., Stewart, W., Watt, C., and Todd, A.J. (2002). MOR-1-immunoreactive neurons in the dorsal horn of the rat spinal cord: evidence for nonsynaptic innervation by substance P-containing primary

afferents and for selective activation by noxious thermal stimuli. Eur. J. Neurosci. *15*, 1306–1316.

Taniguchi, H., He, M., Wu, P., Kim, S., Paik, R., Sugino, K., Kvitsiani, D., Fu, Y., Lu, J., Lin, Y., et al. (2011). A resource of Cre driver lines for genetic targeting of GABAergic neurons in cerebral cortex. Neuron *71*, 995–1013.

Todd, A.J. (2010). Neuronal circuitry for pain processing in the dorsal horn. Nat. Rev. Neurosci. *11*, 823–836.

Torsney, C., and MacDermott, A.B. (2006). Disinhibition opens the gate to pathological pain signaling in superficial neurokinin 1 receptor-expressing neurons in rat spinal cord. J. Neurosci. *26*, 1833–1843.

Trafton, J.A., Abbadie, C., Marek, K., and Basbaum, A.I. (2000). Postsynaptic signaling via the [mu]-opioid receptor: responses of dorsal horn neurons to exogenous opioids and noxious stimulation. J. Neurosci. *20*, 8578–8584.

Tsao, P.I., and von Zastrow, M. (2000). Type-specific sorting of G protein-coupled receptors after endocytosis. J. Biol. Chem. *275*, 11130–11140.

Usoskin, D., Furlan, A., Islam, S., Abdo, H., Lönnerberg, P., Lou, D., Hjerling-Leffler, J., Haeggström, J., Kharchenko, O., Kharchenko, P.V., et al. (2015). Unbiased classification of sensory neuron types by large-scale single-cell RNA sequencing. Nat. Neurosci. *18*, 145–153.

van Rijn, R.M., Whistler, J.L., and Waldhoer, M. (2010). Opioid-receptor-heteromer-specific trafficking and pharmacology. Curr. Opin. Pharmacol. *10*, 73–79.

Volkow, N.D., and McLellan, A.T. (2016). Opioid abuse in chronic pain–misconceptions and mitigation strategies. N. Engl. J. Med. *374*, 1253–1263.

Vong, L., Ye, C., Yang, Z., Choi, B., Chua, S., Jr., and Lowell, B.B. (2011). Leptin action on GABAergic neurons prevents obesity and reduces inhibitory tone to POMC neurons. Neuron *71*, 142–154.

Wang, W., Loh, H.H., and Law, P.Y. (2003). The intracellular trafficking of opioid receptors directed by carboxyl tail and a di-leucine motif in Neuro2A cells. J. Biol. Chem. *278*, 36848–36858.

Wang, H.B., Zhao, B., Zhong, Y.Q., Li, K.C., Li, Z.Y., Wang, Q., Lu, Y.J., Zhang, Z.N., He, S.Q., Zheng, H.C., et al. (2010). Coexpression of delta- and mu-opioid receptors in nociceptive sensory neurons. Proc. Natl. Acad. Sci. USA *107*, 13117–13122.

Whistler, J.L., Enquist, J., Marley, A., Fong, J., Gladher, F., Tsuruda, P., Murray, S.R., and Von Zastrow, M. (2002). Modulation of postendocytic sorting of G protein-coupled receptors. Science *297*, 615–620.

Williams, J.T., Ingram, S.L., Henderson, G., Chavkin, C., von Zastrow, M., Schulz, S., Koch, T., Evans, C.J., and Christie, M.J. (2013). Regulation of μ-opioid receptors: desensitization, phosphorylation, internalization, and tolerance. Pharmacol. Rev. *65*, 223–254.

Xu, Y., Lopes, C., Wende, H., Guo, Z., Cheng, L., Birchmeier, C., and Ma, Q. (2013). Ontogeny of excitatory spinal neurons processing distinct somatic sensory modalities. J. Neurosci. *33*, 14738–14748.

Yasaka, T., Tiong, S.Y., Hughes, D.I., Riddell, J.S., and Todd, A.J. (2010). Populations of inhibitory and excitatory interneurons in lamina II of the adult rat spinal dorsal horn revealed by a combined electrophysiological and anatomical approach. Pain *151*, 475–488.

Zhu, Y., King, M.A., Schuller, A.G., Nitsche, J.F., Reidl, M., Elde, R.P., Unterwald, E., Pasternak, G.W., and Pintar, J.E. (1999). Retention of supraspinal delta-like analgesia and loss of morphine tolerance in delta opioid receptor knockout mice. Neuron *24*, 243–252.

STAR★METHODS

KEY RESOURCES TABLE

REAGENT or RESOURCE	SOURCE	IDENTIFIER
Antibodies		
Anti-GFP Chicken	Abcam	Cat# ab13970 RRID: AB_300798
Anti-GFP Rabbit	Molecular Probes	Cat# A-11122; RRID: AB_221569
Anti-RFP Rabbit	Abcam	Cat# ab62341; RRID: AB_945213
Anti-NeuN Mouse	Millipore	Cat# MAB377; RRID: AB_2298772
Anti-PKCγ Guinea Pig	Strategic Biosolutions	N/A
Anti-Calbindin Mouse	Sigma-Aldrich	Cat# C9848; RRID: AB_476894
Anti-Calretinin Goat	Swant	Cat# CG1; RRID: AB_10000342
Anti-parvalbumin Goat	Swant	Cat# PVG 214; RRID: AB_10000345
Anti-nNOS Rabbit	Thermo Fisher Scientific	Cat# 61-7000; RRID: AB_2313734
Anti-TLX3 Guinea Pig	Dr. Carmen Birchmeier	N/A
Anti-c-Fos Rabbit	Abcam	Cat# ab7963; RRID: AB_306177
Anti-PAX2 Rabbit	Thermo Fisher Scientific	Cat# 71-6000; RRID: AB_2533990
Anti-MOR Rabbit	Abcam	Cat# ab134054
Anti-CGRP Sheep	Abcam	Cat# ab22560; RRID: AB_725809
Anti-NK1R Rabbit	Novus Biologicals	Cat# NB300-119; RRID: AB_10002802
Anti-ChAT Goat	Millipore	Cat# AB144; RRID: AB_90650
Anti-CD11b Rat	AbD Serotec	Cat# MCA711G; RRID: AB_323167
Anti-Iba-1 Rabbit	Wako	Cat# 019-19741; RRID: AB_839504
Anti-P2Y12 Rabbit	Dr. David Julius	N/A
Anti-WGA Rabbit	Sigma-Aldrich	Cat# T4144; RRID: AB_261669
Chemicals, Peptides, and Recombinant Proteins		
[D-Ala2]-Deltorphin II	Sigma-Aldrich	Cat# T0675
DAMGO	Sigma-Aldrich	Cat# E7384
CTOP	Sigma-Aldrich	Cat# P5296
Naloxone	Sigma-Aldrich	Cat# N7758
Naloxone	Tocris Biosciences	Cat# 0599
SNC80	Tocris Biosciences	Cat# 0764
Fluorogold	Thermo Fisher Scientific	Cat# H22845
IB4 biotinylated	Sigma-Aldrich	Cat# L2140
Lucifer Yellow CH	Thermo Fisher Scientific	Cat# L453
Cholera Toxin Subunit B Alexa Fluor® 555 Conjugate	Thermo Fisher Scientific	C34776
Bicuculline	Tocris Biosciences	Cat# 2503
Strychnine	Sigma-Aldrich	Cat# S0532
CFA	Sigma-Aldrich	Cat# F5881
Critical Commercial Assays		
RNAscope Multiplex Fluorescent Assay	Advanced Cell Diagnostics	Cat# 320850
RNAscope Probe- Mm-Oprd1	Advanced Cell Diagnostics	Cat# 427371
RNAscope Probe- Mm-Oprd1-C2	Advanced Cell Diagnostics	Cat# 427371-C2
RNAscope Probe- Mm-Sst	Advanced Cell Diagnostics	Cat# 404631
Experimental Models: Organisms/Strains		
Mouse: DOR-EGFP	Jackson Laboratories	Stock#:029012; RRID: IMSR_JAX: 029012
Mouse: MOR-mCherry	Jackson Laboratories	Stock#:029013; RRID: IMSR_JAX: 029013
Mouse: DOR KO	Jackson Laboratories	Stock#:007557; RRID: IMSR_JAX: 007557

(*Continued on next page*)

Continued

REAGENT or RESOURCE	SOURCE	IDENTIFIER
Mouse: MOR KO	Jackson Laboratories	Stock#: 007559; RRID: IMSR_JAX: 007559
Mouse: DOR fl	Jackson Laboratories	Stock#: 030075; RRID: IMSR_JAX: 030075
Mouse: Vgat-IRES-Cre	Jackson Laboratories	Stock# 016962; RRID: IMSR_JAX:016962
Mouse: Vglut2-IRES-Cre	Jackson Laboratories	Stock# 016963; RRID: IMSR_JAX:016963
Mouse: Sst-IRES-Cre	Jackson Laboratories	Stock# 013044; RRID: IMSR_JAX: 013044
Mouse: *En1*-IRES-Cre	Dr. Tom Jessell	Kimmel et al., 2000
Mouse: Ai 14 (RCL-tdT)	Jackson Laboratories	Stock# 007914; RRID: IMSR_JAX:007914
Mouse: Ai 6 (RCL-ZsGreen)	Jackson Laboratories	Stock# 007906; RRID: IMSR_JAX:007906
Mouse: Penk-IRES-Cre	Dr. Adam Hantman	This paper
Sequence-Based Reagents		
flox EX FW (5′- GCAATCACACCTTGGCCATT-3′)	This paper	N/A
flox EX RV (5′- CCGATTGGGTCATTCAGGGA-3′)	This paper	N/A
Recombinant DNA		
AAV2-CBA-FLEx-WGA	Dr. Reza Sharif-Naeini	Petitjean et al., 2015
AAV-DJ-ef1a-DIO-eYFP	Stanford University Gene Vector and Virus Core	N/A
Software and Algorithms		
pClamp10	Molecular devices	N/A
Clampfit 10.3	Molecular devices	N/A
Prism7	Graphpad	N/A
Photoshop CS6	Adobe	N/A
Illustrator CS6	Adobe	N/A
Excel 2010	Microsoft	N/A
Igor Pro 6	WaveMetrics	N/A

CONTACT FOR REAGENT AND RESOURCE SHARING

Further information and requests for reagents may be directed to, and will be fulfilled by, the Lead Contact and corresponding author, Dr. Grégory Scherrer (gs25@stanford.edu).

EXPERIMENTAL MODEL AND SUBJECT DETAILS

All procedures followed animal care guidelines approved by Stanford University's Administrative Panel on Laboratory Animal Care (APLAC) and the recommendations of the International Association for the Study of Pain. Mice were housed 2-5 per cage and maintained on a 12 hr light/dark cycle in a temperature controlled environment with ad lib access to food and water. We typically use male mice with the age range from 3 to 8 weeks old for our experiments. The generation and characterization of *Oprd1*$^{egfp/egfp}$ mice (Scherrer et al., 2006), *Oprm1*$^{mCherry/mCherry}$ mice (Erbs et al., 2015), *Oprd1* knockout mice (Filliol et al., 2000), *Oprm1* knockout mice (Matthes et al., 1996), *Oprd1*$^{flox/flox}$ mice (Gaveriaux-Ruff et al., 2011), *Vglut2*^{Cre} and *Vgat*^{Cre} mice (Vong et al., 2011), *Som*^{Cre} mice (Taniguchi et al., 2011), *Engrailed1*^{Cre} mice (Kimmel et al., 2000), *Penk*^{Cre} mice (Francois et al., 2017), *Cre-inducible ROSA26*^{tdTomato} and *ROSA26*^{Zsgreen} reporter mice (Madisen et al., 2010) have been described previously. C57BL/6 wild-type mice were purchased from Jackson lab.

METHOD DETAILS

Drugs Administration

The following chemicals were used in this study: [D-Ala2]-Deltorphin II (Sigma T0675), [D-Ala2, N-Me-Phe4, Gly5-ol]-Enkephalin acetate salt (DAMGO, Sigma E7384), CTOP (Sigma P5296), Naloxone (Sigma N7758), CYM51010 (Sigma SML0980), SNC80 (Tocris Cat. No. 0764), Fluorogold (Thermo Fisher Scientific, H22845), Lucifer Yellow CH (Thermo Fisher Scientific, L453), Cholera Toxin Subunit B (Recombinant) Alexa Fluor 555 Conjugate (Thermo Fisher Scientific, C34776). For inducing DOR internalization, SNC80 (10 mg/kg) was injected subcutaneously to lightly restrained unanesthetized mice using a 30 G needle attached to a microsyringe

inserted through the back skin. For behavioral experiments, deltorphin II (1 μg), CTOP (100 ng) or a vehicle solution (0.9% sodium chloride, Hospira NDC 0409-4888-10) were injected intrathecally. A 30 G needle attached to a microsyringe was inserted between the L4/L5 vertebrae, puncturing through the dura (confirmed by the presence of a reflexive tail flick), and then 5 μl of drug was injected.

Immunohistochemistry

We employed an immunostaining protocol described previously (Bardoni et al., 2014; Scherrer et al., 2009). Briefly, 5 to 8 week old mice were deeply anesthetized with ketamine-xylazine and perfused transcardially with 0.1 M phosphate-buffered saline (PBS) followed by 4% formaldehyde solution (Sigma F1635) in 0.1 M phosphate buffer (PB). The spinal cord was dissected, post-fixed for 4 hr at 4°C, and cryoprotected overnight in 30% sucrose in PBS. Frozen spinal cord tissue was then cut at 40 μm and incubated with a 5% NDST blocking solution (0.3% Triton X-100 sloution in 0.1 M PBS plus with 5% normal donkey serum) for at least 1 hr. The primary antibody was diluted in the same solution, and incubated with spinal cord sections overnight at 4°C. After washing the primary antibody 3 times for 5 min with 0.3% Triton X-100 solution in 0.1 M PBS, sections were incubated with secondary antibody solution in 1% NDST solution at room temperature for 2 hr. Sections were then mounted in the glass slide with Fluoromount (Southern Biotech) after washing with PB for 3 times for 5 min. Images were acquired with a confocal microscope (Leica DM2500). We used the following primary antibodies: anti-GFP: Abcam (chicken; 1:1000); Molecular Probes (rabbit; 1:1000), anti-RFP: Abcam (Rabbit; 1:1000); anti-NeuN: EMD millipore (mouse; 1:1000); anti-PKCγ: Strategic Biosolutions (guinea pig, 1:10000); anti-Calbindin: Sigma (Mouse; 1:1000); anti-Calretinin: Swant (Goat; 1:2000); anti-parvalbumin: Swant (Goat; 1:5000); anti-NOS: Thermo Fisher Scientific (Rabbit; 1:1000); anti-TLX3: Carmen Birchmeier (guinea pig; 1:10000); anti-c Fos: Abcam (rabbit; 1:500); anti-PAX2: Thermo Fisher Scientific (rabbit; 1:50); anti-MOR: Abcam (rabbit; 1:500); anti-CGRP: Abcam (sheep; 1:2000), anti-NK1R: Novus Biologicals (Rabbit; 1:1000); anti-ChAT: EMD Millipore (Goat; 1:100); anti-CD11b: AbD Serotec (Rat:1:1000); anti-Iba-1: Wako (Rabbit; 1:1000); Anti-WGA: Sigma (Rabbit,1:1000) anti-P2Y12: gift from Dr. David Julius lab (Rabbit; 1:1000). To identify IB4-binding cells, biotinylated IB4 (Sigma, 1:500) and fluorophore conjugated streptavidin (Molecular Probes, 1:1000) were used in place of primary and secondary antibodies.

In Situ Hybridization

We used Panomics' QuantiGene ViewRNA (Affymetrix) and Advanced Cell Diagnostics RNAscope Technology (ACD Bioscience). Briefly, wild-type mice with 5 to 8 week old were deeply anesthetized with ketamine-xylazine and perfused transcardially with 0.1 M PBS followed by 4% formaldehyde solution in PB. Spinal cord or DRG were dissected, cryoprotected in 30% sucrose overnight and then frozen in OCT. Frozen tissue was cut at 14 μm onto Superfrost Plus slides and kept at −80°C. Tissue was thawed from −80°C, washed with PBS at room temperature (RNAscope) or placed directly into 10% formalin for 10 min (QuantiGene ViewRNA), and subsequently processed according to the manufacturer's protocol. For QuantiGene ViewRNA, we treated the tissue with protease for an optimal time of 12 min and then incubated with the RNA probe set for 3 hr at 40°C. For RNAscope, we first pretreated the tissue with solutions from the pretreatment kit to permeabilize the tissue, and then incubated with protease for 30 min and the hybridization probe(s) for another 2 hr at 40°C.

Subcellular Fluorescence Density

The quantification of subcellular fluorescence density has been described previously (Scherrer et al., 2006). DORGFP or MOR IHC fluorescence intensity values per surface unit (pixel) were determined using ImageJ after subtracting the nuclear background fluorescence (fluorescence density, Df). Ratios of surface (Df surf) versus cytoplasmic (Df cyto) fluorescence densities were then calculated. A ratio value of 1.0 indicates equal DORGFP or MOR IHC fluorescence densities at the cell surface and in the cytoplasm.

Noxious Mechanical and Thermal Stimulation

In order to induce Fos expression in spinal cord dorsal horn neurons, the hindpaw of anesthetized DORGFP;MORmCherry mice were either clamped with a surgical forceps (mechanical) or immersed in a 55°C water bath (thermal) for 30 s. Treatments were repeated 3 times with a 60 s interval. SNC80 was injected subcutaneously 60 min after noxious stimulation to trigger DOR internalization.

Genomic DNA Analysis

We used PCR on genomic DNA to detect exon 2 deletion in spinal cord but not DGR from *Som* ^{Cre};*Oprd1*$^{fl/fl}$ mice (Figure S3C). To confirm the selective ablation of DOR in the spinal cord somatostatin+ neurons, we designed two primers located outside of the LoxP site for detecting the excised band. The forward primer (5′- GCAATCACACCTTGGCCATT −3′) and reverse primer (5′- CCGATTGGGTCATTCAGGGA −3′) resulted in a 353bp DNA fragment that was seen in spinal cord but absent in DRG. Littermate mice with the genotype *Som*^{Cre}*; Oprd1*$^{+/+}$ used as a control subject.

Retrograde Tracing

Five to 8 week old DORGFP;MORmCherry or wild-type mice were anaesthetized with isofluorane and placed on a stereotaxic surgical device (David Kopf Instruments). Three hundred nanoliters of fluorogold (FG, 1 mg/mL) or the B fragment of cholera toxin Alexa fluor 555 (CTB 555, 0.5 mg/mL) were injected unilaterally with a glass micro-pipette into the lateral parabrachial nucleus (antero-posterior, −5.0 mm; medio-lateral, 1.4 mm; dorso-ventral, 3.5 mm) or cerebellum (antero-posterior, −6.95 mm; medio-lateral, 1.75 mm;

dorso-ventral, 2.5 mm) at a rate of ~60 nL/min. 5-7 days following injection, spinal cord and brain tissue was collected for histological or electrophysiological analysis.

For spinal injections of Cre dependent WGA and YFP virus, thoracic vertebra L4 was exposed by carefully removing the paraspinal muscles. The animal was then placed into a stereotaxic frame and vertebrae were immobilized using a pair of spinal adaptors. The T12 dorsal spinous process was removed to expose the dura mater and lumbar spinal cord. Viral vectors (mixed with a ratio of 2 YFP-expressing vector for 1 WGA-expressing vector) were injected to the right of the posterior median spinal vein at a depth of 300 μm. Pulled glass micropipettes were used to inject 250 nL of vector solution at a speed of 40 nL/min. Spinal cord tissue was collected for histological 4 weeks after injection.

Spinal Cord Slice Preparation and Electrophysiology

Three to 8 week old mice were anesthetized with isoflurane, decapitated, and the vertebral column was rapidly removed and placed in oxygenated ice-cold dissection solution (in mM: 95 NaCl, 2.5 KCl, 1.25 $NaH2PO_4$, 26 $NaHCO_3$, 50 sucrose, 25 glucose, 6 $MgCl_2$, 1.5 $CaCl_2$, and 1 kynurenic acid, pH 7.4, 320 mOsm). The lumbar spinal cord was isolated, embedded in a 3% agarose block and transverse slices (400 μm thick) with dorsal roots attached were made using a vibrating microtome (Leica VT1200S). Slices were incubated in oxygenated recovery solution (in mM: 125 NaCl, 2.5 KCl, 1.25 NaH_2PO4, 26 $NaHCO_3$, 25 glucose, 6 $MgCl_2$, and 1.5 $CaCl_2$, pH 7.4, 320 mOsm) at 35°C for 1 hr. Patch-clamp recording in whole-cell configuration was performed at RT on laminae I-II neurons visualized with an Olympus BX51WI microscope fitted with a QIClick QImaging camera. Slices were perfused at ~2 mL/min with recording solution (recovery solution containing 1 mM $MgCl_2$ and 2 mM $CaCl_2$). Recordings were performed in voltage-clamp mode at a holding potential of −70 mV. Thick-walled borosilicate pipettes, having a resistance of 3-6 MOhm, were filled with internal solution (in mM: 120 K-methyl-sulfonate, 10 NaCl, 10 EGTA, 1 $CaCl_2$, 10 HEPES, 0.5 NaGTP, 5 MgATP, pH adjusted to 7.2 with KOH, osmolarity adjusted to 305 with sucrose). Data were acquired using a Multiclamp 700B amplifier and pClamp10 software (Molecular Devices, USA). Sampling rate was 10 kHz and data were filtered at 2 kHz. We monitored opioid receptor-mediated GIRK currents as a read out to determine whether a neuron expresses DOR or MOR, following bath perfusion of the DOR agonist deltorphin II (1 μM) or MOR agonist DAMGO (1 μM). For dorsal root stimulation experiments, the dorsal root was stimulated using a DS4 Bi-phasic current stimulator (Digitimer, UK), every 30 s for 0.2 ms with an intensity of 20 μA, 100 μA or 500 μA for Aβ, Aδ and C fibers, respectively (Torsney and MacDermott, 2006). Aβ, Aδ or C fiber responses were characterized as monosynaptic based on the constant conduction latency and absence of failure during a train of stimuli at 20, 10 or 1 Hz frequency, respectively. Analysis of eEPSC peak amplitudes was done with Clampfit software (pClamp9, Molecular Devices, USA). Graphs and statistical analysis were generated with Igor Pro and Microsoft Excel.

Behavioral Tests

For all behavior sensory assays described below, mice were acclimated to the testing environment for at least 30 min within custom-made red plastic cylinders (10.16 cm D) on a raised perforated metal platform (60.96 cm H). Baseline testing was conducted 30 min prior to drug injections, and drug effects were assessed at 30 min post administration.

To induce inflammation pain, 10 μL of undiluted-CFA solution (Complete Freund’s Adjuvant, Sigma F5881) was injected into the glabrous skin of left hindpaw by a 30G needle. Behavioral assays were performed 72 hr after injection.

To induce a chronic pain state, we utilized a modified version of the Spared Nerve Injury (SNI) model of neuropathic pain. This robust and reliable model entails surgical damage to two of the sciatic nerve branches (common peroneal and tibial branches) while sparing the third (sural branch). Following the SNI procedure, the receptive field of the lateral aspect of the hindpaw (innervated by the sural nerve) displays a hypersensitive phenotype. To perform this peripheral nerve injury procedure, anesthesia was induced and maintained throughout surgery with isoflurane (4% induction, 1.5% maintenance in oxygen). The left hind-leg was shaved and wiped clean with alcohol and betadine. A 1 cm incision was made in the skin of the upper thigh, approximately where the sciatic nerve trifurcates. The overlying biceps femoris muscles were retracted by blunt dissection, exposing the common peroneal, tibial, and sural branches of the sciatic nerve. Next, 2 mm of both the common peroneal and tibial nerves were transected and removed, with care not to distend the sural nerve. The muscle was then sutured with 6–0 sutures, and the skin closed with tissue adhesive (3M Vetbond), followed by a Betadine application. During recovery from surgery, mice were placed under a heat lamp until awake and achieved normal balanced movement. Mice were then returned to their home cage and closely monitored over the following three days for well-being or the spared nerve injury (SNI) induced neuropathic pain animals, behavioral assays were performed 28 days after the SNI surgery.

Von Frey withdrawal threshold test

A set of 8 von Frey filaments (Stoelting, Illinois), ranging from 0.007 to 6.0 g were used to assess mechanical withdrawal thresholds. Filaments were applied perpendicular to the ventral-medial hindpaw surface with sufficient force to cause a slight bending of the filament. A positive response was characterized by a rapid withdrawal of the paw away from the stimulus fiber within 4 s. The Up-Down method was used to determine the mechanical threshold (50% withdrawal threshold) (Chaplan et al., 1994).

Von Frey withdrawal frequency test

To evaluate mechanical sensitivity we used six von Frey filaments (0.07 g, 0.16 g, 0.4 g, 1.0 g and 1.4 g and 6 g for normal mice; 0.008 g,0.02 g,0.07 g,0.16 g,0.4 g,1 g for SNI pain model mice). Filaments were applied perpendicular to the ventral-medial hindpaw surface with sufficient force to cause a slight bending of the filament. Each filament was applied for one second. A positive response

was characterized by a rapid and immediate withdrawal of the paw away from the stimulus fiber. Each filament was applied a total of 5 times, and the frequency of reflexive withdrawal responses was calculated.

Pinprick withdrawal frequency test

We gently touched the plantar surface of the hindpaw with a blunted 25G needle, and measured numbers of withdrawal response per 5 tries with 1 min intervals.

Hot plate test

Mice were acclimated to the testing environment as described above. The plate temperature was set to 52.5 °C. The mouse was placed on the plate and the latency to lick and/or bite a hindpaw was scored. A cut-off of 20 s was set to prevent tissue damage.

Tail immersion test

The mouse was gently restrained and 2 cm of the tip of the tail was submerged in the 50 °C water bath, and the latency to reflexively withdrawal the tail from the water was recorded as a positive nociceptive reflex response. A maximal cut-off of 20 s was set to prevent tissue damage. Only one tail immersion was applied on a given testing session, as to prevent behavioral sensitization that can result from multiple noxious immersions.

QUANTIFICATION AND STATISTICAL ANALYSIS

All statistical analysis was performed with Igor Pro, GraphPad prism 7 or Microsoft Excel software. No assumption was made regarding the distribution of the data. Statistical comparison was performed using Student's t test, one-way ANOVA or repeated-measures ANOVA and Bonferroni-Dunn post hoc test. Quantification data are presented as mean ± SEM with dots showing individual data points and $p < 0.05$ was considered statistically significant. Except when specifically indicated, the numbers of replications (n) represent the total number of animals used in immunohistochemical or behavioral experiments, and the number of neurons recorded in electrophysiological experiments.

Neuron, Volume *98*

Supplemental Information

Functional Divergence of Delta and Mu Opioid Receptor Organization in CNS Pain Circuits

Dong Wang, Vivianne L. Tawfik, Gregory Corder, Sarah A. Low, Amaury François, Allan I. Basbaum, and Grégory Scherrer

SUPPLEMENTARY FIGURES AND FIGURE LEGENDS

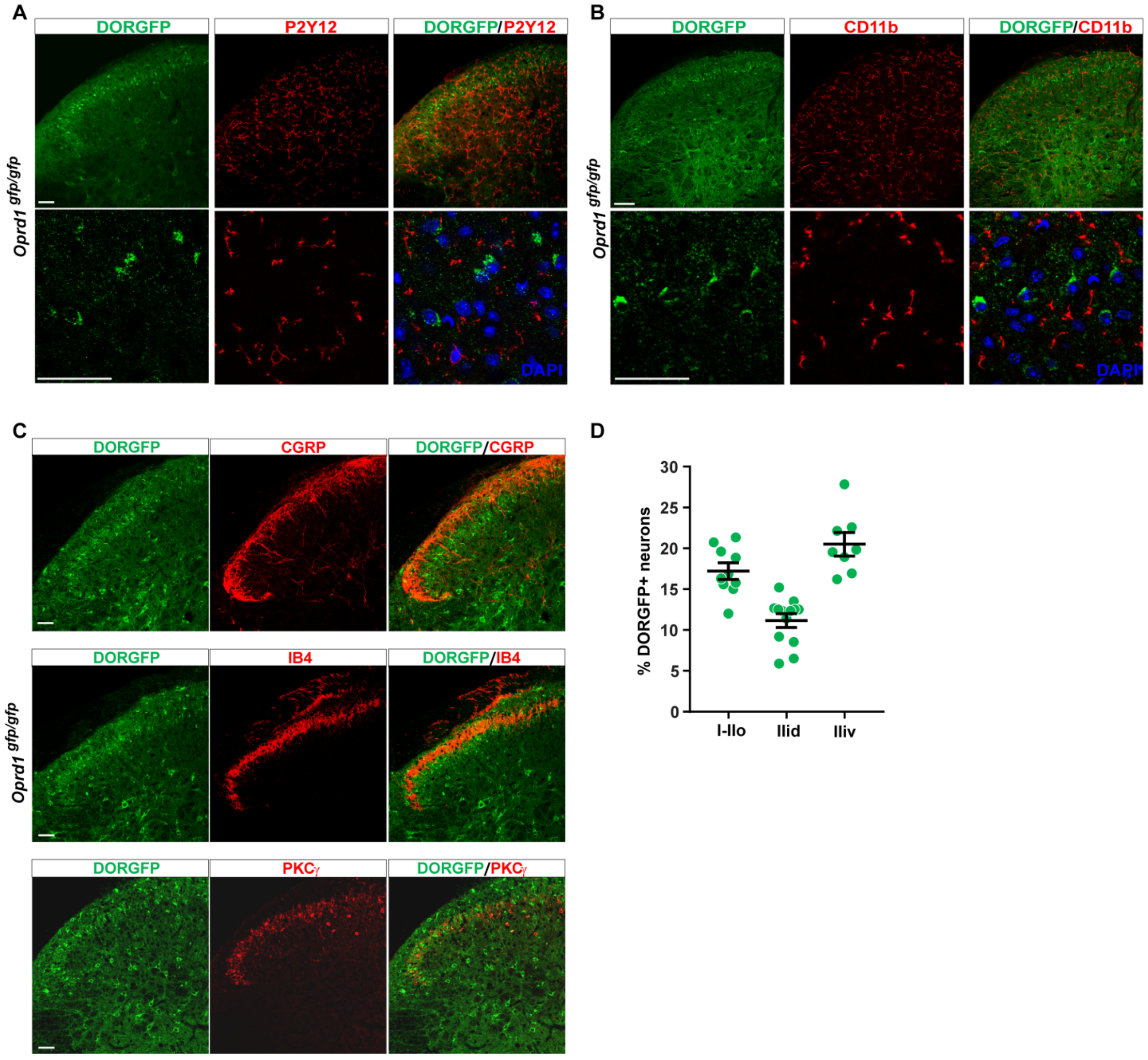

Figure S1

Figure S1. DORGFP Mouse Reports DOR Expression in Spinal Cord.

(Related to Figure 1)

(A-B) DOR is absent in spinal cord astrocytes and microglia by co-staining with anti-GFP and -P2Y12 or -CD11b antibodies.

(C) DORGFP+ neuron distribution in the dorsal horn.

Upper: Double labeling of GFP and CGRP in sections from DORGFP mice shows that 17.2% of DOR+ neurons are located in lamina I and II outer.

Middle: 11.2% of DOR+ neurons are located in lamina II inner dorsal within the IB4+ band.

Bottom: 20.5% of DOR+ neurons are located in lamina II inner ventral within the PKCγ+ band.

(D) Quantification of (C). Data are presented as mean ± SEM with dots showing individual counts in spinal cord sections from 3 mice.

Scale bars represent 50 μM.

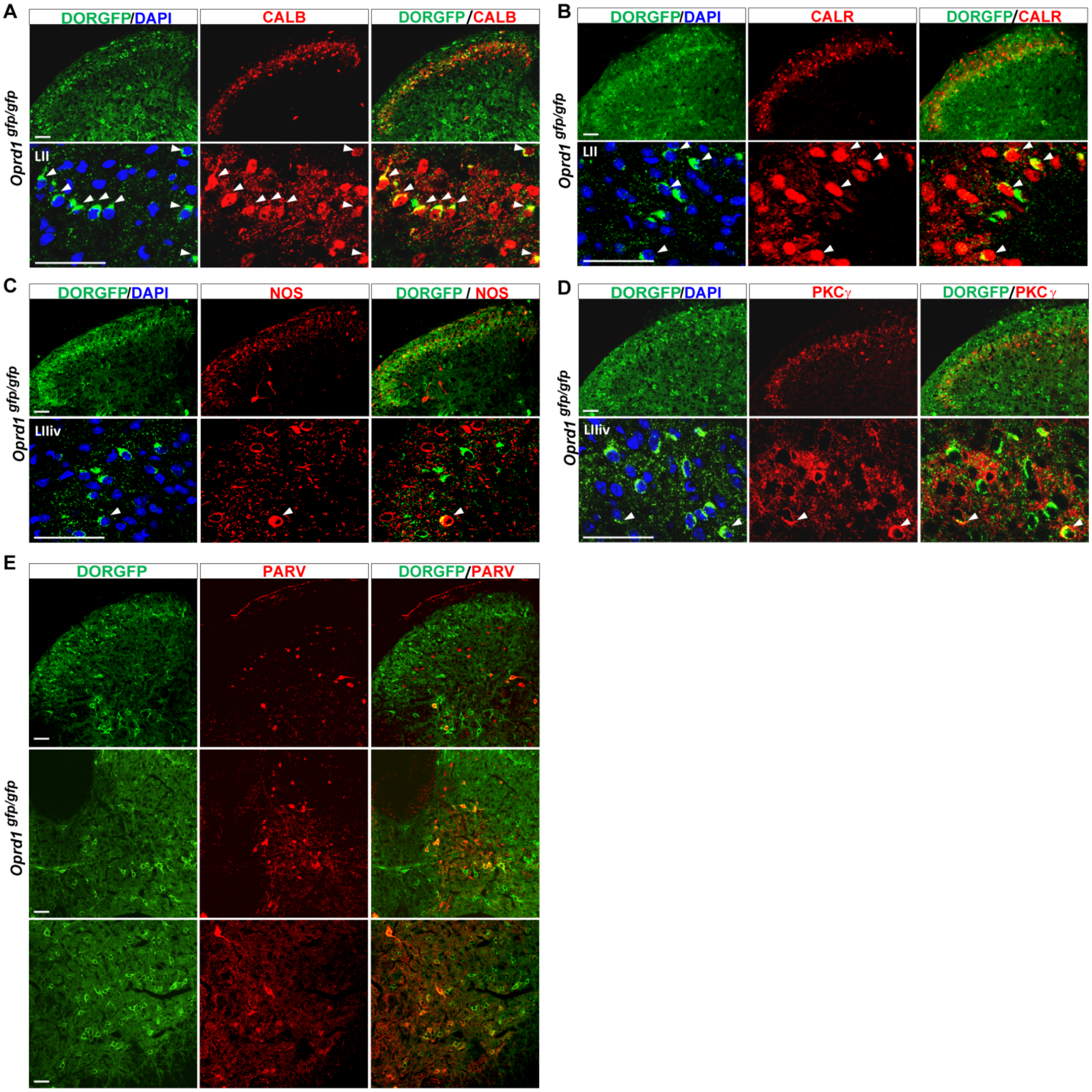
A
DORGFP/DAPI
CALB
DORGFP/CALB
LII
B
DORGFP/DAPI
CALR
DORGFP/CALR
LII
C
DORGFP/DAPI
NOS
DORGFP / NOS
LIIiv
D
DORGFP/DAPI
PKCγ
DORGFP/PKCγ
LIIiv
E
DORGFP
PARV
DORGFP/PARV
Oprd1 gfp/gfp

Figure S2

Figure S2. DORGFP+ Neurons in the Dorsal Horn Express Markers of Excitatory Interneurons.

(Related to Figure 2)

(A) CALB is expressed by 50.7% of DORGFP+ neurons. White arrowheads indicate cells where co-staining occurs.

(B) CALR is present in 26.5% of DORGFP+ neurons. White arrowheads indicate DORGFP+ CALR+ neurons.

(C) NOS is expressed by 12.1% of DORGFP+ neurons. White arrowheads indicate neurons that co-express DORGFP and NOS.

(D) PKCγ is present in 6.9% of DORGFP+ neurons. White arrowheads show DORGFP+ PKCγ+ neurons.

(E) DOR is absent in dorsal horn lamina III PARV+ neurons (top row) but is expressed by PARV+ neurons in the deep DH laminae IV-V (middle row) and by PARV+ neurons in the ventral horn (bottom row).

Scale bars represent 50 μM.

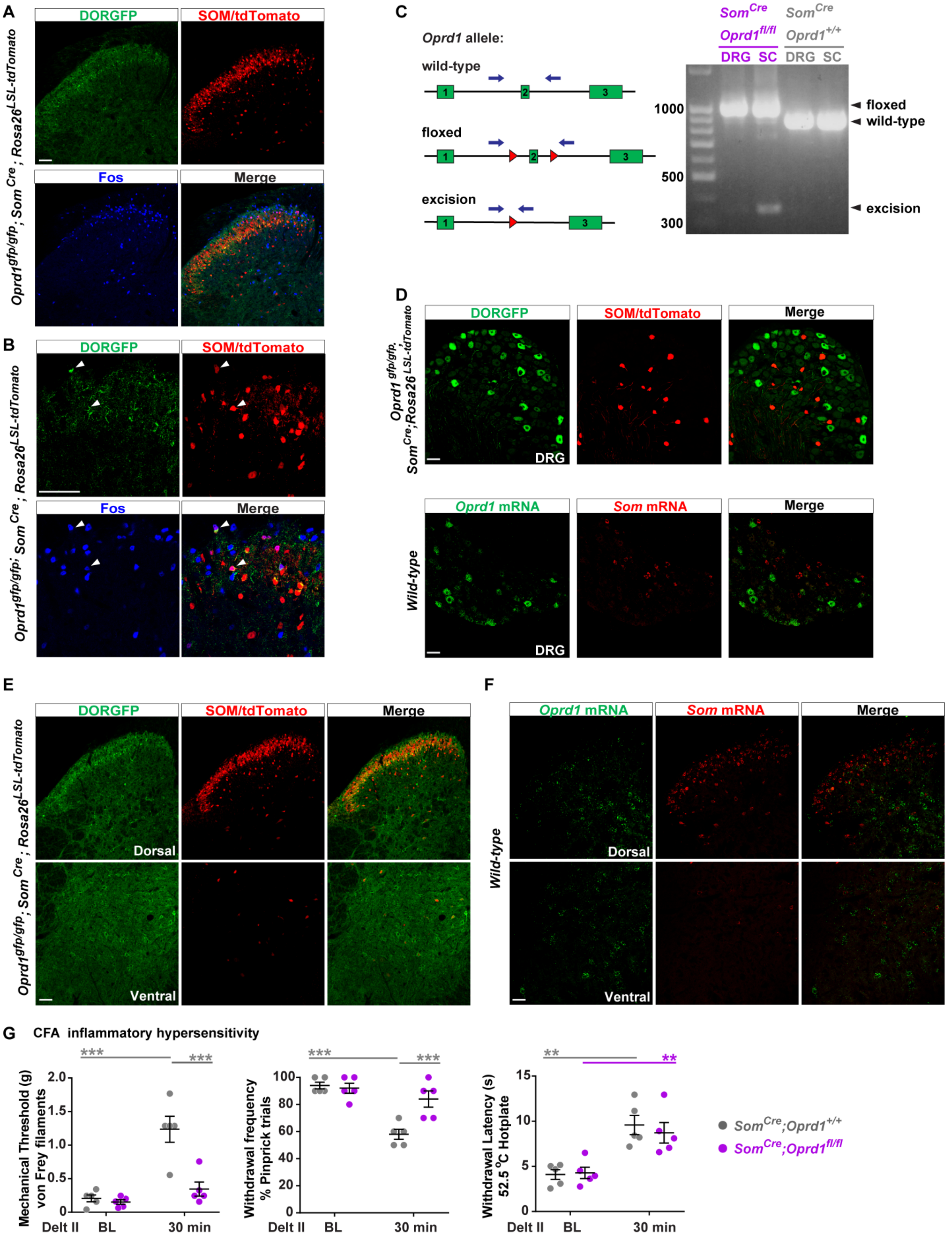
A
DORGFP
SOM/tdTomato
Fos
Merge
Oprd1gfp/gfp; SomCre; Rosa26LSL-tdTomato
B
DORGFP
SOM/tdTomato
Fos
Merge
Oprd1gfp/gfp; SomCre; Rosa26LSL-tdTomato
C
Oprd1 allele:
wild-type
floxed
excision
SomCre Oprd1fl/fl
SomCre Oprd1+/+
DRG SC DRG SC
1000
500
300
floxed
wild-type
excision
D
DORGFP
SOM/tdTomato
Merge
Oprd1gfp/gfp; SomCre;Rosa26LSL-tdTomato
DRG
Oprd1 mRNA
Som mRNA
Merge
Wild-type
DRG
E
DORGFP
SOM/tdTomato
Merge
Oprd1gfp/gfp; SomCre; Rosa26LSL-tdTomato
Dorsal
Ventral
F
Oprd1 mRNA
Som mRNA
Merge
Wild-type
Dorsal
Ventral
G
CFA inflammatory hypersensitivity
Mechanical Threshold (g) von Frey filaments
Withdrawal frequency % Pinprick trials
Withdrawal Latency (s) 52.5 °C Hotplate
Delt II BL 30 min
SomCre;Oprd1+/+
SomCre;Oprd1fl/fl

Figure S3

Figure S3. DOR in Spinal SOM+ Neurons Specifically Regulates Mechanical Pain. (Related to Figure 3)

(A) Noxious mechanical stimulation of the hindpaw of *Oprd1*$^{gfp/gfp}$*;Som*^{Cre}*;Rosa26*$^{LSL\text{-}tdTomato}$ mice mice induced Fos expression in some DORGFP+ SOM+ dorsal horn neurons.

(B) High magnification images from (A). White arrowheads indicate DORGFP+ SOM+ neurons expressing Fos.

(C) PCR showing the recombination of the floxed allele and selective deletion of *Oprd1* in the spinal cord, but not DRG of *Som*^{Cre}; *Oprd1*$^{fl/fl}$ mice .

(D) Double labeling of GFP and tdTomato in sections from *Oprd1*$^{gfp/gfp}$; *Som*^{Cre}; *Rosa26*$^{LSL\text{-}tdTomato}$ mice shows that DOR is absent from SOM+ neurons in DRG neurons. Bottom: Fluorescence in situ hybridization experiments in wild-type mice confirm that *Oprd1* mRNA is absent from SOM+ DRG neurons.

(E) SOM is rarely present in spinal cord ventral horn neurons.

(F) Fluorescence in situ hybridization experiments in wild-type mice confirm that *Som* mRNA is rarely present in the spinal cord ventral horn.

(G) Decreased effect of intrathecal deltorphin II (15 μg) against CFA-induced inflammatory mechanical but not thermal hypersensitivity in DOR cKO mice. Data are presented as mean ± SEM . **, $P < 0.01$, ***, $P < 0.001$, repeated measures, Two-way ANOVA + Bonferroni. Von Frey threshold, pinprick and hotplate tests: n=5 control mice and 5 DOR cKO mice.

Scale bars represent 50 μM.

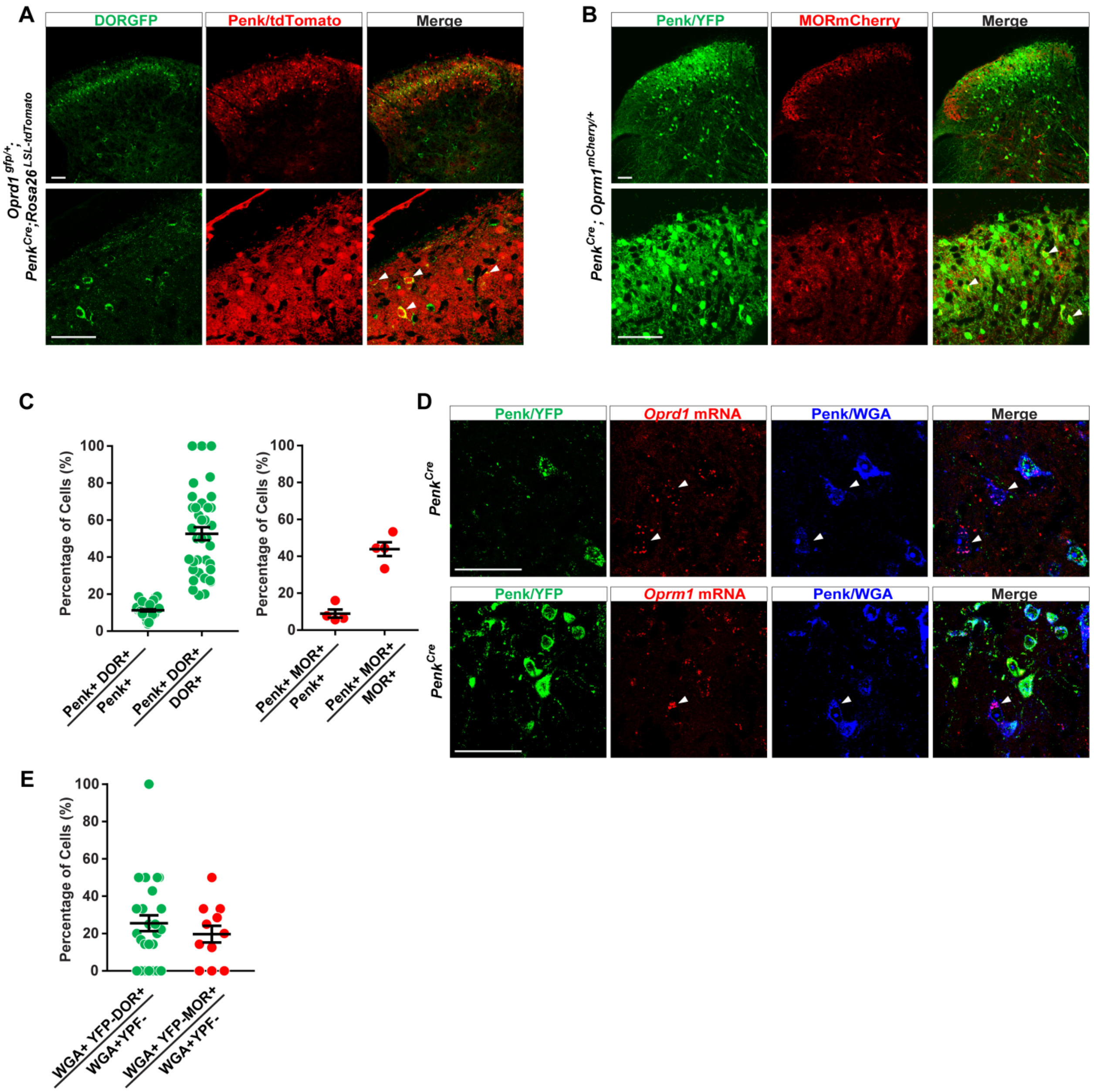
A
DORGFP
Penk/tdTomato
Merge
Oprd1 gfp/+; PenkCre;Rosa26 LSL-tdTomato
B
Penk/YFP
MORmCherry
Merge
PenkCre; Oprm1mCherry/+
C
Percentage of Cells (%)
100
80
60
40
20
0
Penk+ DOR+ / Penk+
Penk+ DOR+ / DOR+
Penk+ MOR+ / Penk+
Penk+ MOR+ / MOR+
D
Penk/YFP
Oprd1 mRNA
Penk/WGA
Merge
PenkCre
Oprm1 mRNA
E
WGA+ YFP-DOR+ / WGA+YPF-
WGA+ YFP-MOR+ / WGA+YPF-

Figure S4

Figure S4. Spinal Neurons Expressing DOR or MOR Receive Synaptic Input from Enkephalinergic Neurons.

(Related to Figure 3 and Figure 5)

(A) Double labeling of GFP and tdTomato in sections from *Oprd1*$^{gfp/+}$;*Penk*^{Cre};*Rosa26*$^{LSL\text{-}tdTomato}$ mice shows that DOR is expressed by the Penk+ spinal dorsal horn neurons. White arrowheads indicate DORGFP+ Penk+ neurons.

(B) Co-staining of YFP and mCherry in sections from *Penk*^{Cre};*Oprm1*$^{mCherry+}$ mice shows that MOR is expressed by the Penk+ spinal dorsal horn neurons. White arrowheads indicate MORmCherry+ Penk+ neurons.

(C) Quantification of (A) and (B).

(D) Wheat germ agglutinin (WGA) anterograde tracing from Penk+ neurons and fluorescent in situ hybridization experiments in *Penk*^{Cre} mice indicates both DOR+ (upper panel) and MOR+ neurons (bottom panel) receive synaptic input from Penk+ spinal neurons.

(E) Quantification of (D).

Data are presented as mean ± SEM in (C) and (E). Scale bars represent 50 μM.

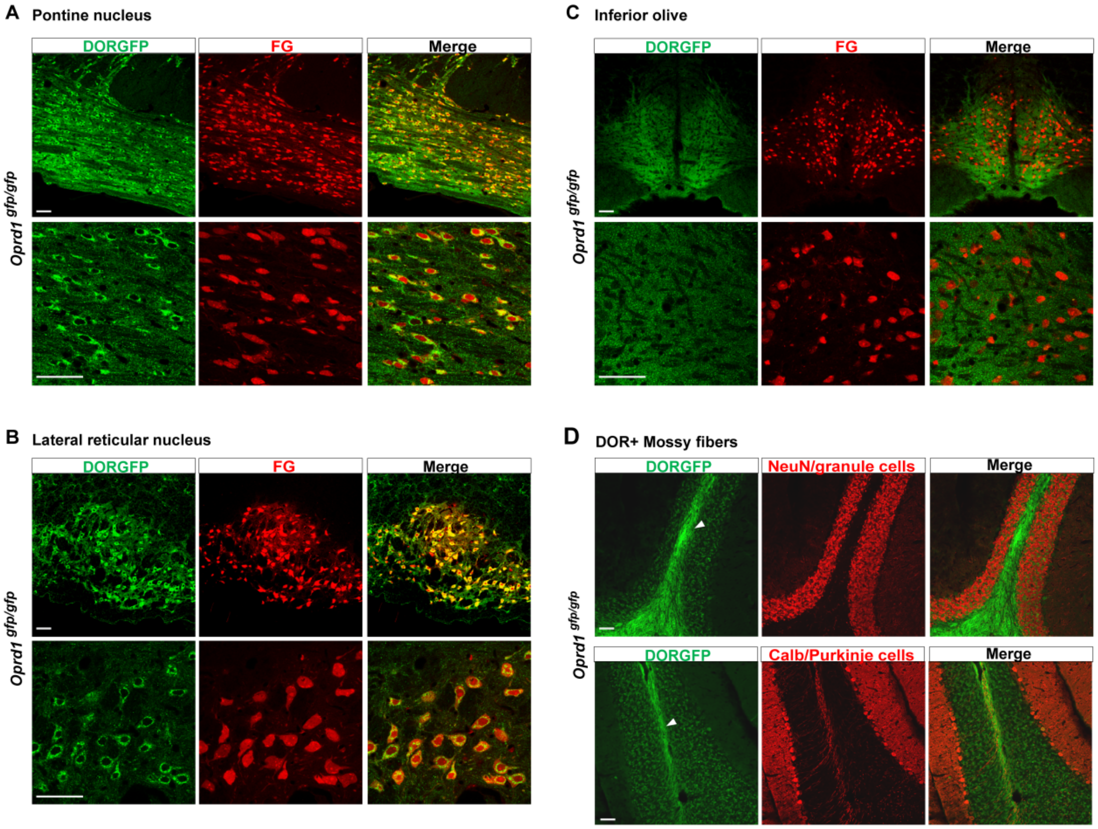
A
Pontine nucleus
DORGFP
FG
Merge
Oprd1 gfp/gfp
B
Lateral reticular nucleus
DORGFP
FG
Merge
Oprd1 gfp/gfp
C
Inferior olive
DORGFP
FG
Merge
Oprd1 gfp/gfp
D
DOR+ Mossy fibers
DORGFP
NeuN/granule cells
Merge
DORGFP
Calb/Purkinje cells
Merge
Oprd1 gfp/gfp

Figure S5

Figure S5. DOR Expression in Neurons that Project to the Cerebellum.

(Related to Figure 4)

(A) DOR is expressed by pontine nucleus neurons that project to the cerebellum, identified by fluorogold (FG) injection in cerebellum.

(B) Expression of DOR in lateral reticular nucleus neurons projecting to the cerebellum.

(C) DOR is absent in the inferior olive neurons that project to the cerebellum.

(D) DOR is present in mossy fibers (white arrowhead) innervating cerebellar granule cells, consistent with DOR expression in pre-cerebellar nuclei.

Scale bars represent 50 μM.

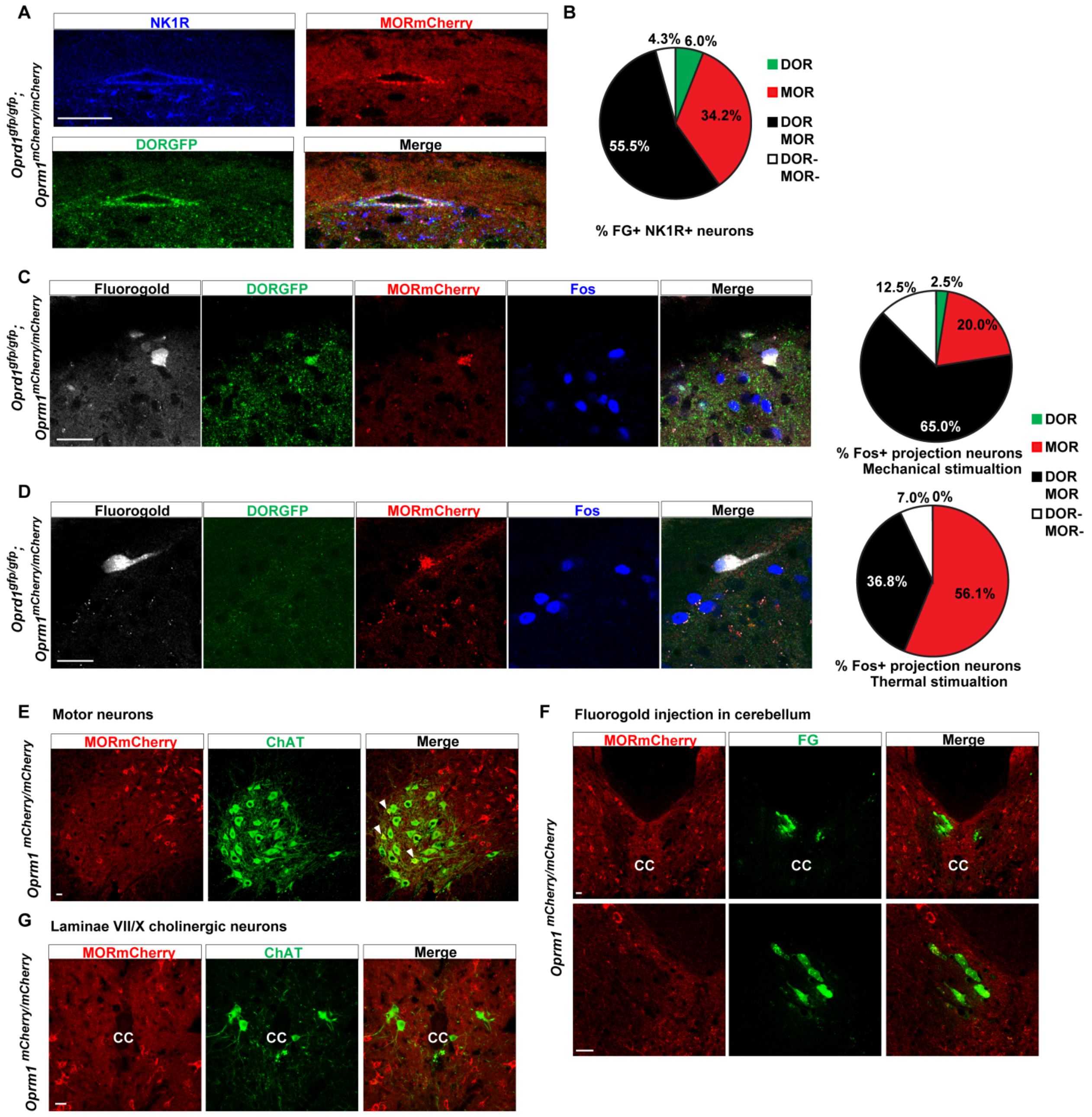
A
NK1R
MORmCherry
Oprd1gfp/gfp; Oprm1mCherry/mCherry
DORGFP
Merge
B
4.3%
6.0%
34.2%
55.5%
DOR
MOR
DOR MOR
DOR- MOR-
% FG+ NK1R+ neurons
C
Fluorogold
DORGFP
MORmCherry
Fos
Merge
12.5%
2.5%
20.0%
65.0%
% Fos+ projection neurons Mechanical stimualtion
D
7.0%
0%
36.8%
56.1%
% Fos+ projection neurons Thermal stimualtion
E
Motor neurons
Oprm1 mCherry/mCherry
ChAT
F
Fluorogold injection in cerebellum
FG
CC
G
Laminae VII/X cholinergic neurons

Figure S6

Figure S6. DOR and MOR are Co-expressed in Lamina I NK1R+ Projection Neurons but not in Motor Neurons.

(Related to Figure 6)

(A-B) DOR and MOR are co-expressed by 55.5% of lamina I NK1R+ projections (n=117 neurons from 3 mice).

(C) Fos is expressed by 65.0% of DOR+MOR+ projection neurons following mechanical stimulation of the hindpaw of DORGFP;MORmCherry mice.

(D) Noxious heat stimulation induced Fos expression in 92.8% of MORmCherry+ projection neurons.

(E) Double labeling of RFP and ChAT in spinal cord sections from MORmCherry mice shows that MOR is expressed by few ChAT+ motor neurons with small diameter cell bodies.

(F) MOR is largely absent from Clarke's column neurons. CC, central canal.

(G) MOR is rarely expressed by cholinergic partition cells and central canal cluster neurons.

Scale bars represent 20 μM.

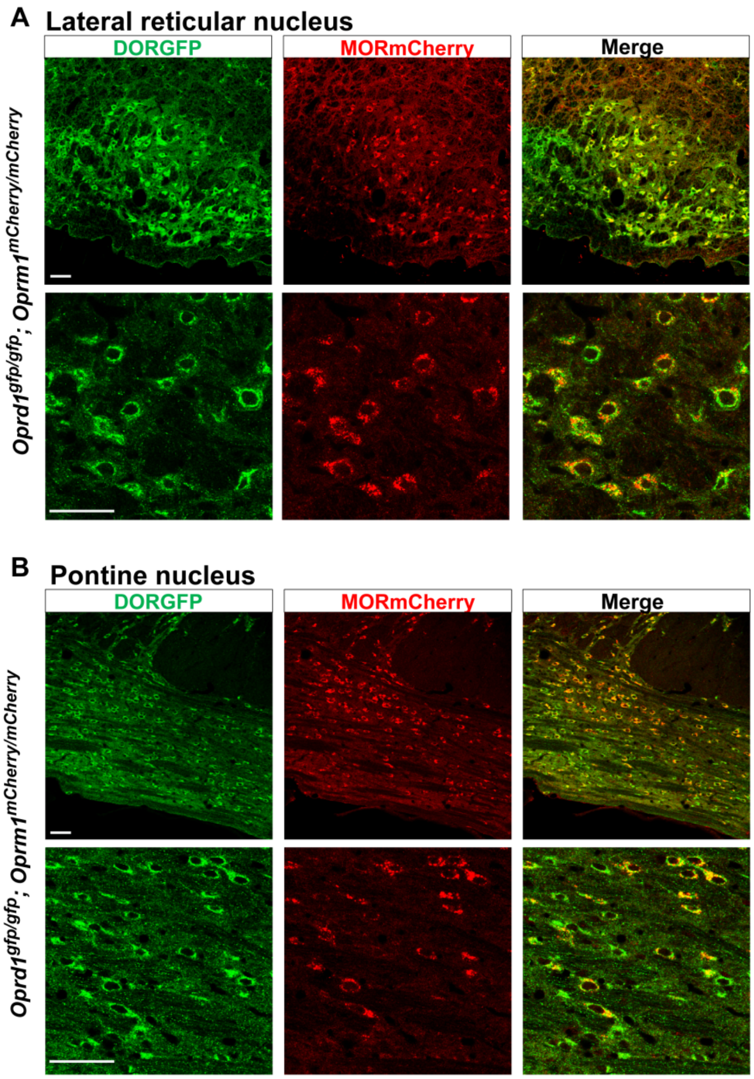
A
Lateral reticular nucleus
DORGFP
MORmCherry
Merge
Oprd1gfp/gfp; Oprm1mCherry/mCherry
B
Pontine nucleus
DORGFP
MORmCherry
Merge
Oprd1gfp/gfp; Oprm1mCherry/mCherry

Figure S7

Figure S7. Co-expression of DOR and MOR in Pre-cerebellar Nuclei.

(Related to Figure 7)

(A) Co-staining with anti-GFP and -RFP antibodies in brain sections from DORGFP;MORmCherry mice indicate DOR and MOR are co-expressed by lateral reticular nucleus (LRt) neurons projecting to the cerebellum.

(B) Co-expression of DOR and MOR in pontine nucleus neurons projecting to the cerebellum.

Scale bars represent 50 μM.

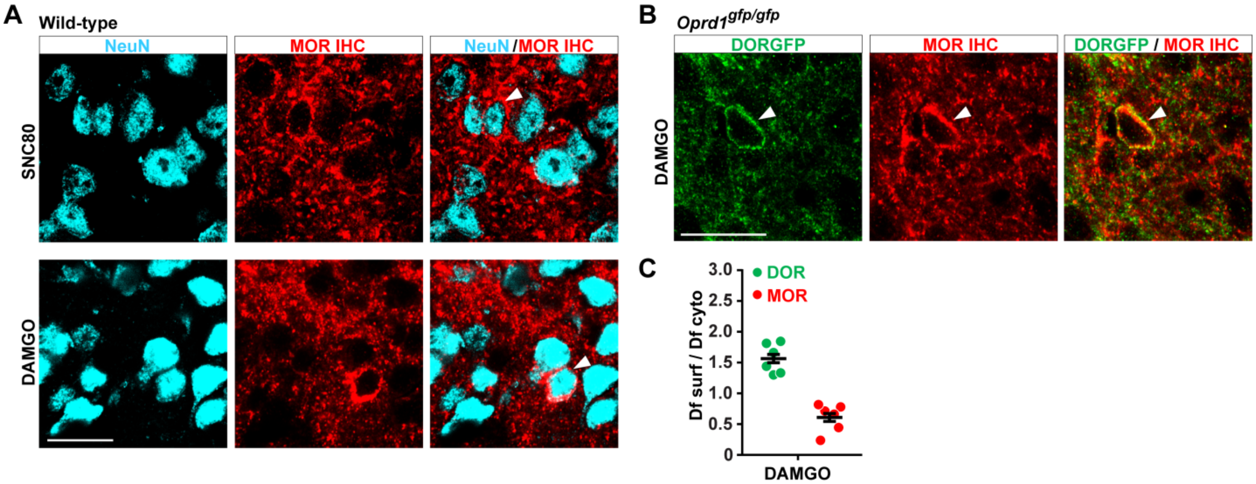
A
Wild-type
NeuN
MOR IHC
NeuN /MOR IHC
SNC80
DAMGO
B
Oprd1gfp/gfp
DORGFP
MOR IHC
DORGFP / MOR IHC
DAMGO
C
DOR
MOR
Df surf / Df cyto
3.0
2.5
2.0
1.5
1.0
0.5
0
DAMGO

Figure S8

Figure S8. The DOR Agonist SNC80 Does Not Cause MOR Co-internalization with DOR in Wild-type Mice.

(Related to Figure 8)

(A) Co-labeling of NeuN and MOR in sections from wild-type mice showing that MOR is internalized by its agonist DAMGO (100 ng, i.t.), but not co-trafficked to lysosomes with DOR following SNC80 treatment. White arrowheads show examples of MOR+ lamina II neurons.

(B) Co-staining with anti-GFP and anti-MOR antibodies in spinal cord sections from DORGFP mice shows that DAMGO causes MOR, without co-internalizing DOR. White arrowheads indicate DORGFP+ MOR+ neurons.

(C) Quantification of (B). Data are presented as mean ± SEM with dots showing individual neurons.

Scale bars represent 20 μM.

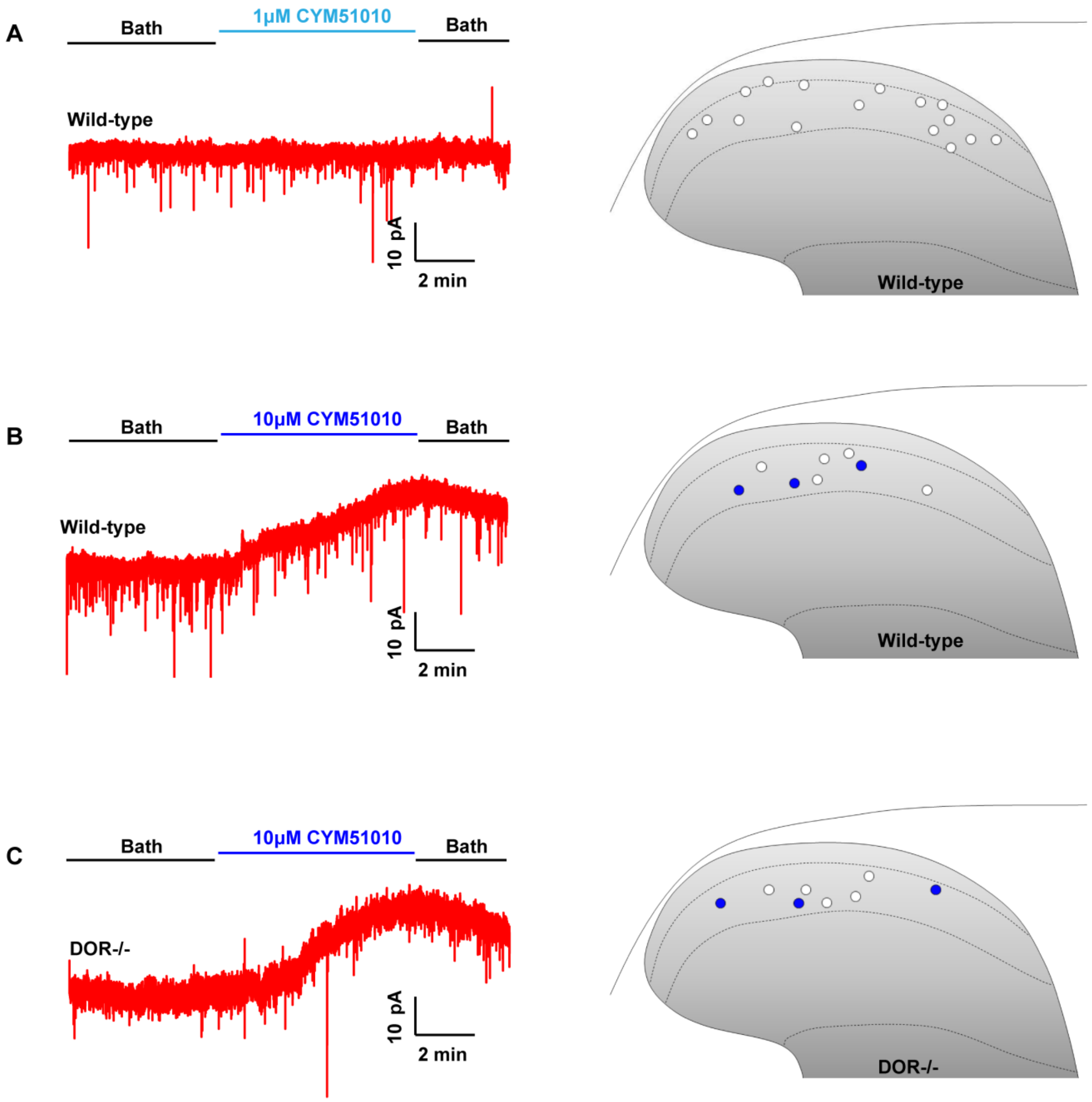
A
Bath
1μM CYM51010
Bath
Wild-type
10 pA
2 min
Wild-type
B
Bath
10μM CYM51010
Bath
Wild-type
10 pA
2 min
Wild-type
C
Bath
10μM CYM51010
Bath
DOR-/-
10 pA
2 min
DOR-/-

Figure S9

Figure S9. Effect of the Biased DOR-MOR Heteromer Agonist On GIRK Channel Currents in Wild-type and DOR KO Mice.

(Related to Figure 8)

(A) Representative trace and schematic map showing that at a dose of 1 μM, the biased DOR-MOR heteromer agonist CYM51010 does not activate GIRK channels in spinal cord dorsal horn neurons in slice from wild-type mice.

(B) 10 μM of CYM51010 did cause activation of GIRK channels in 3 out of 8 recorded dorsal horn neurons in slices from wild-type mice. Blue dots in the schematic map indicate CYM51010-responsive neurons.

(C) 10 μM of CYM51010 also activates GIRK channels in dorsal horn neurons from DOR KO mice, indicating that this response in not mediated by DOR-MOR heteromers, and suggesting that CYM51010 cannot be used to specifically study the neurophysiology of heteromers in slices.
